# Older Europeans’ health perception and their adaptive behavior during the COVID-19 pandemic

**DOI:** 10.1093/eurpub/ckab221

**Published:** 2022-01-03

**Authors:** Sonja Spitzer, Mujaheed Shaikh, Daniela Weber

**Affiliations:** 1 Department of Demography, University of Vienna, Wittgenstein Centre for Demography and Global Human Capital (IIASA, OeAW, University of Vienna), Vienna, 1030, Austria; 2 Political Economy Cluster, Hertie School, Berlin, 10117, Germany; 3 Health Economics and Policy Division, Vienna University of Economics and Business, Vienna, 1020, Austria; 4 International Institute for Applied Systems Analysis (IIASA), Wittgenstein Centre for Demography and Global Human Capital (IIASA, OeAW, University of Vienna), Laxenburg, 2361, Austria

**Keywords:** SARS-CoV-2, biased health beliefs, preventive behaviour, policy guidelines, Survey of Health, Ageing and Retirement in Europe (SHARE)

## Abstract

**Background:**

Although older adults are more vulnerable to the COVID-19 virus, a significant proportion of them do not follow recommended guidelines concerning preventive actions during the ongoing pandemic. This paper analyses the role of biased health beliefs for adaptive health behaviour such as reduced mobility, protection in public spaces, and hygiene measures, for the population aged 50 and older in 13 European countries.

**Methods:**

Health perception is measured based on the difference between self-reported health and physical performance tests for over 24,000 individuals included in the most recent Survey of Health, Ageing and Retirement in Europe. Logistic regressions are employed to explore how over- and underestimating health are related to preventive behaviours.

**Results:**

Results suggest that older adults who underestimate their health are more likely to show adaptive behaviour related to mobility reductions. In particular, they are more likely to stay at home, shop less, and go for walks less often. By contrast, overestimating health is not significantly associated with reduced mobility. Protective behaviour in public spaces and adopting hygiene measures do not vary systematically between health perception groups.

**Conclusion:**

As health beliefs appear relevant for the adoption of preventive health behaviours related to mobility, they have serious consequences for the health and well-being of older Europeans. While adaptive behaviour helps to contain the virus, exaggerated mobility reduction in those who underestimate their health might be contributing to the already high social isolation and loneliness of older adults during the ongoing pandemic.

## 1 INTRODUCTION

Older adults are disproportionately affected by the COVID-19 pandemic and have significantly higher rates of hospitalisation and mortality (1–4). To prevent further spread of COVID-19 and to combat the related health, economic, and social crises, governments across the world have recommended mitigation behaviours such as social distancing, mask-wearing, and frequent hand-washing.

Recent evidence from surveys, however, shows that a significant proportion of older adults, despite their higher vulnerability, do not follow the recommended guidelines. The Centers for Disease Control and Prevention of the United States (USA) report that as of April 2020, 16% of adults over the age of 60 did not wear a face mask, 25% did not cancel or postpone social activities, and about 14% did not avoid crowded or public places (5). (6) observed shoppers in retail stores in the USA and found that only 57% of older adults wore masks. Similar reports emerged during prior pandemic outbreaks such as the Swine Flu, where 32% of older adults in England, Scotland, and Wales reported not following recommended strategies (7).

In addition to age-related deviation from protective guidelines during COVID-19, recent evidence has also explored the role of individual demographic characteristics such as gender, socioeconomic factors like education and income, and psychological differences such as anxiety and optimism (8). We extend this nascent but highly topical literature by investigating the role of biased health perception in explaining the (non)uptake of preventive actions among Europeans aged at least 50 years. Biased health perception that is, over- or underestimating one's own health, was shown to increase substantially with age (9); it is further relevant for the adoption of risky health behaviours and has serious consequences for health and well-being (10,11). In addition, incorrect beliefs about one's own health can affect one's perception of susceptibility to a disease and how severe that disease will be—which are important elements of preventive action according to the Health Belief Model (12). We hypothesise that, relative to people whose perception is accurate, those who underestimate their health show more adaptive behaviour to preventive action and those who overestimate their health show less. The relationship could operate through increased (decreased) perception of susceptibility and severity for individuals who underestimate (overestimate).

### 2 METHODS

#### 2.1 Data and sample

Our analyses are based on the Survey of Health, Ageing and Retirement in Europe (SHARE) COVID-19 survey, which targets Europeans aged 50 and older (13,14). The survey was conducted between May and August 2020 and provides rich information on health and health behaviour during the pandemic (98.3% of all interviews were conducted in June and July 2020). It constitutes the 8$^{th}$ survey wave of the SHARE panel and can be matched with the previous seven waves, which cover more general information on health, socio-economic status, physical performance tests, and thus health perception. We match information on health behaviour from the COVID-19 survey with data on health and health beliefs from survey wave 5 in 2013 (15,16), which is the most recent wave including physical health perception measures. We use additional information from wave 2 in 2007 to show that health perception is rather stable across waves in Appendix 2 of the Supplementary Material, enabling the usage of two different survey waves for the analysis. Thus far, the SHARE COVID-19 survey has been conducted only once, therefore enabling cross-sectional analyses only.

SHARE data is ex ante harmonised, allowing us to analyse 13 European countries that participated in both the COVID-19 survey and in survey wave 5, namely, Belgium, Czechia,

Denmark, Estonia, France, Germany, Italy, Luxembourg, Slovenia, Spain, Sweden, Switzerland, and the Netherlands. We exclude observations based on proxy respondents: this results in 24,507 Europeans aged at least 50 years in 2013 who participated in both waves 5 and 8. The survey was reviewed and approved by the Ethics Committee of the University of Mannheim and the Ethics Council of the Max Planck Society (17).

#### 2.1.2 Outcome variables: adaptive behaviour

We differentiate between three types of COVID-19–related adaptive behaviours, namely, behaviours related to reduced mobility, protection in public spaces, and hygiene measures. Reduced mobility includes staying at home, doing less shopping, less going for walks, less meetings with more than five people from outside the household, and less visits to or from other family members. Protection in public spaces refers to adaptive behaviour that has often been mandated or recommended by law; in particular, wearing masks in public spaces and keeping distance when outside the home. Hygiene measures include washing, sanitising, or disinfecting hands (for cross-tabulations with health perception, see Table 2; for a detailed sequence of survey questions, see Table A.1 in the Supplementary Material, and for summary statistics Table A.2 in the Supplementary Material). All outcome variables are binary and coded so that 1 represents adaptive behaviour. As some adaptive behaviour variables originally had ordered outcomes and were dichotomised (see Table A.1 in the Supplementary Material for details), we conduct robustness analyses that consider the original outcomes. All robustness analyses and their results are explained and discussed in detail in Appendix 2 of the Supplementary Material.

#### 2.1.3 Explanatory variable: health perception

We operationalise health perception using a well-established measure that considers the difference between subjective and objective health (9,11,18). More specifically, we compare survey respondents' self-reported and tested ability to stand up from a chair. Those whose self-reported ability to stand up from a chair matches their outcome during the chair stand test are considered to have achieved either positive concordance (i.e., they are able to stand up from a chair according to both the self-report and the test) or negative concordance (i.e., they are unable to do this according to both the self-report and the test) – see Table 1 for an overview. Individuals who report being able to stand up from a chair but are unable to do so during a chair stand test are classified as overestimating their health, whereas those who report being unable to stand up from the chair but can do so during the performance test are considered as underestimating their health. This health perception measure is explained and evaluated in detail in (9) and (18).

*Table 1 here*
*(Overview of health perception categories and number of observations per category)*

### 2.2 Estimation strategy

Taking into account the binary nature of the outcome variables, the association between individual health perception and adaptive behaviour is estimated based on the following logistic regression model

$$P(y_i = \text{Adaptation}_i \mid X_i) = \alpha + \beta \text{HEALTH PERCEPTION}_i + \gamma X_i + \delta_c + \mu_w + \varepsilon_{icw}$$

where $y_i$ indicates whether individual $i$ shows adaptive behaviour according to the respective outcome, and $HEALTH\ PERCEPTION_i$ indicates its health belief. The vector of control variables $X_i$ includes age, age squared, gender, and educational attainment. Additionally, we control for health status using variables that indicate if the individual has any chronic diseases, limitations in activities of daily living (ADLs) or instrumental activities of daily living (IADLs), whether the individual is frail according to a frailty index (19,20), and also cognitive ability. For robustness analyses, we further control for the respondent's retirement status, relationship status, level of depressive symptoms, household income, and whether they or anyone close to them has tested positive for the COVID-19 virus (Tables A.9 and A.10). More details on the construction of these control variables are given in subsection 2.2.1.

As the severity of the pandemic as well as mandatory rules relating to it vary between countries and over time, we also include control dummies for the country of residence ($\delta_c$) and for survey interview week ($\mu_w$) in the regression model, and interact them in robustness analyses (Tables A.13 and A.14). Moreover, we utilise country-level data on public response measures to further explore the effect of pandemic-related health policies (Tables A.15 and A.16). This research article, however, does not focus on country differences in the results.

Individuals who are able to stand up from a chair might have very different characteristics from individuals unable to do so, which potentially affects their health behaviour. To account for such heterogeneities and in line with previous analyses (18), we further split our sample based on the tested ability to stand up from a chair. Hence, individuals who underestimate their health are always compared to those with positive concordance (i.e., able to stand up from the chair according to self-reports and performance test), and individuals who overestimate their health are always compared to those with negative concordance (i.e., unable to stand up from a chair according to self-reports and performance test).

The dependent variables are taken from the COVID-19 survey (i.e., wave 8) and all explanatory variables are taken from wave 5 when the performance test was conducted, except for the interview week, which also refers to the COVID-19 survey.

#### 2.2.1 Construction of control variables

We control for a variety of potential confounders in our main analysis and include additional control variables for robustness analyses. Summary statistics for all variables are given in Table A.2 in Appendix 1 of the Supplementary Material.

First, we control for age in single years and also include age squared to account for potential non-linearities. Individuals included in the analysis are aged 50–94, with mean age 62. Second, we control for the survey respondent's gender—52.6% of the survey participants are female. Third, we account for the respondent's highest educational attainment, differentiating between low, medium, and high education according to the International Standard Classification of Education (ISCED). Fourth, underlying health status does not confound our findings, as we control for physical and cognitive health in multiple ways. We include a binary variable that indicates whether the survey respondent is suffering from at least one of the following chronic diseases: heart attack, high blood pressure or hypertension, high blood cholesterol, a stroke or cerebral vascular disease, diabetes or high blood sugar, chronic lung disease such as chronic bronchitis or emphysema, cancer or malignant tumour, stomach or duodenal ulcer, Parkinson's Disease, cataracts, hip fracture, other fractures, Alzheimer's Disease, dementia, organic brain syndrome, senility or any other serious memory impairment, other affective or emotional disorders, including anxiety, nervous, or psychiatric problems, rheumatoid arthritis, osteoarthritis, or other forms of rheumatism. In our sample, 67% suffer from at least one of these conditions.

We also include a binary variable that reflects whether the respondent has any limitations in activities of daily living (ADLs: dressing, eating, using the toilet, bathing and showering, getting in and out of bed, walking across a room) and instrumental activities of daily living (IADLs: preparing a hot meal, shopping for groceries, making telephone calls, taking medications, managing money, such as paying bills and keeping track of expenses). In the sample, 7.1% of the respondents have at least one limitation in ADLs, and 9.6% have at least one limitation in IADLs.

A well-established frailty indicator is also included in the regression equation (19). It considers exhaustion, shrinking, weakness, slowness, and low physical activity and was adapted for SHARE in (20), where it is explained in detail. According to this indicator, 7.5% of the sample are considered frail. In addition to physical health, we also account for cognitive ability by adding a control variable that describes orientation to date. This is a categorical variable ranging from zero to four, which measures if the survey respondent knows the day of the week, day of the month, months, and year at the time of the survey interview. The average score across the sample is 3.75.

For robustness analyses, we also control for the individual's retirement status, relationship status, depressive symptoms, whether they or anyone close to them has tested positive for the COVID-19 virus, and the lowest monthly household income during the pandemic. For the retirement status, we create a binary variable that is one if the observation is retired, including semi-retired, partially retired, or in early retirement. According to this variable, 40.0% of the sample are in retirement. We also account for the relationship status with a variable that is one if the individual is living with their partner. This variable is chosen as it includes non-married couples but excludes older adults that do not live together anymore, for example, because one spouse has moved to a nursing home. A total of 67.6% of the participants are living with their partner. We also add a variable indicating if the respondent felt sad or depressed in the month prior to the interview—29.1% of the sample showed depressive symptoms. We also add a variable that reflects whether the respondent or anyone close to the respondent has tested positive for the COVID-19 virus, as such a diagnosis is likely to affect adaptive behaviour during the pandemic. This dummy variable is one for 10.8% of the respondents.

Finally, we control for the household's lowest overall monthly income since the outbreak of the pandemic. In particular, the survey respondent is asked "What was the lowest overall monthly income, after taxes and contributions, that your entire household had, including any financial support you may have received since the outbreak of Corona?" We equivalise the reported income using the square root scale, for which household income is divided by the square root of household size. To account for the skewed income distribution, we further apply a cube root transformation to this variable.

#### 2.2.2 Summary of robustness analyses

We provide a range of robustness analyses that are explained in detail in Appendix 2 of the Supplementary Material and that consider different specifications of health perception, different estimation methods, additional control variables, and heterogeneity analyses. First, we analyse how time-constant health beliefs are across waves. Second, as some adaptive behaviour variables originally had ordered outcomes and were dichotomised (Table A.1 of the Supplementary Material), we conduct robustness analyses considering the original outcomes and run ordered logit regressions instead of logistic regressions (Tables A.5 and A.6). Third, for the original health perception variable, individuals who had to use their arm to help them stand up from the chair during the performance test were considered unable (see 9 and 18 for details on the health perception measure). For the robustness analyses in Tables A.7 and A.8, these individuals are considered able to stand up from a chair. Fourth, we also consider whether respondents are retired, living in a partnership, suffer from depression, and whether they or anyone close to them has tested positive for the COVID-19 virus using additional control variables (Tables A.9 and A.10). We also control for the lowest equivalised monthly household income during the pandemic but we do this in a separate regression, as this variable is missing for over 23% of the survey respondents (Tables A.11 and A.12). Finally, we also interact the country and survey week dummies (Tables A.13 and A.14), and control for pandemic-related public health policies at the time of the interview (Tables A.15 and A.16) to further account for variations between countries and over time in the severity of the pandemic as well as in the mandatory rules.

### 3 RESULTS

Column 1 in Table 2 provides descriptive statistics, which suggest that most older adults engage in preventive actions, especially with respect to protection in public spaces and hygiene measures. The most common preventive behaviours are keeping distance (96.1%) and fewer meetings (91.1%), while only a small proportion of individuals stayed at home during the pandemic (12.1%). Adaptive behaviour varies, however, across health perception groups (Columns 2–5, Table 2). Most importantly, individuals who underestimated their health reduced their mobility more and were more likely to wear masks than their comparison groups (i.e., positive concordance). The pattern is less clear for other types of protection in public spaces and hygiene measures, and also among those who overestimated their health.

Table 2 here (Cross-tabulations of health perception and COVID-19–related adaptive behaviour)

Figure 1 shows the logistic regression results, which confirm the descriptive findings for mobility reductions. Relative to older adults who accurately assess their health, those who underestimate are significantly more likely to stay at home, more likely to reduce shopping, and more likely to reduce going for walks. No significant association between underestimation and protection in public spaces or hygiene measures is found. Contrary to our hypothesis, those who overestimate their health are significantly more likely to keep distance when outside their home. We do not, however, find a statistically significant relationship between overestimation of health and any of the other preventive behaviours.

*Figure 1 here*

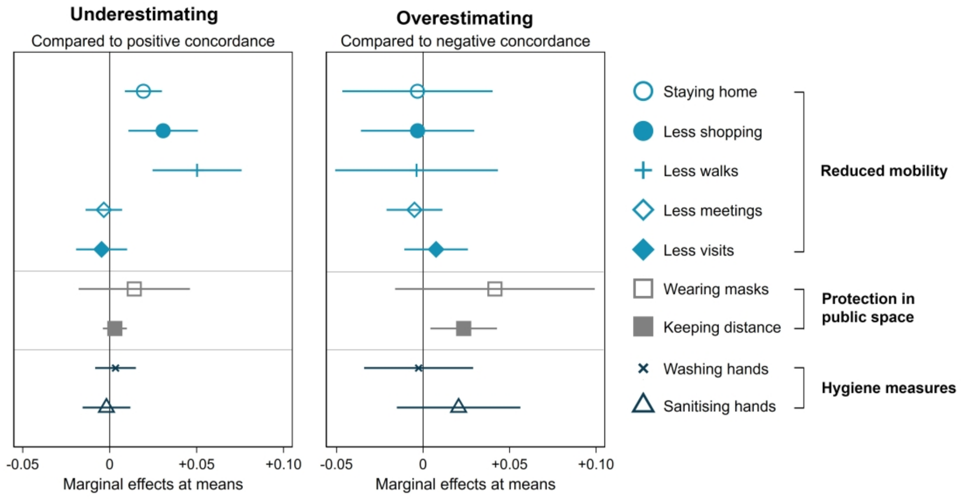

Figure 1: Marginal effects (at means) of health perception on Covid-19–related adaptive behaviour (the underlying estimation results, including coefficients and standard errors, are provided in Tables A.3 and A.4 in Appendix 1 of the Supplementary Material)

Note that underlying health status does not confound these findings, as we control for physical and cognitive health in multiple ways and split the sample based on the tested ability to stand up from a chair. Additionally, we control for differences in adaptive pandemic behaviour by age, gender, and education. Tables A.3 and A.4 in Appendix 1 of the Supplementary Material show the full set of coefficients. In addition to our main results, we find that women —who overall are more likely to underestimate their health (12.5%) than men (6.5%)— engage significantly more in adaptive behaviours. Moreover, preventive health behaviour increases with higher age for most measures, and older adults with higher education are more likely to adopt protection in public spaces and hygiene measures but less likely to reduce their mobility, compared to the less educated.

Our results are robust to a range of robustness analyses, described in Appendix 2 of the Supplementary Material, that consider different specifications of health perception, different estimation methods, additional control variables, and interactions.

### 4 DISCUSSION

Although older adults are more vulnerable during the COVID-19 pandemic (2–4,21) and perceive the risk of the virus to be higher than younger adults do (22), studies frequently show that older adults fail to engage in protective health behaviour (6–8). One explanation might be the increase in health misperception with age (9), leading to deviations from recommended guidelines. In this paper, we hypothesised that individuals underestimating their health engage more in preventive action, while individuals overestimating their health engage less.

Our results show that older adults who underestimate their health are indeed more likely to exercise adaptive behaviour, at least concerning mobility reductions. More specifically, they are more likely to stay at home, shop less, and go for walks less often. The relationship might operate through increased perception of disease susceptibility and severity (12). While these preventive actions may protect individuals and help to contain the virus, an exaggerated reduction in mobility on the part of those who underestimate their health might be contributing to the already high social isolation and loneliness of older adults during the ongoing pandemic (23–25). In additional analyses, we also find that depressive symptoms are associated with an increase in adaptive behaviour (Tables A.9 and A.10).

Contrary to our hypothesis, adaptive behaviour related to protection in public spaces and hygiene measures are not significantly associated with underestimating health. One possible explanation is that protection in public spaces was often mandatory and was thus exercised regardless of health perception. Overall, we find that differences in health perception matter less for preventive behaviours that are widely adopted, such as hygiene measures (Table 2). Moreover, the SHARE survey questions on hygiene measures are phrased so that they ask whether respondents washed, sanitised, or disinfected their hands more frequently than usual, (i.e. if they increased their hygiene behaviour) (Table A.1 in the Supplementary Material). If individuals who underestimate their health had already adopted such hygiene measures before the pandemic, their higher baseline level could explain why they did not (have to) further increase their hygiene measures, which is why we find no effect of underestimating health on hygiene measures during the pandemic.

Overestimating health appears not to be significantly associated with preventive behaviour. The only exception is that those who overestimate their health appear more likely to keep distance when outside, which is contrary to our hypothesis. Speculatively, those who overestimate their health might also be overconfident in other domains (i.e., their preventive behaviour) or even overestimate distances themselves, which could explain this curious finding. Moreover, the positive effect becomes insignificant once the original ordered structure of the outcome variable is considered by employing ordered logit regressions instead of logistic regressions for binary outcomes (Table A.6).

In addition to our main results, we find that women in good health are more likely to engage in protective behaviours than men. This is in line with recent studies showing greater COVID-

19–related risk awareness among women (22,26), who in turn may be more likely to implement protective behaviour (27).

One potential limitation of this study is that data on health perception is taken from 2013, because the SHARE COVID-19 study did not cover the performance tests needed for the health perception measure. We have shown, however, that individual health perception is generally stable over time and we also control for age as a potential confounder. Furthermore, this analysis does not elicit the causal effects of health perception on adaptive behaviour, which could be desirable for future work.

In conclusion, our study showed that health perception is relevant for the adoption of preventive health behaviour among older Europeans, especially with respect to mobility reductions. Future work could fruitfully explore a potential link between underestimating health, mobility reduction, and increases in social isolation and loneliness.

### FUNDING

This work was supported by the Austrian Academy of Sciences via an APART-GSK Fellowship for Daniela Weber. This paper uses data from SHARE Waves 2, 5, and 8 (10.6103/SHARE.w2.710, 10.6103/SHARE.w5.710, 10.6103/SHARE.w8ca.100). The SHARE data collection has been funded by the European Commission, DG RTD through FP5 (QLK6-CT-2001-00360), FP6 (SHARE-I3: RII-CT-2006-062193, COMPARE: CIT5-CT-2005-028857, SHARELIFE: CIT4-CT-2006-028812), FP7 (SHARE-PREP: GA N°211909, SHARE-LEAP: GA N°227822, SHARE M4: GA N°261982, DASISH: GA N°283646) and Horizon 2020 (SHARE-DEV3: GA N°676536, SHARE-COHESION: GA N°870628, SERISS: GA N°654221, SSHOC: GA N°823782) and by DG Employment, Social Affairs &

Inclusion through VS 2015/0195, VS 2016/0135, VS 2018/0285, VS 2019/0332, and VS 2020/0313. Additional funding from the German Ministry of Education and Research, the Max Planck Society for the Advancement of Science, the U.S. National Institute on Aging (U01_AG09740-13S2, P01_AG005842, P01_AG08291, P30_AG12815, R21_AG025169, Y1-AG-4553-01, IAG_BSR06-11, OGHA_04-064, HHSN271201300071C, RAG052527A) and from various national funding sources is gratefully acknowledged (see www.share-project.org).

### CONFLICT OF INTEREST

None declared

### KEY POINTS

- A significant proportion of older adults do not follow recommended guidelines concerning preventive actions during the COVID-19 pandemic.
- Health beliefs appear relevant for the adoption of preventive health behaviours related to mobility, and thus have serious consequences for the containment of the virus.
- Older adults who underestimate their health are more likely to show adaptive behaviour related to mobility reductions.
- Overestimating health is not significantly associated with reduced mobility, protective behaviour in public spaces, or hygiene measures.

### DATA AVAILABILITY

The data underlying the results presented in the study are available from the Survey of Health, Ageing and Retirement in Europe (http://www.share-project.org).

### ONLINE SUPPELEMENTARY MATERIAL

The Supplementary Material will be made available online and is currently uploaded as a separate document for the submission process.

Table 1: Overview of health perception categories and number of observations per category

| | **Objectively** | | | |
|---|---|---|---|---|
| **Subjectively** | Able | | Unable | |
| Able | Positive concordance | 18,746 (87.2%) | Overestimating | 1,416 (47.1%) |
| Unable | Underestimating | 2,756 (12.8%) | Negative concordance | 1,589 (52.9%) |
| **Total** | | **21,502 (100%)** | | **3,005 (100%)** |

Note: No weights applied

Table 2: Cross-tabulations of health perception and COVID-19–related adaptive behaviour

| | (1) | (2) | (3) | (4) | (5) |
|---|---|---|---|---|---|
| | **Total** | **Positive concor-dance** | **Under-estimating** | **Negative concor-dance** | **Over-estimating** |
| | (%) | (%) | (%) | (%) | (%) |
| **Reduced mobility** | | | | | |
| Staying home | 12.1 | 9.9 | 14.3 | 31.8 | 21.8 |
| Less shopping | 69.2 | 67.4 | 70.6 | 83.8 | 79.4 |
| Less walks | 52.2 | 49.3 | 54.8 | 72.5 | 71.1 |
| Less meetings | 91.1 | 90.5 | 92.2 | 93.5 | 94.3 |
| Less visit | 84.0 | 83.3 | 84.1 | 88.1 | 89.8 |
| **Protection in public space** | | | | | |
| Wearing masks | 79.3 | 78.7 | 80.9 | 82.5 | 84.0 |
| Keeping distance | 96.1 | 96.3 | 95.6 | 94.1 | 96.4 |
| **Hygiene measures** | | | | | |
| Washing hands | 87.2 | 88.2 | 84.8 | 76.9 | 86.4 |
| Sanitising hands | 80.6 | 81.3 | 77.6 | 73.2 | 83.2 |

Note: Calibrated cross-sectional individual weights are applied; in Column 1, 100% refers to the total number of observations; in Columns 2–5, 100% refers to the number of observations in the respective health perception category; variables "less shopping", "less walks", "less meetings" and "less visits" are only asked to those who did not stay at home and thus for these variables the category "adaptation" includes the share of individuals who did not leave their home (for more details see Table A.1 in the Supplementary Material)

**Older Europeans' health perception and their adaptive behaviour during the COVID-19 pandemic**

**SUPPLEMENTARY MATERIAL**

**Appendix 1: main results**

Table A.1 Sequence of survey questions for outcome variables

| | **Survey question** | **Possible outcomes** | **Routing** | **Transformation** |
|---|---|---|---|---|
| 1 | Since the outbreak of Corona, have you ever left your home? | • Yes<br>• No | Asked to entire sample | • Adaptation: no<br>• No adaptation: yes |
| 2 | Since the outbreak of Corona, how often have you done the following activities, as compared to before the outbreak?<br>• Going shopping?<br>• Going out for a walk?<br>• Meeting with more than five people from outside your household?<br>• Visiting other family members? | • Not anymore<br>• Less often<br>• About the same<br>• More often | Asked if question 1 is answered with "yes" | • Adaptation: not anymore, less often, if question 1 is answered with "no"<br>• No adaptation: about the same, more often |
| 3 | How often did you wear a face mask when you went outside your home to a public space? | • Always<br>• Often<br>• Sometimes<br>• Never | Asked if question 1 is answered with "yes" | • Adaptation: always, often<br>• No adaptation: sometimes, never |
| 4 | How often did you keep distance to others when you went outside your home? | • Always<br>• Often<br>• Sometimes<br>• Never | Asked if question 1 is answered with "yes" | • Adaptation: always, often<br>• No adaptation: sometimes, never |
| 5 | Did you wash your hands more frequently than usual? | • Yes | Asked to entire sample | • Adaptation: yes |

| | | | | |
|---|---|---|---|---|
| | | • No | | • No adaptation: no |
| 6 | Did you use special hand sanitiser or disinfection fluids more frequently than usual? | • Yes<br>• No | Asked to entire sample | • Adaptation: yes<br>• No adaptation: no |

Table A.2: Summary statistics

| | N | Mean | SD | Min. | Max. |
|---|---|---|---|---|---|
| **Adaptive behaviour** | | | | | |
| **Reduced mobility** | | | | | |
| Staying home (1 = yes) | 24,399 | 0.121 | 0.326 | 0 | 1 |
| Less shopping (1 = yes) | 24,164 | 0.692 | 0.462 | 0 | 1 |
| Less walks (1 = yes) | 24,060 | 0.522 | 0.500 | 0 | 1 |
| Less meetings (1 = yes) | 23,909 | 0.911 | 0.285 | 0 | 1 |
| Less visits (1 = yes) | 23,944 | 0.840 | 0.367 | 0 | 1 |
| **Protection in public spaces** | | | | | |
| Wearing masks (1 = yes) | 20,686 | 0.793 | 0.405 | 0 | 1 |
| Keeping distance (1 = yes) | 20,654 | 0.961 | 0.193 | 0 | 1 |
| **Hygiene measures** | | | | | |
| Washing hands (1 = yes) | 24,364 | 0.872 | 0.334 | 0 | 1 |
| Sanitising hands (1 = yes) | 24,373 | 0.806 | 0.395 | 0 | 1 |
| **Health perception** | | | | | |
| Positive concordance (1 = yes) | 24,507 | 0.796 | 0.403 | 0 | 1 |
| Underestimating (1 = yes) | 24,507 | 0.097 | 0.296 | 0 | 1 |
| Negative concordance (1 = yes) | 24,507 | 0.047 | 0.212 | 0 | 1 |
| Overestimating (1 = yes) | 24,507 | 0.060 | 0.238 | 0 | 1 |
| **Mobility** | | | | | |
| Objectively mobile (1 = yes) | 24,507 | 0.893 | 0.310 | 0 | 1 |
| Subjectively mobile (1 = yes) | 24,507 | 0.856 | 0.351 | 0 | 1 |
| **Control variables** | | | | | |
| Age (in number of years) | 24,507 | 61.896 | 9.726 | 50 | 94 |
| Gender (1 = female) | 24,507 | 0.526 | 0.499 | 0 | 1 |
| Low education | 24,162 | 0.363 | 0.481 | 0 | 1 |
| Medium education | 24,162 | 0.393 | 0.488 | 0 | 1 |
| High education | 24,162 | 0.244 | 0.430 | 0 | 1 |
| Chronic disease dummy (1 = yes) | 24,499 | 0.674 | 0.469 | 0 | 1 |
| ADL dummy (1 = yes) | 24,505 | 0.071 | 0.258 | 0 | 1 |
| IADL dummy (1 = yes) | 24,505 | 0.096 | 0.295 | 0 | 1 |
| Frailty (1 = frail) | 23,212 | 0.075 | 0.263 | 0 | 1 |
| Cognitive ability (4 = high) | 24,482 | 3.749 | 0.587 | 0 | 4 |
| Retired (1 = yes) | 24,446 | 0.400 | 0.490 | 0 | 1 |
| Living with partner (1 = yes) | 24,276 | 0.676 | 0.468 | 0 | 1 |
| Feeling depressed or sad (1 = yes) | 24,445 | 0.291 | 0.455 | 0 | 1 |
| Anyone tested positive (1 = yes) | 24,197 | 0.108 | 0.310 | 0 | 1 |
| Lowest monthly household income | 18,612 | 1,802 | 1,525 | 0 | 82,704 |

Note: Calibrated cross-sectional individual weights are applied to all columns except "N"

Table A.3: Main results for underestimating health

| | (1) Staying home | (2) Less shopping | (3) Less walks | (4) Less meetings | (5) Less visits | (6) Wearing masks | (7) Keeping distance | (8) Washing hands | (9) Sanitising hands |
|---|---|---|---|---|---|---|---|---|---|
| Underestimating | 0.255*** | 0.167** | 0.202*** | -0.054 | -0.044 | 0.070 | 0.106 | 0.040 | -0.016 |
| | (0.066) | (0.057) | (0.052) | (0.085) | (0.070) | (0.082) | (0.130) | (0.071) | (0.061) |
| Age | -0.023 | -0.041 | -0.113*** | 0.208*** | 0.136*** | 0.116* | 0.241*** | 0.189*** | 0.083** |
| | (0.039) | (0.030) | (0.030) | (0.044) | (0.036) | (0.046) | (0.058) | (0.035) | (0.032) |
| Age squared | 0.001* | 0.001* | 0.001*** | -0.001*** | -0.001*** | -0.001* | -0.002*** | -0.002*** | -0.001*** |
| | (0.000) | (0.000) | (0.000) | (0.000) | (0.000) | (0.000) | (0.000) | (0.000) | (0.000) |
| Women | 0.264*** | 0.644*** | 0.268*** | 0.406*** | 0.324*** | 0.413*** | 0.408*** | 0.224*** | 0.118** |
| | (0.045) | (0.032) | (0.031) | (0.049) | (0.040) | (0.045) | (0.075) | (0.044) | (0.037) |
| Medium education (ref. = low) | -0.324*** | -0.041 | -0.210*** | -0.036 | -0.073 | 0.168* | 0.132 | 0.075 | 0.116* |
| | (0.057) | (0.045) | (0.042) | (0.066) | (0.056) | (0.066) | (0.094) | (0.057) | (0.051) |
| High education (ref. = low) | -0.647*** | 0.030 | -0.428*** | 0.185* | 0.015 | 0.368*** | 0.465*** | 0.327*** | 0.192*** |
| | (0.073) | (0.049) | (0.047) | (0.076) | (0.062) | (0.071) | (0.113) | (0.067) | (0.058) |
| Chronic disease dummy | 0.082 | 0.181*** | 0.241*** | 0.079 | 0.096* | 0.238*** | 0.154 | -0.031 | 0.067 |
| | (0.055) | (0.037) | (0.037) | (0.055) | (0.046) | (0.053) | (0.086) | (0.052) | (0.046) |
| ADL dummy | -0.053 | 0.029 | 0.216** | 0.181 | 0.304** | -0.071 | -0.295 | -0.023 | 0.011 |
| | (0.104) | (0.087) | (0.081) | (0.136) | (0.118) | (0.119) | (0.179) | (0.101) | (0.090) |
| IADL dummy | 0.291*** | 0.144 | 0.250*** | 0.021 | 0.054 | -0.043 | -0.143 | -0.238** | -0.049 |
| | (0.082) | (0.074) | (0.069) | (0.112) | (0.093) | (0.099) | (0.158) | (0.083) | (0.077) |
| Frailty dummy | 0.472*** | 0.217* | 0.645*** | 0.642*** | 0.306* | 0.287* | -0.211 | -0.121 | -0.303*** |
| | (0.085) | (0.097) | (0.089) | (0.173) | (0.128) | (0.135) | (0.184) | (0.101) | (0.087) |
| Cognition score = 1 (ref. = 0) | -0.129 | 0.252 | -0.480 | -0.127 | -0.528 | -0.010 | -1.519* | -0.401 | -0.673 |
| | (0.388) | (0.472) | (0.479) | (0.809) | (0.569) | (0.800) | (0.753) | (0.381) | (0.363) |
| Cognition score = 2 (ref. = 0) | -0.749** | -0.170 | -0.615* | -0.430 | -0.241 | -0.194 | -0.734 | 0.213 | -0.130 |
| | (0.270) | (0.283) | (0.310) | (0.427) | (0.382) | (0.461) | (0.581) | (0.270) | (0.267) |

| | | | | | | | | | |
|---|---|---|---|---|---|---|---|---|---|
| Cognition score = 3 (ref. = 0) | -0.925*** | -0.192 | -0.723** | -0.369 | -0.339 | 0.115 | -0.210 | 0.660** | 0.122 |
| | (0.225) | (0.246) | (0.272) | (0.363) | (0.331) | (0.402) | (0.524) | (0.225) | (0.229) |
| Cognition score = 4 (ref. = 0) | -1.033*** | -0.205 | -0.774** | -0.380 | -0.417 | 0.209 | -0.154 | 0.685** | 0.225 |
| | (0.220) | (0.244) | (0.270) | (0.358) | (0.327) | (0.397) | (0.516) | (0.220) | (0.225) |
| Constant | -3.721** | 0.494 | 2.056* | -5.304*** | -3.483** | -1.763 | -4.107* | -4.554*** | -0.956 |
| | (1.332) | (1.011) | (1.000) | (1.451) | (1.192) | (1.549) | (2.001) | (1.169) | (1.080) |
| Country dummies | Yes | Yes | Yes | Yes | Yes | Yes | Yes | Yes | Yes |
| Interview week dummies | Yes | Yes | Yes | Yes | Yes | Yes | Yes | Yes | Yes |
| N | 20,510 | 20,331 | 20,242 | 20,104 | 20,141 | 17,901 | 17,880 | 20,488 | 20,496 |
| Pseudo R2 | 0.184 | 0.092 | 0.210 | 0.080 | 0.060 | 0.524 | 0.047 | 0.035 | 0.068 |

Table A.4: Main results for overestimating health

| | (1) Staying home | (2) Less shopping | (3) Less walks | (4) Less meetings | (5) Less visits | (6) Wearing masks | (7) Keeping distance | (8) Washing hands | (9) Sanitising hands |
|---|---|---|---|---|---|---|---|---|---|
| Overestimating | -0.018 | -0.027 | -0.019 | -0.129 | 0.149 | 0.255 | 0.614* | -0.021 | 0.142 |
| | (0.126) | (0.140) | (0.123) | (0.217) | (0.183) | (0.177) | (0.249) | (0.133) | (0.125) |
| Age | 0.026 | -0.077 | -0.081 | 0.089 | 0.236* | 0.061 | 0.379** | 0.117 | 0.106 |
| | (0.085) | (0.097) | (0.080) | (0.132) | (0.105) | (0.129) | (0.143) | (0.084) | (0.078) |
| Age squared | 0.000 | 0.001 | 0.001 | -0.000 | -0.002* | -0.001 | -0.003** | -0.001 | -0.001* |
| | (0.001) | (0.001) | (0.001) | (0.001) | (0.001) | (0.001) | (0.001) | (0.001) | (0.001) |
| Women | 0.479*** | 0.709*** | 0.424*** | 0.290 | 0.165 | 0.587*** | 0.552* | 0.386** | 0.200 |
| | (0.120) | (0.122) | (0.109) | (0.193) | (0.164) | (0.164) | (0.221) | (0.123) | (0.116) |
| Medium education (ref. = low) | -0.382** | -0.127 | 0.065 | -0.088 | 0.046 | -0.172 | 0.440 | -0.259 | 0.231 |
| | (0.135) | (0.145) | (0.125) | (0.224) | (0.195) | (0.191) | (0.245) | (0.146) | (0.134) |
| High education (ref. = low) | -0.594** | 0.055 | -0.176 | 0.170 | -0.215 | 0.090 | 0.246 | -0.089 | 0.203 |
| | (0.184) | (0.183) | (0.154) | (0.292) | (0.238) | (0.223) | (0.319) | (0.184) | (0.173) |
| Chronic disease dummy | -0.007 | 0.153 | 0.187 | 0.576* | 0.002 | 0.315 | 0.079 | 0.129 | 0.147 |
| | (0.164) | (0.168) | (0.150) | (0.230) | (0.236) | (0.213) | (0.295) | (0.175) | (0.164) |
| ADL dummy | 0.331* | 0.402* | 0.288 | 0.470 | -0.023 | 0.299 | 0.610* | -0.079 | -0.163 |
| | (0.140) | (0.187) | (0.158) | (0.304) | (0.239) | (0.231) | (0.308) | (0.143) | (0.140) |
| IADL dummy | 0.328* | 0.395* | 0.318* | 0.113 | 0.274 | -0.174 | -0.100 | -0.306* | -0.100 |
| | (0.134) | (0.175) | (0.144) | (0.276) | (0.244) | (0.206) | (0.268) | (0.145) | (0.140) |
| Frailty dummy | 0.255* | 0.290 | 0.502*** | 0.068 | 0.276 | -0.081 | -0.205 | -0.294* | 0.051 |
| | (0.129) | (0.165) | (0.137) | (0.248) | (0.211) | (0.198) | (0.256) | (0.137) | (0.128) |
| Cognition score = 1 (ref. = 0) | -0.471 | 0.675 | 0.182 | 0.005 | -0.662 | -0.398 | -0.880 | -0.290 | 0.379 |
| | (0.581) | (1.220) | (0.858) | (1.140) | (1.291) | (1.119) | (1.347) | (0.619) | (0.613) |
| Cognition score = 2 (ref. = 0) | -0.427 | 0.732 | 0.477 | 0.579 | 0.045 | 0.846 | 0.000 | 0.117 | 0.195 |
| | (0.446) | (0.738) | (0.631) | (0.731) | (0.949) | (0.987) | (.) | (0.436) | (0.445) |

| | | | | | | | | | |
|---|---|---|---|---|---|---|---|---|---|
| Cognition score = 3 (ref. = 0) | -1.248** | 0.056 | -0.425 | 0.033 | -0.556 | 0.474 | 0.463 | 0.375 | 0.142 |
| | (0.385) | (0.578) | (0.506) | (0.248) | (0.770) | (0.811) | (0.743) | (0.374) | (0.384) |
| Cognition score = 4 (ref. = 0) | -1.465*** | -0.255 | -0.504 | 0.000 | -0.443 | 0.592 | 0.986 | 0.634 | 0.388 |
| | (0.373) | (0.558) | (0.495) | (.) | (0.757) | (0.790) | (0.709) | (0.359) | (0.372) |
| Constant | -4.995 | 1.555 | 0.583 | -2.657 | -6.809 | 0.105 | -10.359* | -2.177 | -1.905 |
| | (2.952) | (3.198) | (2.700) | (4.321) | (3.561) | (4.327) | (4.932) | (2.903) | (2.675) |
| Country dummies | Yes | Yes | Yes | Yes | Yes | Yes | Yes | Yes | Yes |
| Interview week dummies | Yes | Yes | Yes | Yes | Yes | Yes | Yes | Yes | Yes |
| N | 2,260 | 2,219 | 2,224 | 2,118 | 2,192 | 1,580 | 1,464 | 2,250 | 2,255 |
| Pseudo R2 | 0.240 | 0.155 | 0.218 | 0.103 | 0.123 | 0.444 | 0.128 | 0.061 | 0.069 |

**Appendix 2: robustness analyses**

Our results are robust to a range of robustness analyses that consider different specifications of health perception, different estimation methods, additional control variables including macro drivers of adaptive health, and interactions. First, as some adaptive behaviour variables originally had ordered outcomes and were dichotomised (see Table A.1 in Appendix 1), we conduct robustness analyses considering the original outcomes and run ordered logit regressions instead of logistic regressions for this subset of outcome variables (Tables A.5 and A.6). Although the coefficients for underestimating health vary slightly in magnitude, the results are qualitatively similar to the main analysis. Interestingly, the curious positive effect of overestimating health on keeping distance when outside disappears when ordered logit regressions are employed.

Second, for the original health perception variable, individuals who had to use their arm to help them stand up from the chair during the performance test were considered unable (see 1 or 2 for details on the health perception measure). For the robustness analyses in Tables A.7 and A.8, these individuals are considered able to stand up from a chair. The results are virtually identical to the main analysis.

Third, we also consider additional control variables (Tables A.9 and A.10). Most importantly, we control for depressive symptoms. While the depressive realism hypothesis conjectures that depressive individuals may actually be more accurate and realistic than non-depressed individuals, the selective processing hypothesis suggests that depression may lead to negative bias and under-confidence. Theoretically, the relationship is complex, and experimental evidence adds to this complexity. While a range of early experimental evidence shows that depressed individuals are more realistic than their non-depressed counterparts (3), recent research finds that depressed individuals are slightly more likely to be under-confident in their performance evaluations (4). Furthermore, major cognitive theories of depression point towards a reverse relationship such that negative perceptions of self can cause depression (5,6). It is therefore unclear how depression may actually affect both health perception and adaptive behaviour, or whether the relationship is in the other direction such that staying at home affects depression.

The main results of underestimating and overestimating health on adaptive behaviour remain unchanged when controlling for depression. In addition, we find that individuals with depressive symptoms also appear more likely to show adaptive behaviour. Our results also remain robust to adding retirement status, partnership status, and a variable indicating whether the survey participants or anyone close to them has tested positive for the COVID-19 virus. While living with a partner increases the likelihood of almost all types of preventive behaviour, retirement and positive COVID-19 experiences affect only some of the outcome variables (Tables A.9 and A.10).

Depending on their economic resources, households might be more or less likely to adapt their behaviour to the ongoing pandemic. We thus also control for the lowest equivalised monthly household income during the pandemic but we do this in a separate regression, as this variable is missing for over 23% of the survey respondents (Tables A.11 and A.12). Our main results are, again, robust to this additional control variable. We find, however, that the effect of income on preventive health behaviour varies across measures. While mobility reductions decrease with higher income, protection in public spaces and hygiene measures increase.

Fourth, we also account for differences in the severity of the pandemic as well as in mandatory rules across countries and time. We interact the country and survey week dummies to further control for variations across time and space (Tables A.13 and A.14). The results remain virtually identical to the main analysis. As public health and social measures enforced at the time of the interview might be important macro drivers of adaptive health behaviour, we also control for policy variations across countries and times during the study period. For this robustness analysis, we utilise data from the European Centre for Disease Prevention and Control (7) which includes selected national public response measures to COVID-19 on a weekly basis. In particular, we control for mandatory mask use in public spaces to account for macro drivers of protection in public spaces, and for the restriction of private gatherings to account for macro drivers of reduced mobility. Variables that could proxy macro drivers of hygiene measures are not available, as washing or sanitising hands was not enforced by law but rather recommended or socially regulated.

Results in Tables A.15 to A.16 show that our main findings are robust for the inclusion of health and social measures at the country level (i.e., individuals who underestimate their health are more likely to show adaptive behaviour). Moreover, the macro controls have little effect on individual adaptive health behaviour, most likely because country-level variations are already accounted for due to the inclusion of country dummies and controls for the week of the interview. The only additional effect found suggests that mandatory mask use in public spaces decreases mobility reductions related to shopping, potentially because older adults feel that it is safer to go shopping when mandatory mask use applies.

Finally, we investigate if and how health perception changes over time. For our main analysis, we match information on health and health beliefs from wave 5 in 2013 with data on health behaviour from the COVID-19 survey in 2020. It is thus important to investigate how frequently individuals change their health beliefs between waves. Unfortunately, the 2020 COVID-19 survey does not include tested health variables and thus does not allow us to analyse health beliefs. We can, however, analyse variations in health perception between wave 2 in 2007, which also includes the chair stand test, and wave 5 in 2020.

A total of 13,595 individuals participated in both waves 2 and 5, which is a much smaller and potentially selected sample than the one used for the main analysis. This subsample allows us, however, to explore how health perception varies over time. It appears that health beliefs are constant between the waves for the large majority of observations, namely 61.7%, thus reassuring us that our health perception measure is valid for a large fraction of the sample. The within-individual standard deviation in health perception is only 0.10 for the objectively able sample (individuals that are able to stand up from a chair during the chair stand test) and 0.08 for the objectively unable (individuals that are unable to stand up from a chair during the chair stand test).

| | (1) Less shopping | (2) Less walks | (3) Less meetings | (4) Less visits | (5) Wearing masks | (6) Keeping distance |
|---|---|---|---|---|---|---|
| Underestimating | 0.174*** | 0.241*** | 0.026 | 0.086 | 0.068 | 0.001 |
| | (0.045) | (0.046) | (0.051) | (0.048) | (0.063) | (0.066) |
| Age | -0.177*** | -0.134*** | 0.087** | 0.059* | 0.116*** | 0.155*** |
| | (0.025) | (0.024) | (0.027) | (0.026) | (0.034) | (0.034) |
| Age squared | 0.002*** | 0.001*** | -0.000 | -0.000 | -0.001*** | -0.001*** |
| | (0.000) | (0.000) | (0.000) | (0.000) | (0.000) | (0.000) |
| Women | 0.483*** | 0.120*** | 0.305*** | 0.274*** | 0.429*** | 0.388*** |
| | (0.027) | (0.025) | (0.028) | (0.026) | (0.034) | (0.037) |
| Medium education (ref. = low) | -0.162*** | -0.215*** | -0.120** | -0.134*** | 0.119* | 0.055 |
| | (0.037) | (0.036) | (0.041) | (0.039) | (0.050) | (0.051) |
| High education (ref. = low) | -0.145*** | -0.534*** | -0.179*** | -0.193*** | 0.270*** | 0.076 |
| | (0.040) | (0.040) | (0.044) | (0.041) | (0.054) | (0.055) |
| Chronic disease dummy | 0.153*** | 0.187*** | 0.051 | 0.073* | 0.202*** | 0.046 |
| | (0.030) | (0.030) | (0.033) | (0.031) | (0.040) | (0.043) |
| ADL dummy | 0.063 | 0.157* | 0.082 | 0.111 | -0.053 | -0.141 |
| | (0.073) | (0.072) | (0.079) | (0.074) | (0.099) | (0.098) |
| IADL dummy | 0.308*** | 0.300*** | 0.003 | 0.117 | 0.043 | -0.048 |
| | (0.062) | (0.061) | (0.065) | (0.063) | (0.078) | (0.085) |
| Frailty dummy | 0.435*** | 0.642*** | 0.591*** | 0.316*** | 0.301** | 0.065 |
| | (0.074) | (0.072) | (0.089) | (0.076) | (0.112) | (0.109) |
| Cognition score = 1 (ref. = 0) | 0.421 | -0.244 | -0.046 | 0.062 | 0.197 | -0.296 |
| | (0.388) | (0.400) | (0.428) | (0.423) | (0.596) | (0.570) |

| | | | | | | |
|---|---|---|---|---|---|---|
| Cognition score = 2 (ref. = 0) | -0.361 | -0.629* | -0.382 | -0.459 | -0.024 | -0.364 |
| | (0.262) | (0.245) | (0.271) | (0.239) | (0.378) | (0.294) |
| Cognition score = 3 (ref. = 0) | -0.566* | -0.689** | -0.356 | -0.505* | 0.123 | 0.112 |
| | (0.234) | (0.219) | (0.242) | (0.212) | (0.337) | (0.257) |
| Cognition score = 4 (ref. = 0) | -0.620** | -0.741*** | -0.396 | -0.523* | 0.219 | 0.192 |
| | (0.232) | (0.217) | (0.239) | (0.210) | (0.335) | (0.252) |
| cut1 | -8.253*** | -5.351*** | -1.651 | -1.418 | 0.873 | 1.032 |
| | (0.837) | (0.820) | (0.914) | (0.847) | (1.162) | (1.133) |
| cut2 | -4.666*** | -2.902*** | 1.433 | 1.252 | 2.201 | 2.386* |
| | (0.836) | (0.820) | (0.910) | (0.846) | (1.162) | (1.129) |
| cut3 | -2.072* | -1.571 | 3.420*** | 3.255*** | 3.062** | 4.152*** |
| | (0.834) | (0.819) | (0.910) | (0.846) | (1.162) | (1.126) |
| Country dummies | Yes | Yes | Yes | Yes | Yes | Yes |
| Interview week dummies | Yes | Yes | Yes | Yes | Yes | Yes |
| N | 20,333 | 20,243 | 20,106 | 20,143 | 17,903 | 17,882 |
| Pseudo R2 | 0.089 | 0.119 | 0.081 | 0.073 | 0.362 | 0.024 |

Note: Ordered logit regressions based on the sample that was able to stand up during the chair stand test; the table presents coefficients along with standard errors in parentheses; * p<0.05, ** p<0.01, *** p<0.0

Table A.6: Robustness analysis for overestimating health: ordered logit estimations

| | (1)<br>Less<br>shopping | (2)<br>Less<br>walks | (3)<br>Less<br>meetings | (4)<br>Less<br>visits | (5)<br>Wearing<br>masks | (6)<br>Keeping<br>distance |
|---|---|---|---|---|---|---|
| Overestimating | -0.047 | -0.026 | 0.048 | 0.014 | 0.150 | 0.279 |
| | (0.102) | (0.100) | (0.124) | (0.113) | (0.145) | (0.156) |
| Age | -0.117 | -0.091 | 0.012 | 0.065 | 0.013 | 0.234* |
| | (0.066) | (0.062) | (0.077) | (0.071) | (0.098) | (0.095) |
| Age squared | 0.001** | 0.001* | 0.000 | -0.000 | -0.000 | -0.002* |
| | (0.000) | (0.000) | (0.001) | (0.001) | (0.001) | (0.001) |
| Women | 0.486*** | 0.375*** | 0.328** | 0.189 | 0.463*** | 0.288* |
| | (0.094) | (0.087) | (0.109) | (0.098) | (0.129) | (0.136) |
| Medium education (ref. = low) | -0.258* | -0.025 | 0.020 | -0.064 | -0.120 | 0.166 |
| | (0.109) | (0.107) | (0.133) | (0.121) | (0.162) | (0.166) |
| High education (ref. = low) | -0.313* | -0.292* | -0.172 | -0.377** | 0.053 | -0.276 |
| | (0.135) | (0.129) | (0.156) | (0.146) | (0.181) | (0.193) |
| Chronic disease dummy | 0.018 | 0.008 | 0.180 | -0.072 | 0.205 | 0.066 |
| | (0.122) | (0.120) | (0.145) | (0.138) | (0.173) | (0.180) |
| ADL dummy | 0.369** | 0.239 | 0.268 | 0.249 | 0.205 | 0.389* |
| | (0.126) | (0.124) | (0.162) | (0.150) | (0.180) | (0.192) |
| IADL dummy | 0.438*** | 0.267* | 0.068 | 0.166 | -0.292 | -0.129 |
| | (0.117) | (0.116) | (0.146) | (0.139) | (0.170) | (0.180) |
| Frailty dummy | 0.333** | 0.489*** | 0.436** | 0.201 | 0.067 | -0.213 |
| | (0.111) | (0.108) | (0.139) | (0.126) | (0.159) | (0.166) |
| Cognition score = 1 (ref. = 0) | -0.433 | -0.082 | 0.176 | 0.217 | -0.311 | 0.544 |
| | (0.618) | (0.673) | (1.005) | (0.723) | (0.758) | (1.300) |
| Cognition score = 2 (ref. = 0) | -0.332 | 0.177 | 0.554 | 0.140 | 0.207 | 0.050 |

| | | | | | | |
|---|---|---|---|---|---|---|
| | (0.515) | (0.479) | (0.708) | (0.483) | (0.768) | (0.713) |
| Cognition score = 3 (ref. = 0) | -0.879 | -0.539 | -0.683 | -0.235 | 0.438 | 0.140 |
| | (0.469) | (0.427) | (0.546) | (0.423) | (0.648) | (0.664) |
| Cognition score = 4 (ref. = 0) | -1.149* | -0.674 | -0.928 | -0.264 | 0.478 | 0.581 |
| | (0.461) | (0.420) | (0.535) | (0.414) | (0.633) | (0.648) |
| cut1 | -6.805** | -3.944 | -3.989 | -1.117 | -2.525 | 4.886 |
| | (2.229) | (2.091) | (2.603) | (2.406) | (3.329) | (3.247) |
| cut2 | -3.185 | -1.366 | -0.964 | 1.726 | -1.436 | 5.930 |
| | (2.212) | (2.090) | (2.573) | (2.399) | (3.326) | (3.256) |
| cut3 | -1.023 | -0.152 | 0.756 | 3.474 | -0.700 | 7.208* |
| | (2.209) | (2.088) | (2.570) | (2.396) | (3.321) | (3.250) |
| Country dummies | Yes | Yes | Yes | Yes | Yes | Yes |
| Interview week dummies | Yes | Yes | Yes | Yes | Yes | Yes |
| N | 2,222 | 2,225 | 2,218 | 2,211 | 1,581 | 1,571 |
| Pseudo R2 | 0.153 | 0.130 | 0.097 | 0.093 | 0.315 | 0.047 |

Note: Ordered logit regressions based on the sample that was unable to stand up during the chair stand test; the table presents coefficients along with standard errors in parentheses; * p<0.05, ** p<0.01, *** p<0.0

Table A.7: Robustness analysis for underestimating health: different specification of objective mobility

| | (1) Staying home | (2) Less shopping | (3) Less walks | (4) Less meetings | (5) Less visits | (6) Wearing masks | (7) Keeping distance | (8) Washing hands | (9) Sanitising hands |
|---|---|---|---|---|---|---|---|---|---|
| Underestimating | 0.240*** | 0.170** | 0.208*** | -0.055 | -0.046 | 0.079 | 0.094 | 0.033 | -0.015 |
| | (0.066) | (0.057) | (0.052) | (0.084) | (0.069) | (0.082) | (0.129) | (0.071) | (0.061) |
| Age | -0.024 | -0.045 | -0.113*** | 0.204*** | 0.134*** | 0.115* | 0.244*** | 0.194*** | 0.087** |
| | (0.038) | (0.030) | (0.029) | (0.044) | (0.035) | (0.046) | (0.058) | (0.034) | (0.032) |
| Age squared | 0.001** | 0.001* | 0.001*** | -0.001*** | -0.001** | -0.001* | -0.002*** | -0.002*** | -0.001*** |
| | (0.000) | (0.000) | (0.000) | (0.000) | (0.000) | (0.000) | (0.000) | (0.000) | (0.000) |
| Women | 0.261*** | 0.649*** | 0.270*** | 0.410*** | 0.322*** | 0.417*** | 0.414*** | 0.232*** | 0.122** |
| | (0.045) | (0.032) | (0.031) | (0.049) | (0.040) | (0.045) | (0.075) | (0.044) | (0.037) |
| Medium education (ref. = low) | -0.332*** | -0.040 | -0.209*** | -0.029 | -0.074 | 0.163* | 0.141 | 0.075 | 0.112* |
| | (0.057) | (0.045) | (0.042) | (0.066) | (0.056) | (0.066) | (0.094) | (0.056) | (0.051) |
| High education (ref. = low) | -0.654*** | 0.032 | -0.426*** | 0.192* | 0.017 | 0.364*** | 0.470*** | 0.329*** | 0.190*** |
| | (0.073) | (0.049) | (0.047) | (0.076) | (0.062) | (0.071) | (0.113) | (0.067) | (0.058) |
| Chronic disease dummy | 0.083 | 0.184*** | 0.245*** | 0.082 | 0.097* | 0.237*** | 0.155 | -0.031 | 0.066 |
| | (0.055) | (0.037) | (0.037) | (0.055) | (0.046) | (0.053) | (0.085) | (0.052) | (0.046) |
| ADL dummy | -0.053 | 0.023 | 0.206* | 0.169 | 0.293* | -0.075 | -0.273 | -0.010 | 0.004 |
| | (0.104) | (0.086) | (0.081) | (0.134) | (0.117) | (0.119) | (0.179) | (0.101) | (0.089) |
| IADL dummy | 0.276*** | 0.149* | 0.255*** | 0.012 | 0.062 | -0.039 | -0.146 | -0.256** | -0.047 |
| | (0.082) | (0.074) | (0.068) | (0.111) | (0.092) | (0.098) | (0.158) | (0.083) | (0.076) |
| Frailty dummy | 0.468*** | 0.227* | 0.638*** | 0.643*** | 0.308* | 0.280* | -0.180 | -0.117 | -0.304*** |
| | (0.084) | (0.096) | (0.088) | (0.171) | (0.127) | (0.134) | (0.184) | (0.099) | (0.086) |
| Cognition score = 1 (ref. = 0) | -0.209 | 0.245 | -0.481 | -0.125 | -0.533 | -0.002 | -1.508* | -0.473 | -0.652 |
| | (0.386) | (0.471) | (0.478) | (0.809) | (0.567) | (0.795) | (0.751) | (0.375) | (0.360) |
| Cognition score = 2 (ref. = 0) | -0.767** | -0.146 | -0.616* | -0.415 | -0.261 | -0.154 | -0.713 | 0.237 | -0.120 |

| | | | | | | | | | |
|---|---|---|---|---|---|---|---|---|---|
| | (0.261) | (0.282) | (0.308) | (0.426) | (0.380) | (0.459) | (0.580) | (0.265) | (0.263) |
| Cognition score = 3 (ref. = 0) | -0.954*** | -0.202 | -0.728** | -0.378 | -0.345 | 0.108 | -0.219 | 0.651** | 0.116 |
| | (0.221) | (0.246) | (0.271) | (0.362) | (0.330) | (0.401) | (0.524) | (0.222) | (0.226) |
| Cognition score = 4 (ref. = 0) | -1.064*** | -0.213 | -0.777** | -0.386 | -0.429 | 0.210 | -0.151 | 0.681** | 0.217 |
| | (0.216) | (0.243) | (0.269) | (0.358) | (0.327) | (0.397) | (0.516) | (0.217) | (0.223) |
| Constant | -3.659** | 0.607 | 2.051* | -5.168*** | -3.403** | -1.754 | -4.231* | -4.710*** | -1.063 |
| | (1.321) | (1.008) | (0.994) | (1.448) | (1.185) | (1.542) | (1.993) | (1.158) | (1.073) |
| Country dummies | Yes | Yes | Yes | Yes | Yes | Yes | Yes | Yes | Yes |
| Interview week dummies | Yes | Yes | Yes | Yes | Yes | Yes | Yes | Yes | Yes |
| N | 20,625 | 20,446 | 20,354 | 20,216 | 20,255 | 17,992 | 17,969 | 20,603 | 20,611 |
| Pseudo R2 | 0.184 | 0.093 | 0.212 | 0.080 | 0.061 | 0.524 | 0.046 | 0.036 | 0.069 |

Table A.8: Robustness analysis for overestimating health: different specification of objective mobility

| | (1) Staying home | (2) Less shopping | (3) Less walks | (4) Less meetings | (5) Less visits | (6) Wearing masks | (7) Keeping distance | (8) Washing hands | (9) Sanitising hands |
|---|---|---|---|---|---|---|---|---|---|
| Overestimating | -0.027 | -0.012 | 0.006 | -0.137 | 0.135 | 0.312 | 0.600* | -0.035 | 0.153 |
| | (0.129) | (0.143) | (0.126) | (0.226) | (0.190) | (0.182) | (0.256) | (0.136) | (0.128) |
| Age | 0.039 | -0.048 | -0.082 | 0.136 | 0.264* | 0.043 | 0.337* | 0.084 | 0.092 |
| | (0.088) | (0.099) | (0.083) | (0.135) | (0.110) | (0.132) | (0.144) | (0.086) | (0.079) |
| Age squared | 0.000 | 0.001 | 0.001 | -0.001 | -0.002* | -0.001 | -0.003** | -0.001 | -0.001 |
| | (0.001) | (0.001) | (0.001) | (0.001) | (0.001) | (0.001) | (0.001) | (0.001) | (0.001) |
| Women | 0.498*** | 0.645*** | 0.409*** | 0.207 | 0.187 | 0.539** | 0.499* | 0.330** | 0.167 |
| | (0.124) | (0.126) | (0.111) | (0.204) | (0.168) | (0.168) | (0.229) | (0.126) | (0.119) |
| Medium education (ref. = low) | -0.336* | -0.147 | 0.063 | -0.175 | 0.049 | -0.158 | 0.430 | -0.271 | 0.270* |
| | (0.137) | (0.146) | (0.128) | (0.233) | (0.201) | (0.196) | (0.257) | (0.150) | (0.137) |
| High education (ref. = low) | -0.543** | 0.028 | -0.205 | 0.062 | -0.282 | 0.098 | 0.232 | -0.130 | 0.201 |
| | (0.188) | (0.187) | (0.158) | (0.306) | (0.245) | (0.230) | (0.327) | (0.186) | (0.176) |
| Chronic disease dummy | -0.025 | 0.101 | 0.151 | 0.529* | -0.019 | 0.342 | 0.018 | 0.155 | 0.174 |
| | (0.167) | (0.173) | (0.154) | (0.238) | (0.245) | (0.218) | (0.316) | (0.180) | (0.168) |
| ADL dummy | 0.331* | 0.436* | 0.337* | 0.503 | -0.003 | 0.334 | 0.553 | -0.131 | -0.166 |
| | (0.144) | (0.192) | (0.163) | (0.320) | (0.246) | (0.238) | (0.311) | (0.145) | (0.143) |
| IADL dummy | 0.361** | 0.379* | 0.291* | 0.166 | 0.248 | -0.215 | -0.117 | -0.265 | -0.117 |
| | (0.136) | (0.179) | (0.147) | (0.284) | (0.247) | (0.212) | (0.273) | (0.147) | (0.143) |
| Frailty dummy | 0.200 | 0.282 | 0.518*** | 0.027 | 0.262 | -0.069 | -0.272 | -0.320* | 0.074 |
| | (0.134) | (0.168) | (0.140) | (0.255) | (0.214) | (0.202) | (0.259) | (0.140) | (0.132) |
| Cognition score = 1 (ref. = 0) | -0.311 | 0.742 | 0.224 | -0.115 | -0.705 | -0.563 | -0.928 | -0.092 | 0.366 |
| | (0.593) | (1.227) | (0.879) | (1.148) | (1.296) | (1.137) | (1.361) | (0.635) | (0.618) |
| Cognition score = 2 (ref. = 0) | -0.312 | 0.631 | 0.573 | 0.428 | 0.259 | 0.649 | 0.000 | 0.069 | 0.200 |

| | | | | | | | | | |
|---|---|---|---|---|---|---|---|---|---|
| | (0.469) | (0.743) | (0.645) | (0.740) | (1.035) | (0.999) | (.) | (0.447) | (0.463) |
| Cognition score = 3 (ref. = 0) | -1.146** | 0.185 | -0.381 | 0.057 | -0.598 | 0.544 | 0.537 | 0.412 | 0.149 |
| | (0.399) | (0.577) | (0.510) | (0.260) | (0.782) | (0.810) | (0.749) | (0.383) | (0.395) |
| Cognition score = 4 (ref. = 0) | -1.371*** | -0.197 | -0.474 | 0.000 | -0.395 | 0.582 | 0.989 | 0.660 | 0.419 |
| | (0.386) | (0.556) | (0.498) | (.) | (0.768) | (0.786) | (0.713) | (0.365) | (0.382) |
| Constant | -5.564 | 0.606 | 0.498 | -3.870 | -7.805* | 0.759 | -8.780 | -1.038 | -1.594 |
| | (3.040) | (3.256) | (2.802) | (4.425) | (3.702) | (4.407) | (4.991) | (2.973) | (2.716) |
| Country dummies | Yes | Yes | Yes | Yes | Yes | Yes | Yes | Yes | Yes |
| Interview week dummies | Yes | Yes | Yes | Yes | Yes | Yes | Yes | Yes | Yes |
| N | 2,145 | 2,104 | 2,112 | 2,010 | 2,078 | 1,489 | 1,386 | 2,135 | 2,140 |
| Pseudo R2 | 0.246 | 0.150 | 0.216 | 0.104 | 0.127 | 0.443 | 0.129 | 0.059 | 0.066 |

Table A.9: Robustness analysis for underestimating health: additional control variables

| | (1) Staying home | (2) Less shopping | (3) Less walks | (4) Less meetings | (5) Less visits | (6) Wearing masks | (7) Keeping distance | (8) Washing hands | (9) Sanitising hands |
|---|---|---|---|---|---|---|---|---|---|
| Underestimating | 0.246*** | 0.155** | 0.188*** | -0.050 | -0.059 | 0.069 | 0.094 | 0.042 | -0.008 |
| | (0.067) | (0.058) | (0.053) | (0.086) | (0.070) | (0.083) | (0.131) | (0.072) | (0.062) |
| Age | 0.005 | -0.045 | -0.103** | 0.154*** | 0.090* | 0.085 | 0.211** | 0.200*** | 0.090* |
| | (0.042) | (0.032) | (0.032) | (0.046) | (0.038) | (0.048) | (0.066) | (0.039) | (0.035) |
| Age squared | 0.001 | 0.001* | 0.001*** | -0.001** | -0.001* | -0.001 | -0.002*** | -0.002*** | -0.001*** |
| | (0.000) | (0.000) | (0.000) | (0.000) | (0.000) | (0.000) | (0.000) | (0.000) | (0.000) |
| Women | 0.229*** | 0.653*** | 0.211*** | 0.403*** | 0.329*** | 0.409*** | 0.455*** | 0.250*** | 0.142*** |
| | (0.047) | (0.033) | (0.032) | (0.050) | (0.041) | (0.046) | (0.078) | (0.046) | (0.039) |
| Medium education (ref. = low) | -0.317*** | -0.039 | -0.194*** | -0.034 | -0.071 | 0.161* | 0.115 | 0.067 | 0.112* |
| | (0.058) | (0.045) | (0.042) | (0.067) | (0.057) | (0.067) | (0.095) | (0.057) | (0.051) |
| High education (ref. = low) | -0.649*** | 0.028 | -0.424*** | 0.186* | 0.020 | 0.367*** | 0.431*** | 0.315*** | 0.188** |
| | (0.074) | (0.050) | (0.048) | (0.077) | (0.063) | (0.071) | (0.114) | (0.068) | (0.058) |
| Chronic disease dummy | 0.065 | 0.172*** | 0.216*** | 0.065 | 0.077 | 0.222*** | 0.155 | -0.025 | 0.065 |
| | (0.055) | (0.037) | (0.037) | (0.056) | (0.046) | (0.053) | (0.086) | (0.052) | (0.047) |
| ADL dummy | -0.039 | 0.028 | 0.199* | 0.162 | 0.293* | -0.100 | -0.242 | -0.039 | 0.026 |
| | (0.106) | (0.089) | (0.083) | (0.138) | (0.119) | (0.122) | (0.185) | (0.103) | (0.092) |
| IADL dummy | 0.301*** | 0.134 | 0.244*** | 0.011 | 0.035 | -0.045 | -0.206 | -0.270** | -0.051 |
| | (0.083) | (0.075) | (0.069) | (0.114) | (0.094) | (0.100) | (0.158) | (0.085) | (0.078) |
| Frailty dummy | 0.428*** | 0.208* | 0.593*** | 0.589*** | 0.275* | 0.236 | -0.183 | -0.078 | -0.282** |
| | (0.086) | (0.098) | (0.091) | (0.174) | (0.129) | (0.137) | (0.187) | (0.104) | (0.089) |
| Cognition score = 1 (ref. = 0) | -0.105 | 0.281 | -0.482 | -0.193 | -0.550 | -0.139 | -1.509* | -0.413 | -0.658 |
| | (0.396) | (0.471) | (0.487) | (0.821) | (0.571) | (0.809) | (0.756) | (0.396) | (0.378) |
| Cognition score = 2 (ref. = 0) | -0.663* | -0.065 | -0.566 | -0.372 | -0.112 | -0.126 | -0.645 | 0.251 | -0.172 |

| | | | | | | | | | |
|---|---|---|---|---|---|---|---|---|---|
| | (0.275) | (0.287) | (0.316) | (0.425) | (0.383) | (0.461) | (0.579) | (0.275) | (0.275) |
| Cognition score = 3 (ref. = 0) | -0.824*** | -0.113 | -0.694* | -0.324 | -0.256 | 0.165 | -0.137 | 0.654** | 0.074 |
| | (0.230) | (0.250) | (0.278) | (0.360) | (0.329) | (0.401) | (0.522) | (0.230) | (0.237) |
| Cognition score = 4 (ref. = 0) | -0.917*** | -0.119 | -0.728** | -0.318 | -0.334 | 0.252 | -0.062 | 0.677** | 0.160 |
| | (0.226) | (0.247) | (0.275) | (0.355) | (0.326) | (0.397) | (0.514) | (0.225) | (0.234) |
| Retired | -0.116 | -0.016 | -0.024 | 0.160* | 0.127* | 0.122 | 0.050 | -0.071 | -0.052 |
| | (0.065) | (0.050) | (0.048) | (0.076) | (0.062) | (0.072) | (0.119) | (0.069) | (0.060) |
| Living with partner | 0.065 | 0.352*** | 0.025 | 0.265*** | 0.323*** | 0.178** | 0.303*** | 0.264*** | 0.292*** |
| | (0.055) | (0.039) | (0.039) | (0.057) | (0.047) | (0.056) | (0.086) | (0.051) | (0.046) |
| Feeling depressed or sad | 0.261*** | 0.303*** | 0.474*** | 0.304*** | 0.296*** | 0.292*** | 0.147 | 0.129* | 0.082 |
| | (0.053) | (0.045) | (0.041) | (0.069) | (0.057) | (0.062) | (0.101) | (0.057) | (0.049) |
| Anyone tested positive | -0.064 | 0.119* | -0.070 | 0.132 | 0.106 | 0.001 | 0.266 | 0.188* | 0.060 |
| | (0.090) | (0.059) | (0.059) | (0.096) | (0.076) | (0.081) | (0.156) | (0.088) | (0.074) |
| Constant | -4.905*** | 0.153 | 1.605 | -3.791* | -2.320 | -0.897 | -3.522 | -5.206*** | -1.431 |
| | (1.460) | (1.090) | (1.087) | (1.556) | (1.282) | (1.649) | (2.295) | (1.322) | (1.200) |
| Country dummies | Yes | Yes | Yes | Yes | Yes | Yes | Yes | Yes | Yes |
| Interview week dummies | Yes | Yes | Yes | Yes | Yes | Yes | Yes | Yes | Yes |
| N | 20,280 | 20,108 | 20,020 | 19,882 | 19,921 | 17,720 | 17,705 | 20,265 | 20,273 |
| Pseudo R2 | 0.187 | 0.098 | 0.216 | 0.084 | 0.065 | 0.527 | 0.050 | 0.038 | 0.071 |

Table A.10: Robustness analysis for overestimating health: additional control variables

| | (1) Staying home | (2) Less shopping | (3) Less walks | (4) Less meetings | (5) Less visits | (6) Wearing masks | (7) Keeping distance | (8) Washing hands | (9) Sanitising hands |
|---|---|---|---|---|---|---|---|---|---|
| Overestimating | 0.007 | 0.025 | 0.009 | -0.068 | 0.240 | 0.322 | 0.712** | 0.012 | 0.152 |
| | (0.128) | (0.143) | (0.126) | (0.221) | (0.187) | (0.180) | (0.254) | (0.136) | (0.128) |
| Age | 0.050 | -0.071 | -0.080 | -0.023 | 0.267* | 0.184 | 0.434** | 0.192* | 0.107 |
| | (0.094) | (0.108) | (0.090) | (0.143) | (0.123) | (0.138) | (0.164) | (0.094) | (0.088) |
| Age squared | 0.000 | 0.001 | 0.001 | 0.000 | -0.002* | -0.001 | -0.003** | -0.002* | -0.001 |
| | (0.001) | (0.001) | (0.001) | (0.001) | (0.001) | (0.001) | (0.001) | (0.001) | (0.001) |
| Women | 0.446*** | 0.716*** | 0.380*** | 0.284 | 0.102 | 0.597*** | 0.660** | 0.393** | 0.206 |
| | (0.128) | (0.128) | (0.113) | (0.208) | (0.168) | (0.167) | (0.228) | (0.132) | (0.123) |
| Medium education (ref. = low) | -0.387** | -0.093 | 0.133 | -0.054 | 0.097 | -0.203 | 0.392 | -0.255 | 0.260 |
| | (0.138) | (0.148) | (0.128) | (0.230) | (0.201) | (0.196) | (0.246) | (0.151) | (0.137) |
| High education (ref. = low) | -0.597** | 0.068 | -0.140 | 0.188 | -0.236 | 0.031 | 0.155 | -0.106 | 0.186 |
| | (0.192) | (0.188) | (0.156) | (0.301) | (0.244) | (0.227) | (0.326) | (0.189) | (0.176) |
| Chronic disease dummy | 0.019 | 0.117 | 0.146 | 0.475 | -0.064 | 0.342 | 0.188 | 0.087 | 0.202 |
| | (0.171) | (0.177) | (0.157) | (0.243) | (0.251) | (0.220) | (0.307) | (0.179) | (0.168) |
| ADL dummy | 0.369* | 0.372* | 0.256 | 0.454 | -0.064 | 0.302 | 0.547 | -0.121 | -0.182 |
| | (0.144) | (0.189) | (0.161) | (0.303) | (0.241) | (0.233) | (0.310) | (0.149) | (0.144) |
| IADL dummy | 0.323* | 0.360* | 0.254 | 0.087 | 0.261 | -0.196 | -0.131 | -0.380* | -0.106 |
| | (0.136) | (0.175) | (0.147) | (0.282) | (0.247) | (0.207) | (0.265) | (0.148) | (0.142) |
| Frailty dummy | 0.185 | 0.307 | 0.508*** | 0.107 | 0.271 | -0.082 | -0.046 | -0.258 | 0.059 |
| | (0.134) | (0.168) | (0.142) | (0.255) | (0.213) | (0.203) | (0.262) | (0.140) | (0.133) |
| Cognition score = 1 (ref. = 0) | -0.486 | 0.830 | 0.463 | -0.068 | -0.623 | -0.510 | -1.027 | -0.744 | 0.122 |
| | (0.593) | (1.259) | (0.874) | (1.128) | (1.297) | (1.197) | (1.303) | (0.662) | (0.632) |
| Cognition score = 2 (ref. = 0) | -0.319 | 0.881 | 0.657 | 0.499 | 0.129 | 0.780 | 0.000 | -0.161 | 0.036 |

| | | | | | | | | | |
|---|---|---|---|---|---|---|---|---|---|
| | (0.460) | (0.760) | (0.647) | (0.734) | (0.943) | (0.971) | (.) | (0.489) | (0.478) |
| Cognition score = 3 (ref. = 0) | -1.005* | 0.286 | -0.172 | 0.031 | -0.318 | 0.447 | 0.547 | 0.153 | -0.031 |
| | (0.397) | (0.600) | (0.522) | (0.257) | (0.765) | (0.810) | (0.700) | (0.430) | (0.415) |
| Cognition score = 4 (ref. = 0) | -1.229** | -0.017 | -0.249 | 0.000 | -0.267 | 0.595 | 1.075 | 0.376 | 0.187 |
| | (0.385) | (0.578) | (0.509) | (.) | (0.748) | (0.789) | (0.662) | (0.414) | (0.402) |
| Retired | -0.013 | -0.060 | 0.091 | 0.470 | -0.098 | -0.430 | -0.303 | -0.190 | 0.001 |
| | (0.148) | (0.172) | (0.154) | (0.263) | (0.230) | (0.222) | (0.325) | (0.163) | (0.150) |
| Living with partner | 0.004 | 0.383** | -0.069 | 0.503* | 0.291 | 0.358* | 0.655** | -0.003 | 0.258* |
| | (0.122) | (0.133) | (0.115) | (0.204) | (0.175) | (0.166) | (0.223) | (0.132) | (0.120) |
| Feeling depressed or sad | 0.339** | 0.609*** | 0.472*** | 0.563* | 0.386* | 0.301 | -0.032 | 0.230 | 0.005 |
| | (0.121) | (0.151) | (0.119) | (0.237) | (0.190) | (0.186) | (0.246) | (0.133) | (0.121) |
| Anyone tested positive | -0.368 | 0.006 | -0.355 | -0.108 | -0.006 | 0.230 | 0.835 | 0.272 | 0.417 |
| | (0.258) | (0.236) | (0.199) | (0.394) | (0.331) | (0.343) | (0.721) | (0.270) | (0.248) |
| Constant | -6.197 | 0.535 | 0.178 | 0.720 | -8.404* | -4.566 | -13.044* | -4.539 | -2.049 |
| | (3.222) | (3.570) | (3.010) | (4.766) | (4.154) | (4.655) | (5.690) | (3.207) | (3.000) |
| Country dummies | Yes | Yes | Yes | Yes | Yes | Yes | Yes | Yes | Yes |
| Interview week dummies | Yes | Yes | Yes | Yes | Yes | Yes | Yes | Yes | Yes |
| N | 2,190 | 2,149 | 2,154 | 2,054 | 2,124 | 1,548 | 1,436 | 2,184 | 2,189 |
| Pseudo R2 | 0.238 | 0.165 | 0.223 | 0.111 | 0.129 | 0.447 | 0.142 | 0.067 | 0.075 |

Table A.11: Robustness analysis for underestimating health: additional control variable income

| | (1) Staying home | (2) Less shopping | (3) Less walks | (4) Less meetings | (5) Less visits | (6) Wearing masks | (7) Keeping distance | (8) Washing hands | (9) Sanitising hands |
|---|---|---|---|---|---|---|---|---|---|
| Underestimating | 0.205$^{**}$ | 0.136$^{*}$ | 0.201$^{***}$ | -0.096 | -0.025 | 0.128 | 0.130 | 0.108 | 0.038 |
| | (0.078) | (0.064) | (0.059) | (0.093) | (0.078) | (0.091) | (0.148) | (0.083) | (0.070) |
| Age | -0.045 | -0.071$^{*}$ | -0.103$^{**}$ | 0.201$^{***}$ | 0.136$^{**}$ | 0.117$^{*}$ | 0.211$^{**}$ | 0.215$^{***}$ | 0.098$^{**}$ |
| | (0.046) | (0.035) | (0.035) | (0.051) | (0.041) | (0.053) | (0.068) | (0.041) | (0.037) |
| Age squared | 0.001$^{*}$ | 0.001$^{**}$ | 0.001$^{***}$ | -0.001$^{***}$ | -0.001$^{**}$ | -0.001$^{*}$ | -0.002$^{***}$ | -0.002$^{***}$ | -0.001$^{***}$ |
| | (0.000) | (0.000) | (0.000) | (0.000) | (0.000) | (0.000) | (0.001) | (0.000) | (0.000) |
| Women | 0.255$^{***}$ | 0.691$^{***}$ | 0.262$^{***}$ | 0.412$^{***}$ | 0.329$^{***}$ | 0.405$^{***}$ | 0.500$^{***}$ | 0.251$^{***}$ | 0.146$^{***}$ |
| | (0.053) | (0.036) | (0.036) | (0.056) | (0.044) | (0.050) | (0.087) | (0.051) | (0.043) |
| Medium education (ref. = low) | -0.297$^{***}$ | 0.000 | -0.176$^{***}$ | 0.011 | -0.077 | 0.129 | 0.068 | 0.013 | 0.059 |
| | (0.066) | (0.052) | (0.048) | (0.075) | (0.064) | (0.074) | (0.108) | (0.065) | (0.058) |
| High education (ref. = low) | -0.575$^{***}$ | 0.062 | -0.344$^{***}$ | 0.196$^{*}$ | 0.025 | 0.271$^{***}$ | 0.206 | 0.203$^{**}$ | 0.095 |
| | (0.088) | (0.057) | (0.056) | (0.087) | (0.071) | (0.082) | (0.128) | (0.078) | (0.067) |
| Chronic disease dummy | 0.106 | 0.177$^{***}$ | 0.236$^{***}$ | 0.097 | 0.098 | 0.211$^{***}$ | 0.101 | -0.045 | 0.072 |
| | (0.065) | (0.042) | (0.042) | (0.062) | (0.051) | (0.060) | (0.099) | (0.060) | (0.053) |
| ADL dummy | -0.091 | 0.023 | 0.146 | 0.188 | 0.308$^{*}$ | -0.095 | -0.182 | -0.043 | 0.031 |
| | (0.120) | (0.097) | (0.090) | (0.151) | (0.130) | (0.131) | (0.208) | (0.114) | (0.101) |
| IADL dummy | 0.291$^{**}$ | 0.115 | 0.214$^{**}$ | -0.048 | -0.007 | -0.034 | -0.217 | -0.241$^{**}$ | -0.050 |
| | (0.094) | (0.082) | (0.077) | (0.123) | (0.101) | (0.108) | (0.171) | (0.093) | (0.087) |
| Frailty dummy | 0.528$^{***}$ | 0.206 | 0.643$^{***}$ | 0.586$^{**}$ | 0.214 | 0.297$^{*}$ | -0.125 | -0.180 | -0.357$^{***}$ |
| | (0.097) | (0.109) | (0.100) | (0.191) | (0.139) | (0.149) | (0.212) | (0.113) | (0.098) |
| Cognition score = 1 (ref. = 0) | -0.074 | -0.123 | -0.560 | -0.699 | -0.903 | -0.761 | -2.031$^{*}$ | -0.566 | -0.536 |
| | (0.461) | (0.525) | (0.550) | (0.857) | (0.617) | (0.970) | (0.946) | (0.470) | (0.449) |
| Cognition score = 2 (ref. = 0) | -0.803$^{*}$ | -0.360 | -0.855$^{*}$ | -0.675 | -0.223 | -0.250 | -1.124 | 0.219 | -0.100 |

| | | | | | | | | | |
|---|---|---|---|---|---|---|---|---|---|
| | (0.336) | (0.342) | (0.356) | (0.506) | (0.450) | (0.552) | (0.779) | (0.341) | (0.328) |
| Cognition score = 3 (ref. = 0) | -1.066*** | -0.453 | -0.980** | -0.579 | -0.381 | 0.060 | -0.523 | 0.633* | 0.149 |
| | (0.286) | (0.299) | (0.314) | (0.433) | (0.390) | (0.488) | (0.723) | (0.289) | (0.284) |
| Cognition score = 4 (ref. = 0) | -1.149*** | -0.443 | -0.988** | -0.540 | -0.446 | 0.186 | -0.479 | 0.647* | 0.232 |
| | (0.280) | (0.296) | (0.311) | (0.429) | (0.386) | (0.483) | (0.715) | (0.283) | (0.281) |
| Lowest income | -0.088*** | 0.010 | -0.044*** | -0.002 | -0.000 | 0.063*** | 0.114*** | 0.049*** | 0.063*** |
| | (0.015) | (0.010) | (0.011) | (0.014) | (0.011) | (0.015) | (0.019) | (0.013) | (0.012) |
| Constant | -1.880 | 1.560 | 2.552* | -4.841** | -3.363* | -2.524 | -4.103 | -5.979*** | -2.239 |
| | (1.584) | (1.181) | (1.173) | (1.713) | (1.389) | (1.810) | (2.377) | (1.384) | (1.254) |
| Country dummies | Yes | Yes | Yes | Yes | Yes | Yes | Yes | Yes | Yes |
| Interview week dummies | Yes | Yes | Yes | Yes | Yes | Yes | Yes | Yes | Yes |
| N | 15,768 | 15,652 | 15,577 | 15,500 | 15,513 | 13,877 | 13,864 | 15,755 | 15,762 |
| Pseudo R2 | 0.196 | 0.090 | 0.203 | 0.081 | 0.058 | 0.515 | 0.050 | 0.035 | 0.068 |

Table A.12: Robustness analysis for overestimating health: additional control variable income

| | (1) Staying home | (2) Less shopping | (3) Less walks | (4) Less meetings | (5) Less visits | (6) Wearing masks | (7) Keeping distance | (8) Washing hands | (9) Sanitising hands |
|---|---|---|---|---|---|---|---|---|---|
| Overestimating | 0.104 | 0.044 | -0.091 | -0.108 | 0.274 | 0.172 | 0.679* | -0.027 | 0.058 |
| | (0.151) | (0.159) | (0.142) | (0.246) | (0.209) | (0.198) | (0.282) | (0.155) | (0.143) |
| Age | 0.150 | -0.043 | -0.045 | 0.059 | 0.274* | -0.035 | 0.372* | 0.078 | 0.058 |
| | (0.111) | (0.115) | (0.095) | (0.164) | (0.123) | (0.141) | (0.164) | (0.104) | (0.095) |
| Age squared | -0.000 | 0.001 | 0.001 | -0.000 | -0.002* | 0.000 | -0.003* | -0.001 | -0.001 |
| | (0.001) | (0.001) | (0.001) | (0.001) | (0.001) | (0.001) | (0.001) | (0.001) | (0.001) |
| Women | 0.463** | 0.707*** | 0.496*** | 0.358 | 0.217 | 0.526** | 0.596* | 0.369* | 0.176 |
| | (0.141) | (0.139) | (0.126) | (0.219) | (0.185) | (0.189) | (0.249) | (0.144) | (0.135) |
| Medium education (ref. = low) | -0.344* | -0.044 | 0.045 | -0.016 | -0.061 | -0.074 | 0.455 | -0.362* | 0.121 |
| | (0.163) | (0.164) | (0.144) | (0.260) | (0.227) | (0.215) | (0.279) | (0.173) | (0.156) |
| High education (ref. = low) | -0.523* | 0.064 | 0.042 | 0.281 | -0.381 | 0.062 | 0.082 | -0.122 | 0.052 |
| | (0.223) | (0.214) | (0.182) | (0.355) | (0.281) | (0.264) | (0.382) | (0.223) | (0.209) |
| Chronic disease dummy | -0.157 | 0.289 | 0.113 | 0.573* | 0.032 | 0.355 | -0.093 | 0.223 | 0.139 |
| | (0.193) | (0.192) | (0.179) | (0.279) | (0.284) | (0.243) | (0.351) | (0.199) | (0.188) |
| ADL dummy | 0.491** | 0.433* | 0.415* | 0.472 | 0.045 | 0.220 | 0.270 | -0.148 | -0.167 |
| | (0.165) | (0.208) | (0.181) | (0.343) | (0.271) | (0.259) | (0.335) | (0.167) | (0.165) |
| IADL dummy | 0.437** | 0.367 | 0.282 | 0.217 | 0.170 | -0.161 | 0.006 | -0.276 | -0.196 |
| | (0.163) | (0.198) | (0.166) | (0.315) | (0.287) | (0.242) | (0.307) | (0.174) | (0.166) |
| Frailty dummy | 0.407** | 0.323 | 0.473** | 0.013 | 0.264 | 0.004 | -0.164 | -0.297 | 0.068 |
| | (0.154) | (0.185) | (0.157) | (0.286) | (0.239) | (0.222) | (0.286) | (0.161) | (0.153) |
| Cognition score = 1 (ref. = 0) | -0.300 | 0.000 | 0.000 | 0.000 | 0.000 | 0.000 | 0.000 | -0.746 | 0.444 |
| | (0.826) | (.) | (.) | (.) | (.) | (.) | (.) | (0.862) | (0.850) |
| Cognition score = 2 (ref. = 0) | -0.313 | 1.016 | 0.720 | 0.981 | 0.151 | -0.194 | 0.000 | -0.133 | 0.494 |

| | | | | | | | | | |
|---|---|---|---|---|---|---|---|---|---|
| | (0.618) | (0.842) | (0.729) | (0.990) | (0.963) | (1.129) | (.) | (0.589) | (0.585) |
| Cognition score = 3 (ref. = 0) | -1.283* | 0.289 | -0.087 | -0.178 | -0.354 | 0.172 | 0.383 | 0.242 | 0.490 |
| | (0.530) | (0.650) | (0.595) | (0.278) | (0.777) | (0.915) | (0.958) | (0.516) | (0.499) |
| Cognition score = 4 (ref. = 0) | -1.459** | -0.024 | -0.232 | 0.000 | -0.114 | 0.307 | 0.799 | 0.503 | 0.633 |
| | (0.515) | (0.627) | (0.580) | (.) | (0.757) | (0.890) | (0.917) | (0.497) | (0.483) |
| Lowest income | -0.075 | 0.023 | -0.055 | 0.079 | 0.094 | 0.037 | 0.079 | 0.055 | 0.115** |
| | (0.045) | (0.044) | (0.036) | (0.049) | (0.058) | (0.054) | (0.082) | (0.040) | (0.040) |
| Constant | -8.515* | -0.260 | -0.299 | -3.094 | -9.559* | 2.968 | -10.529 | -1.459 | -1.758 |
| | (3.915) | (3.809) | (3.219) | (5.279) | (4.233) | (4.765) | (5.665) | (3.592) | (3.274) |
| Country dummies | Yes | Yes | Yes | Yes | Yes | Yes | Yes | Yes | Yes |
| Interview week dummies | Yes | Yes | Yes | Yes | Yes | Yes | Yes | Yes | Yes |
| N | 1,658 | 1,620 | 1,624 | 1,569 | 1,596 | 1,129 | 1,106 | 1,655 | 1,655 |
| Pseudo R2 | 0.270 | 0.151 | 0.210 | 0.123 | 0.122 | 0.396 | 0.131 | 0.063 | 0.065 |

Table A.13: Robustness analysis for underestimating health: interaction between country and interview week dummies

| | (1) Staying home | (2) Less shopping | (3) Less walks | (4) Less meetings | (5) Less visits | (6) Wearing masks | (7) Keeping distance | (8) Washing hands | (9) Sanitising hands |
|---|---|---|---|---|---|---|---|---|---|
| Underestimating | 0.250*** | 0.164** | 0.192*** | -0.058 | -0.043 | 0.070 | 0.114 | 0.040 | -0.018 |
| | (0.066) | (0.057) | (0.052) | (0.085) | (0.070) | (0.082) | (0.132) | (0.072) | (0.061) |
| Age | -0.031 | -0.044 | -0.115*** | 0.210*** | 0.137*** | 0.109* | 0.252*** | 0.196*** | 0.087** |
| | (0.039) | (0.031) | (0.030) | (0.044) | (0.036) | (0.046) | (0.059) | (0.035) | (0.032) |
| Age squared | 0.001** | 0.001* | 0.001*** | -0.001*** | -0.001*** | -0.001* | -0.002*** | -0.002*** | -0.001*** |
| | (0.000) | (0.000) | (0.000) | (0.000) | (0.000) | (0.000) | (0.000) | (0.000) | (0.000) |
| Women | 0.263*** | 0.646*** | 0.272*** | 0.411*** | 0.329*** | 0.419*** | 0.418*** | 0.228*** | 0.120** |
| | (0.045) | (0.032) | (0.032) | (0.050) | (0.040) | (0.046) | (0.076) | (0.044) | (0.038) |
| Medium education (ref. = low) | -0.323*** | -0.039 | -0.214*** | -0.041 | -0.073 | 0.172** | 0.119 | 0.089 | 0.119* |
| | (0.057) | (0.045) | (0.042) | (0.067) | (0.057) | (0.066) | (0.095) | (0.057) | (0.051) |
| High education (ref. = low) | -0.638*** | 0.030 | -0.427*** | 0.190* | 0.018 | 0.370*** | 0.466*** | 0.341*** | 0.193*** |
| | (0.074) | (0.049) | (0.048) | (0.076) | (0.063) | (0.071) | (0.114) | (0.067) | (0.058) |
| Chronic disease dummy | 0.090 | 0.182*** | 0.241*** | 0.078 | 0.094* | 0.245*** | 0.146 | -0.032 | 0.068 |
| | (0.055) | (0.037) | (0.037) | (0.055) | (0.046) | (0.053) | (0.086) | (0.052) | (0.046) |
| ADL dummy | -0.074 | 0.029 | 0.214** | 0.188 | 0.311** | -0.069 | -0.284 | -0.012 | 0.016 |
| | (0.105) | (0.087) | (0.081) | (0.137) | (0.118) | (0.120) | (0.182) | (0.102) | (0.090) |
| IADL dummy | 0.288*** | 0.136 | 0.246*** | 0.010 | 0.058 | -0.057 | -0.127 | -0.238** | -0.055 |
| | (0.083) | (0.074) | (0.069) | (0.113) | (0.092) | (0.099) | (0.160) | (0.084) | (0.077) |
| Frailty dummy | 0.489*** | 0.234* | 0.663*** | 0.653*** | 0.309* | 0.283* | -0.230 | -0.124 | -0.310*** |
| | (0.086) | (0.097) | (0.089) | (0.173) | (0.128) | (0.136) | (0.185) | (0.101) | (0.087) |
| Cognition score = 1 (ref. = 0) | -0.132 | 0.308 | -0.485 | -0.146 | -0.513 | 0.076 | -1.367 | -0.418 | -0.647 |
| | (0.391) | (0.468) | (0.478) | (0.805) | (0.572) | (0.807) | (0.743) | (0.381) | (0.363) |
| Cognition score = 2 (ref. = 0) | -0.761** | -0.162 | -0.621* | -0.469 | -0.232 | -0.133 | -0.633 | 0.210 | -0.145 |
| | (0.271) | (0.284) | (0.311) | (0.421) | (0.384) | (0.467) | (0.579) | (0.271) | (0.268) |

| | | | | | | | | | |
|---|---|---|---|---|---|---|---|---|---|
| Cognition score = 3 (ref. = 0) | -0.916*** | -0.178 | -0.717** | -0.374 | -0.318 | 0.176 | -0.076 | 0.650** | 0.136 |
| | (0.226) | (0.247) | (0.274) | (0.355) | (0.332) | (0.409) | (0.521) | (0.227) | (0.229) |
| Cognition score = 4 (ref. = 0) | -1.021*** | -0.191 | -0.761** | -0.390 | -0.404 | 0.258 | -0.036 | 0.676** | 0.237 |
| | (0.221) | (0.244) | (0.272) | (0.351) | (0.329) | (0.404) | (0.512) | (0.222) | (0.226) |
| Constant | -3.351* | 0.480 | 2.166* | -5.375*** | -3.559** | -1.706 | -4.643* | -4.750*** | -1.067 |
| | (1.340) | (1.015) | (1.005) | (1.463) | (1.201) | (1.560) | (2.032) | (1.182) | (1.093) |
| Country dummies | Yes | Yes | Yes | Yes | Yes | Yes | Yes | Yes | Yes |
| Interview week dummies | Yes | Yes | Yes | Yes | Yes | Yes | Yes | Yes | Yes |
| Interaction between dummies | Yes | Yes | Yes | Yes | Yes | Yes | Yes | Yes | Yes |
| N | 20,276 | 20,299 | 20,217 | 19,903 | 20,021 | 17,234 | 17,293 | 20,458 | 20,427 |
| Pseudo R2 | 0.190 | 0.097 | 0.214 | 0.087 | 0.065 | 0.513 | 0.056 | 0.041 | 0.074 |

Table A.14: Robustness analysis for overestimating health: interaction between country and interview week dummies

| | (1) Staying home | (2) Less shopping | (3) Less walks | (4) Less meetings | (5) Less visits | (6) Wearing masks | (7) Keeping distance | (8) Washing hands | (9) Sanitising hands |
|---|---|---|---|---|---|---|---|---|---|
| Overestimating | -0.012 | -0.024 | 0.011 | -0.148 | 0.181 | 0.337 | 0.701** | -0.007 | 0.115 |
| | (0.130) | (0.145) | (0.129) | (0.225) | (0.186) | (0.186) | (0.264) | (0.137) | (0.128) |
| Age | 0.030 | -0.051 | -0.087 | 0.114 | 0.232* | 0.083 | 0.435** | 0.136 | 0.110 |
| | (0.088) | (0.102) | (0.084) | (0.131) | (0.111) | (0.137) | (0.154) | (0.089) | (0.083) |
| Age squared | 0.000 | 0.001 | 0.001 | -0.001 | -0.001 | -0.001 | -0.003** | -0.001* | -0.001 |
| | (0.001) | (0.001) | (0.001) | (0.001) | (0.001) | (0.001) | (0.001) | (0.001) | (0.001) |
| Women | 0.498*** | 0.752*** | 0.421*** | 0.299 | 0.177 | 0.618*** | 0.629** | 0.381** | 0.195 |
| | (0.124) | (0.127) | (0.114) | (0.202) | (0.171) | (0.177) | (0.234) | (0.127) | (0.119) |
| Medium education (ref. = low) | -0.380** | -0.049 | 0.090 | -0.080 | 0.066 | -0.185 | 0.503* | -0.336* | 0.223 |
| | (0.139) | (0.149) | (0.130) | (0.229) | (0.205) | (0.202) | (0.256) | (0.154) | (0.138) |
| High education (ref. = low) | -0.603** | 0.144 | -0.161 | 0.192 | -0.218 | 0.047 | 0.313 | -0.186 | 0.198 |
| | (0.188) | (0.190) | (0.161) | (0.305) | (0.245) | (0.235) | (0.321) | (0.193) | (0.177) |
| Chronic disease dummy | 0.035 | 0.202 | 0.236 | 0.612* | 0.060 | 0.331 | 0.115 | 0.117 | 0.132 |
| | (0.171) | (0.181) | (0.159) | (0.247) | (0.243) | (0.223) | (0.305) | (0.185) | (0.169) |
| ADL dummy | 0.363* | 0.413* | 0.315 | 0.476 | -0.005 | 0.375 | 0.583 | -0.086 | -0.178 |
| | (0.144) | (0.194) | (0.164) | (0.311) | (0.244) | (0.239) | (0.317) | (0.146) | (0.147) |
| IADL dummy | 0.310* | 0.479** | 0.317* | 0.138 | 0.318 | -0.160 | 0.032 | -0.314* | -0.104 |
| | (0.139) | (0.182) | (0.148) | (0.290) | (0.251) | (0.208) | (0.282) | (0.152) | (0.144) |
| Frailty dummy | 0.250 | 0.269 | 0.496*** | 0.050 | 0.273 | -0.074 | -0.248 | -0.279* | 0.037 |
| | (0.134) | (0.171) | (0.144) | (0.254) | (0.217) | (0.203) | (0.278) | (0.140) | (0.132) |
| Cognition score = 1 (ref. = 0) | -0.417 | 0.519 | 0.269 | 0.142 | -0.637 | -0.222 | -1.706 | -0.321 | 0.071 |
| | (0.615) | (1.268) | (0.864) | (1.215) | (1.378) | (1.107) | (1.710) | (0.674) | (0.651) |
| Cognition score = 2 (ref. = 0) | -0.337 | 0.810 | 0.493 | 0.492 | -0.039 | 0.842 | 0.000 | 0.100 | 0.047 |
| | (0.468) | (0.763) | (0.669) | (0.750) | (0.975) | (1.040) | (.) | (0.457) | (0.489) |

| | | | | | | | | | |
|---|---|---|---|---|---|---|---|---|---|
| Cognition score = 3 (ref. = 0) | -1.214** | 0.126 | -0.355 | 0.068 | -0.558 | 0.582 | 0.536 | 0.397 | 0.018 |
| | (0.405) | (0.633) | (0.540) | (0.259) | (0.815) | (0.851) | (0.755) | (0.388) | (0.421) |
| Cognition score = 4 (ref. = 0) | -1.460*** | -0.280 | -0.465 | 0.000 | -0.527 | 0.796 | 1.184 | 0.698 | 0.318 |
| | (0.393) | (0.617) | (0.530) | (.) | (0.802) | (0.835) | (0.726) | (0.371) | (0.409) |
| Constant | -4.867 | 0.437 | 0.953 | -3.679 | -7.103 | -1.133 | -12.808* | -2.175 | -1.812 |
| | (3.065) | (3.360) | (2.833) | (4.283) | (3.753) | (4.563) | (5.263) | (3.089) | (2.849) |
| Country dummies | Yes | Yes | Yes | Yes | Yes | Yes | Yes | Yes | Yes |
| Interview week dummies | Yes | Yes | Yes | Yes | Yes | Yes | Yes | Yes | Yes |
| Interaction between dummies | Yes | Yes | Yes | Yes | Yes | Yes | Yes | Yes | Yes |
| N | 2,186 | 2,086 | 2,141 | 1,628 | 1,849 | 1,310 | 1,081 | 2,143 | 2,149 |
| Pseudo R2 | 0.255 | 0.172 | 0.234 | 0.102 | 0.133 | 0.370 | 0.143 | 0.086 | 0.084 |

Table A.15: Robustness analysis for underestimating health: controlling for public health measures at the time of the interview

| | (1) Staying home | (2) Less shopping | (3) Less walks | (4) Less meetings | (5) Less visits | (6) Wearing masks | (7) Keeping distance | (8) Washing hands | (9) Sanitising hands |
|---|---|---|---|---|---|---|---|---|---|
| Underestimating | 0.263*** | 0.200*** | 0.213*** | -0.063 | -0.048 | 0.069 | 0.088 | 0.047 | -0.033 |
| | (0.066) | (0.059) | (0.053) | (0.088) | (0.071) | (0.082) | (0.133) | (0.073) | (0.062) |
| Age | -0.022 | -0.055 | -0.104*** | 0.189*** | 0.132*** | 0.123** | 0.196** | 0.191*** | 0.075* |
| | (0.039) | (0.031) | (0.030) | (0.047) | (0.037) | (0.046) | (0.061) | (0.036) | (0.033) |
| Age squared | 0.001* | 0.001* | 0.001*** | -0.001*** | -0.001** | -0.001** | -0.002*** | -0.002*** | -0.001*** |
| | (0.000) | (0.000) | (0.000) | (0.000) | (0.000) | (0.000) | (0.000) | (0.000) | (0.000) |
| Women | 0.271*** | 0.638*** | 0.277*** | 0.425*** | 0.309*** | 0.415*** | 0.396*** | 0.231*** | 0.108** |
| | (0.046) | (0.033) | (0.032) | (0.052) | (0.041) | (0.046) | (0.078) | (0.045) | (0.039) |
| Medium education (ref. = low) | -0.330*** | -0.033 | -0.202*** | -0.026 | -0.074 | 0.179** | 0.151 | 0.095 | 0.105* |
| | (0.057) | (0.046) | (0.043) | (0.070) | (0.059) | (0.066) | (0.097) | (0.058) | (0.053) |
| High education (ref. = low) | -0.653*** | 0.035 | -0.424*** | 0.165* | -0.001 | 0.382*** | 0.466*** | 0.355*** | 0.146* |
| | (0.074) | (0.051) | (0.048) | (0.081) | (0.065) | (0.071) | (0.118) | (0.069) | (0.060) |
| Chronic disease dummy | 0.081 | 0.167*** | 0.240*** | 0.070 | 0.097* | 0.239*** | 0.159 | -0.043 | 0.057 |
| | (0.055) | (0.038) | (0.038) | (0.058) | (0.048) | (0.053) | (0.088) | (0.053) | (0.048) |
| ADL dummy | -0.080 | -0.002 | 0.212* | 0.155 | 0.301* | -0.075 | -0.266 | -0.052 | -0.005 |
| | (0.105) | (0.089) | (0.083) | (0.141) | (0.121) | (0.120) | (0.184) | (0.103) | (0.091) |
| IADL dummy | 0.300*** | 0.144 | 0.238*** | 0.006 | 0.036 | -0.040 | -0.138 | -0.235** | -0.069 |
| | (0.083) | (0.075) | (0.070) | (0.116) | (0.094) | (0.100) | (0.163) | (0.085) | (0.079) |
| Frailty dummy | 0.465*** | 0.225* | 0.641*** | 0.776*** | 0.311* | 0.297* | -0.185 | -0.090 | -0.280** |
| | (0.085) | (0.098) | (0.090) | (0.186) | (0.130) | (0.136) | (0.188) | (0.102) | (0.088) |
| Cognition score = 1 (ref. = 0) | -0.124 | 0.279 | -0.537 | 0.572 | -0.509 | -0.002 | -1.222 | -0.322 | -0.617 |
| | (0.391) | (0.472) | (0.473) | (1.095) | (0.568) | (0.802) | (0.810) | (0.387) | (0.367) |
| Cognition score = 2 (ref. = 0) | -0.767** | -0.150 | -0.575 | -0.409 | -0.121 | -0.187 | -0.560 | 0.279 | -0.061 |
| | (0.271) | (0.285) | (0.312) | (0.432) | (0.388) | (0.463) | (0.593) | (0.274) | (0.270) |

| | | | | | | | | | |
|---|---|---|---|---|---|---|---|---|---|
| Cognition score = 3 (ref. = 0) | -0.939*** | -0.157 | -0.708** | -0.338 | -0.311 | 0.104 | -0.137 | 0.676** | 0.146 |
| | (0.226) | (0.246) | (0.272) | (0.362) | (0.330) | (0.404) | (0.525) | (0.226) | (0.229) |
| Cognition score = 4 (ref. = 0) | -1.041*** | -0.173 | -0.755** | -0.344 | -0.378 | 0.206 | -0.123 | 0.695** | 0.251 |
| | (0.221) | (0.243) | (0.270) | (0.357) | (0.327) | (0.399) | (0.517) | (0.221) | (0.226) |
| Mandatory mask use in all public spaces | -0.105 | -0.109 | -0.159 | 0.071 | -0.142 | 0.037 | 0.064 | -0.040 | -0.048 |
| | (0.106) | (0.082) | (0.085) | (0.142) | (0.109) | (0.151) | (0.203) | (0.114) | (0.101) |
| Restrictions, private gatherings | 0.057 | -0.105 | 0.058 | 0.015 | 0.046 | 0.047 | 0.239 | 0.015 | -0.166 |
| | (0.139) | (0.088) | (0.087) | (0.163) | (0.119) | (0.122) | (0.251) | (0.124) | (0.110) |
| Constant | -3.756** | 1.018 | 1.762 | -4.633** | -3.330** | -2.010 | -2.649 | -4.647*** | -0.659 |
| | (1.352) | (1.039) | (1.021) | (1.549) | (1.239) | (1.548) | (2.099) | (1.200) | (1.112) |
| Country dummies | Yes | Yes | Yes | Yes | Yes | Yes | Yes | Yes | Yes |
| Interview week dummies | Yes | Yes | Yes | Yes | Yes | Yes | Yes | Yes | Yes |
| N | 19,437 | 19,263 | 19,173 | 19,016 | 19,056 | 16,848 | 16,827 | 19,416 | 19,423 |
| Pseudo R2 | 0.179 | 0.094 | 0.201 | 0.078 | 0.061 | 0.486 | 0.045 | 0.035 | 0.069 |

Table A.16: Robustness analysis for overestimating health: controlling for public health measures at the time of the interview

| | (1)<br>Staying home | (2)<br>Less shopping | (3)<br>Less walks | (4)<br>Less meetings | (5)<br>Less visits | (6)<br>Wearing masks | (7)<br>Keeping distance | (8)<br>Washing hands | (9)<br>Sanitising hands |
|---|---|---|---|---|---|---|---|---|---|
| Overestimating | -0.004 | -0.043 | 0.040 | -0.229 | 0.132 | 0.245 | 0.770** | -0.037 | 0.151 |
| | (0.127) | (0.144) | (0.126) | (0.225) | (0.188) | (0.178) | (0.263) | (0.135) | (0.128) |
| Age | 0.010 | -0.077 | -0.085 | 0.073 | 0.238* | 0.057 | 0.422** | 0.140 | 0.110 |
| | (0.086) | (0.101) | (0.084) | (0.142) | (0.111) | (0.131) | (0.143) | (0.087) | (0.080) |
| Age squared | 0.000 | 0.001 | 0.001 | -0.000 | -0.002 | -0.001 | -0.003** | -0.001* | -0.001* |
| | (0.001) | (0.001) | (0.001) | (0.001) | (0.001) | (0.001) | (0.001) | (0.001) | (0.001) |
| Women | 0.487*** | 0.695*** | 0.416*** | 0.350 | 0.189 | 0.585*** | 0.521* | 0.419*** | 0.222 |
| | (0.122) | (0.126) | (0.111) | (0.199) | (0.170) | (0.166) | (0.233) | (0.125) | (0.118) |
| Medium education (ref. = low) | -0.395** | -0.088 | 0.064 | -0.107 | 0.127 | -0.209 | 0.391 | -0.271 | 0.202 |
| | (0.135) | (0.149) | (0.127) | (0.233) | (0.199) | (0.194) | (0.249) | (0.149) | (0.136) |
| High education (ref. = low) | -0.618*** | 0.071 | -0.172 | 0.115 | -0.024 | 0.059 | 0.333 | -0.086 | 0.125 |
| | (0.187) | (0.191) | (0.158) | (0.302) | (0.251) | (0.226) | (0.348) | (0.191) | (0.176) |
| Chronic disease dummy | -0.010 | 0.170 | 0.173 | 0.653** | -0.005 | 0.320 | 0.112 | 0.202 | 0.158 |
| | (0.166) | (0.173) | (0.155) | (0.238) | (0.245) | (0.215) | (0.304) | (0.177) | (0.169) |
| ADL dummy | 0.323* | 0.379* | 0.249 | 0.416 | 0.019 | 0.282 | 0.745* | -0.072 | -0.181 |
| | (0.142) | (0.190) | (0.160) | (0.310) | (0.240) | (0.227) | (0.319) | (0.144) | (0.142) |
| IADL dummy | 0.322* | 0.383* | 0.371* | 0.028 | 0.093 | -0.169 | -0.103 | -0.328* | -0.102 |
| | (0.136) | (0.179) | (0.148) | (0.285) | (0.247) | (0.202) | (0.281) | (0.147) | (0.142) |
| Frailty dummy | 0.238 | 0.267 | 0.487*** | 0.011 | 0.373 | -0.135 | -0.079 | -0.311* | 0.037 |
| | (0.132) | (0.169) | (0.140) | (0.255) | (0.216) | (0.197) | (0.264) | (0.140) | (0.131) |
| Cognition score = 1 (ref. = 0) | -0.474 | 0.668 | 0.133 | 0.077 | -0.501 | -0.375 | -0.949 | -0.359 | 0.286 |
| | (0.589) | (1.205) | (0.856) | (1.160) | (1.297) | (1.122) | (1.381) | (0.629) | (0.616) |
| Cognition score = 2 (ref. = 0) | -0.418 | 0.907 | 0.504 | 0.529 | 0.509 | 0.877 | 0.000 | 0.049 | 0.114 |
| | (0.448) | (0.777) | (0.641) | (0.730) | (1.027) | (0.996) | (.) | (0.448) | (0.454) |

| | | | | | | | | | |
|---|---|---|---|---|---|---|---|---|---|
| Cognition score = 3 (ref. = 0) | -1.277*** | 0.024 | -0.465 | 0.088 | -0.530 | 0.495 | 0.498 | 0.314 | 0.089 |
| | (0.387) | (0.571) | (0.506) | (0.258) | (0.753) | (0.814) | (0.743) | (0.385) | (0.392) |
| Cognition score = 4 (ref. = 0) | -1.459*** | -0.264 | -0.499 | 0.000 | -0.414 | 0.597 | 1.075 | 0.597 | 0.348 |
| | (0.375) | (0.551) | (0.495) | (.) | (0.737) | (0.792) | (0.708) | (0.369) | (0.379) |
| Mandatory mask use in all public spaces | -0.433 | -0.569* | -0.476 | 0.439 | 0.689 | -0.134 | 0.872 | -0.257 | -0.446 |
| | (0.265) | (0.261) | (0.264) | (0.707) | (0.468) | (0.534) | (0.561) | (0.276) | (0.274) |
| Restrictions, private gatherings | -0.449 | -0.477 | -0.226 | 0.314 | -0.582 | -0.007 | 0.695 | 0.044 | 0.222 |
| | (0.359) | (0.332) | (0.303) | (0.688) | (0.555) | (0.487) | (0.772) | (0.352) | (0.341) |
| Constant | -4.463 | 1.590 | 0.689 | -2.214 | -7.081 | 0.283 | -11.971* | -2.915 | -1.995 |
| | (2.994) | (3.316) | (2.815) | (4.604) | (3.724) | (4.373) | (4.940) | (2.974) | (2.735) |
| Country dummies | Yes | Yes | Yes | Yes | Yes | Yes | Yes | Yes | Yes |
| Interview week dummies | Yes | Yes | Yes | Yes | Yes | Yes | Yes | Yes | Yes |
| N | 2,182 | 2,141 | 2,144 | 2,040 | 2,118 | 1,505 | 1,393 | 2,172 | 2,175 |
| Pseudo R2 | 0.236 | 0.152 | 0.208 | 0.106 | 0.131 | 0.400 | 0.137 | 0.065 | 0.069 |

## Supplementary Material

ckab221_Supplementary_Data

