# Older Europeans’ health perception and their adaptive behaviour during the COVID-19 pandemic

**DOI:** 10.1093/eurpub/ckab221

**Published:** 2022-01-03

**Authors:** Sonja Spitzer, Mujaheed Shaikh, Daniela Weber

**Affiliations:** 1 Department of Demography, University of Vienna, Wittgenstein Centre for Demography and Global Human Capital (IIASA, OeAW, Univ. Vienna), 1030 Vienna, Austria; 2 Political Economy Cluster, Hertie School, Berlin, Germany; 3 Health Economics and Policy Division, Vienna University of Economics and Business, Vienna, Austria; 4 Population and Just Societies Program, International Institute for Applied Systems Analysis (IIASA), Wittgenstein Centre for Demography and Global Human Capital (IIASA, OeAW, Univ. Vienna), Laxenburg, Austria

## Abstract

**Background:**

Although older adults are more vulnerable to the COVID-19 virus, a significant proportion of them do not follow recommended guidelines concerning preventive actions during the ongoing pandemic. This article analyses the role of biased health beliefs for adaptive health behaviour such as reduced mobility, protection in public spaces and hygiene measures, for the population aged 50 and older in 13 European countries.

**Methods:**

Health perception is measured based on the difference between self-reported health and physical performance tests for over 24 000 individuals included in the most recent Survey of Health, Ageing and Retirement in Europe. Logistic regressions are employed to explore how over- and underestimating health are related to preventive behaviours.

**Results:**

Results suggest that older adults who underestimate their health are more likely to show adaptive behaviour related to mobility reductions. In particular, they are more likely to stay at home, shop less and go for walks less often. In contrast, overestimating health is not significantly associated with reduced mobility. Protective behaviour in public spaces and adopting hygiene measures do not vary systematically between health perception groups.

**Conclusion:**

As health beliefs appear relevant for the adoption of preventive health behaviours related to mobility, they have serious consequences for the health and well-being of older Europeans. Although adaptive behaviour helps to contain the virus, exaggerated mobility reduction in those who underestimate their health might be contributing to the already high social isolation and loneliness of older adults during the ongoing pandemic.

## Introduction

Older adults are disproportionately affected by the SARS-CoV-2 (COVID-19) pandemic and have significantly higher rates of hospitalization and mortality.^1–4^ To prevent further spread of COVID-19 and to combat the related health, economic and social crises, governments across the world have recommended mitigation behaviours such as social distancing, mask-wearing and frequent hand-washing.

Recent evidence from surveys, however, shows that a significant proportion of older adults, despite their higher vulnerability, do not follow the recommended guidelines. The Centers for Disease Control and Prevention of the USA report that as of April 2020, 16% of adults over the age of 60 did not wear a face mask, 25% did not cancel or postpone social activities and about 14% did not avoid crowded or public places.^5^^,^^6^ observed shoppers in retail stores in the USA and found that only 57% of older adults wore masks. Similar reports emerged during prior pandemic outbreaks such as the Swine Flu, where 32% of older adults in England, Scotland and Wales reported not following recommended strategies.^7^

In addition to age-related deviation from protective guidelines during COVID-19, recent evidence has also explored the role of individual demographic characteristics such as gender, socioeconomic factors like education and income and psychological differences such as anxiety and optimism.^8^ We extend this nascent but highly topical literature by investigating the role of biased health perception in explaining the (non)uptake of preventive actions among Europeans aged at least 50 years. Biased health perception that is, over- or underestimating one’s own health, was shown to increase substantially with age^9^; it is further relevant for the adoption of risky health behaviours and has serious consequences for health and well-being.^10^^,^^11^ In addition, incorrect beliefs about one’s own health can affect one’s perception of susceptibility to a disease and how severe that disease will be—which are important elements of preventive action according to the Health Belief Model.^12^ We hypothesize that, relative to people whose perception is accurate, those who underestimate their health show more adaptive behaviour to preventive action and those who overestimate their health show less. The relationship could operate through increased (decreased) perception of susceptibility and severity for individuals who underestimate (overestimate).

## Methods

### Data and sample

Our analyses are based on the Survey of Health, Ageing and Retirement in Europe (SHARE) COVID-19 survey, which targets Europeans aged 50 and older.^13^^,^^14^ The survey was conducted between May and August 2020 and provides rich information on health and health behaviour during the pandemic (98.3% of all interviews were conducted in June and July 2020). It constitutes the eighth survey wave of the SHARE panel and can be matched with the previous seven waves, which cover more general information on health, socio-economic status, physical performance tests and thus health perception. We match information on health behaviour from the COVID-19 survey with data on health and health beliefs from survey Wave 5 in 2013,^15^^,^^16^ which is the most recent wave including physical health perception measures. We use additional information from Wave 2 in 2007 to show that health perception is rather stable across waves in Supplementary Appendix S2, enabling the usage of two different survey waves for the analysis. Thus far, the SHARE COVID-19 survey has been conducted only once, therefore enabling cross-sectional analyses only.

SHARE data are *ex ante* harmonized, allowing us to analyze 13 European countries that participated in both the COVID-19 survey and in survey Wave 5, namely, Belgium, Czechia, Denmark, Estonia, France, Germany, Italy, Luxembourg, Slovenia, Spain, Sweden, Switzerland and the Netherlands. We exclude observations based on proxy respondents: these results in 24 507 Europeans aged at least 50 years in 2013 who participated in both Waves 5 and 8. The survey was reviewed and approved by the Ethics Committee of the University of Mannheim and the Ethics Council of the Max Planck Society.^17^

#### Outcome variables: adaptive behaviour

We differentiate between three types of COVID-19-related adaptive behaviours, namely, behaviours related to reduced mobility, protection in public spaces and hygiene measures. Reduced mobility includes staying at home, doing less shopping, less going for walks, less meetings with more than five people from outside the household and less visits to or from other family members. Protection in public spaces refers to adaptive behaviour that has often been mandated or recommended by law; in particular, wearing masks in public spaces and keeping distance when outside the home. Hygiene measures include washing, sanitizing or disinfecting hands (for cross-tabulations with health perception, see table 2; for a detailed sequence of survey questions, see Supplementary Appendix table SA1, and for summary statistics Supplementary Appendix table SA2). All outcome variables are binary and coded so that 1 represents adaptive behaviour. As some adaptive behaviour variables originally had ordered outcomes and were dichotomized (see Supplementary Appendix table SA1 for details), we conduct robustness analyses that consider the original outcomes. All robustness analyses and their results are explained and discussed in detail in Supplementary Appendix S2.

**Table 2:** Cross-tabulations of health perception and COVID-19-related adaptive behaviour

	1	2	3	4	5
	Total (%)	Positive concordance (%)	Underestimating (%)	Negative concordance (%)	Overestimating (%)
Reduced mobility					
Staying home	12.1	9.9	14.3	31.8	21.8
Less shopping	69.2	67.4	70.6	83.8	79.4
Less walks	52.2	49.3	54.8	72.5	71.1
Less meetings	91.1	90.5	92.2	93.5	94.3
Less visit	84.0	83.3	84.1	88.1	89.8
Protection in public space					
Wearing masks	79.3	78.7	80.9	82.5	84.0
Keeping distance	96.1	96.3	95.6	94.1	96.4
Hygiene measures					
Washing hands	87.2	88.2	84.8	76.9	86.4
Sanitizing hands	80.6	81.3	77.6	73.2	83.2

Notes: Calibrated cross-sectional individual weights are applied; in Column 1, 100% refers to the total number of observations; in Columns 2–5, 100% refers to the number of observations in the respective health perception category; variables ‘less shopping’, ‘less walks’, ‘less meetings’ and ‘less visits’ are only asked to those who did not stay at home and thus for these variables the category ‘adaptation’ includes the share of individuals who did not leave their home (for more details see Supplementary Appendix table SA1).

#### Explanatory variable: health perception

We operationalize health perception using a well-established measure that considers the difference between subjective and objective health.^9^^,^^11^^,^^18^ More specifically, we compare survey respondents’ self-reported and tested ability to stand up from a chair. Those whose self-reported ability to stand up from a chair matches their outcome during the chair stand test are considered to have achieved either positive concordance (i.e. they are able to stand up from a chair according to both the self-report and the test) or negative concordance (i.e. they are unable to do this according to both the self-report and the test)— see table 1 for an overview. Individuals who report being able to stand up from a chair but are unable to do so during a chair stand test are classified as overestimating their health, whereas those who report being unable to stand up from the chair but can do so during the performance test are considered as underestimating their health. This health perception measure is explained and evaluated in detail in Refs.^9^^,^^18^

**Table 1:** Overview of health perception categories and number of observations per category

	Objectively			
Subjectively	Able		Unable	
Able	Positive concordance	18 746 (87.2%)	Overestimating	1416 (47.1%)
Unable	Underestimating	2756 (12.8%)	Negative concordance	1589 (52.9%)
Total		21 502 (100%)		3005 (100%)

Note: No weights applied.

### Estimation strategy

Taking into account the binary nature of the outcome variables, the association between individual health perception and adaptive behaviour is estimated based on the following logistic regression model:

$$\text{logit}\left(\pi_{i}\right|)=\alpha \;+\;\beta {\mathrm{HEALTH}\;\mathrm{PERCEPTION}}_{i}+\;\gamma X_{i}+{\delta C}_{i}+\;{\mathrm{µW}}_{i}$$

where $\pi_{i}$ indicates the probability of individual *i* showing adaptive behaviour according to the respective outcome, and ${\mathrm{HEALTH}\;\mathrm{PERCEPTION}}_{i}$ indicates its health belief. The vector of control variables $X_{i}$ includes age, age squared, gender and educational attainment. Additionally, we control for health status using variables that indicate if the individual has any chronic diseases, limitations in activities of daily living (ADLs) or instrumental activities of daily living (IADLs), whether the individual is frail according to a frailty index,^19^^,^^20^ and also cognitive ability. For robustness analyses, we further control for the respondent’s retirement status, relationship status, level of depressive symptoms, household income and whether they or anyone close to them has tested positive for the COVID-19 virus (Supplementary Appendix tables SA9 and SA10). More details on the construction of these control variables are given in ‘Construction of control variables section'.

As the severity of the pandemic as well as mandatory rules relating to it vary between countries and over time, we also include control dummies for the country of residence (*C*_*i*_) and for survey interview week (*W*_*i*_) in the regression model, and interact them in robustness analyses (Supplementary Appendix tables SA13 and SA14). Moreover, we utilize country-level data on public response measures to further explore the effect of pandemic-related health policies (Supplementary Appendix tables SA15 and SA16). This research article, however, does not focus on country differences in the results.

Individuals who are able to stand up from a chair might have very different characteristics from individuals unable to do so, which potentially affects their health behaviour. To account for such heterogeneities and in line with previous analyses,^18^ we further split our sample based on the tested ability to stand up from a chair. Hence, individuals who underestimate their health are always compared with those with positive concordance (i.e. able to stand up from the chair according to self-reports and performance test), and individuals who overestimate their health are always compared with those with negative concordance (i.e. unable to stand up from a chair according to self-reports and performance test).

The dependent variables are taken from the COVID-19 survey (i.e. Wave 8) and all explanatory variables are taken from Wave 5 when the performance test was conducted, except for the interview week, which also refers to the COVID-19 survey.

#### Construction of control variables

We control for a variety of potential confounders in our main analysis and include additional control variables for robustness analyses. Summary statistics for all variables are given in Supplementary Appendix table SA2.

First, we control for age in single years and also include age squared to account for potential non-linearities. Individuals included in the analysis are aged 50–94, with mean age 62. Second, we control for the survey respondent’s gender—52.6% of the survey participants are female. Third, we account for the respondent’s highest educational attainment, differentiating between low, medium and high education according to the International Standard Classification of Education. Fourth, underlying health status does not confound our findings, as we control for physical and cognitive health in multiple ways. We include a binary variable that indicates whether the survey respondent is suffering from at least one of the following chronic diseases: heart attack, high blood pressure or hypertension, high blood cholesterol, a stroke or cerebral vascular disease, diabetes or high blood sugar, chronic lung disease such as chronic bronchitis or emphysema, cancer or malignant tumour, stomach or duodenal ulcer, Parkinson’s Disease, cataracts, hip fracture, other fractures, Alzheimer’s Disease, dementia, organic brain syndrome, senility or any other serious memory impairment, other affective or emotional disorders, including anxiety, nervous or psychiatric problems, rheumatoid arthritis, osteoarthritis or other forms of rheumatism. In our sample, 67% suffer from at least one of these conditions.

We also include a binary variable that reflects whether the respondent has any limitations in ADLs (dressing, eating, using the toilet, bathing and showering, getting in and out of bed, walking across a room) and IADLs (preparing a hot meal, shopping for groceries, making telephone calls, taking medications, managing money, such as paying bills and keeping track of expenses). In the sample, 7.1% of the respondents have at least one limitation in ADLs, and 9.6% have at least one limitation in IADLs.

A well-established frailty indicator is also included in the regression equation.^19^ It considers exhaustion, shrinking, weakness, slowness, and low physical activity and was adapted for SHARE in,^20^ where it is explained in detail. According to this indicator, 7.5% of the samples are considered frail. In addition to physical health, we also account for cognitive ability by adding a control variable that describes orientation to date. This is a categorical variable ranging from zero to four, which measures if the survey respondent knows the day of the week, day of the month, months and year at the time of the survey interview. The average score across the sample is 3.75.

For robustness analyses, we also control for the individual’s retirement status, relationship status, depressive symptoms, whether they or anyone close to them has tested positive for the COVID-19 virus, and the lowest monthly household income during the pandemic. For the retirement status, we create a binary variable that is one if the observation is retired, including semi-retired, partially retired or in early retirement. According to this variable, 40.0% of the samples are in retirement. We also account for the relationship status with a variable that is one if the individual is living with their partner. This variable is chosen as it includes non-married couples but excludes older adults that do not live together anymore, for example, because one spouse has moved to a nursing home. A total of 67.6% of the participants are living with their partner. We also add a variable indicating if the respondent felt sad or depressed in the month prior to the interview—29.1% of the sample showed depressive symptoms. We also add a variable that reflects whether the respondent or anyone close to the respondent has tested positive for the COVID-19 virus, as such a diagnosis is likely to affect adaptive behaviour during the pandemic. This dummy variable is one for 10.8% of the respondents.

Finally, we control for the household’s lowest overall monthly income since the outbreak of the pandemic. In particular, the survey respondent is asked ‘What was the lowest overall monthly income, after taxes and contributions that your entire household had, including any financial support you may have received since the outbreak of Corona?’ We equivalize the reported income using the square root scale, for which household income is divided by the square root of household size. To account for the skewed income distribution, we further apply a cube root transformation to this variable.

#### Summary of robustness analyses

We provide a range of robustness analyses that are explained in detail in Supplementary Appendix S2 and that consider different specifications of health perception, different estimation methods, additional control variables and heterogeneity analyses. First, we analyze how time-constant health beliefs are across waves. Second, as some adaptive behaviour variables originally had ordered outcomes and were dichotomized (Supplementary Appendix table SA1), we conduct robustness analyses considering the original outcomes and run ordered logit regressions instead of logistic regressions (Supplementary Appendix tables SA5 and SA6). Third, for the original health perception variable, individuals who had to use their arm to help them stand up from the chair during the performance test were considered unable (see 9 and 18 for details on the health perception measure). For the robustness analyses in Supplementary Appendix tables SA7 and SA8, these individuals are considered able to stand up from a chair. Fourth, we also consider whether respondents are retired, living in a partnership, suffer from depression and whether they or anyone close to them has tested positive for the COVID-19 virus using additional control variables (Supplementary Appendix tables SA9 and SA10). We also control for the lowest equivalized monthly household income during the pandemic but we do this in a separate regression, as this variable is missing for over 23% of the survey respondents (Supplementary Appendix tables SA11 and SA12). Finally, we also interact the country and survey week dummies (Supplementary Appendix tables SA13 and SA14), and control for pandemic-related public health policies at the time of the interview (Supplementary Appendix tables SA15 and SA16) to further account for variations between countries and over time in the severity of the pandemic as well as in the mandatory rules.

## Results

Column 1 in table 2 provides descriptive statistics, which suggest that most older adults engage in preventive actions, especially with respect to protection in public spaces and hygiene measures. The most common preventive behaviours are keeping distance (96.1%) and fewer meetings (91.1%), whereas only a small proportion of individuals stayed at home during the pandemic (12.1%). Adaptive behaviour varies, however, across health perception groups (Columns 2–5, table 2). Most importantly, individuals who underestimated their health reduced their mobility more and were more likely to wear masks than their comparison groups (i.e. positive concordance). The pattern is less clear for other types of protection in public spaces and hygiene measures, and also among those who overestimated their health.

**Figure 1:**
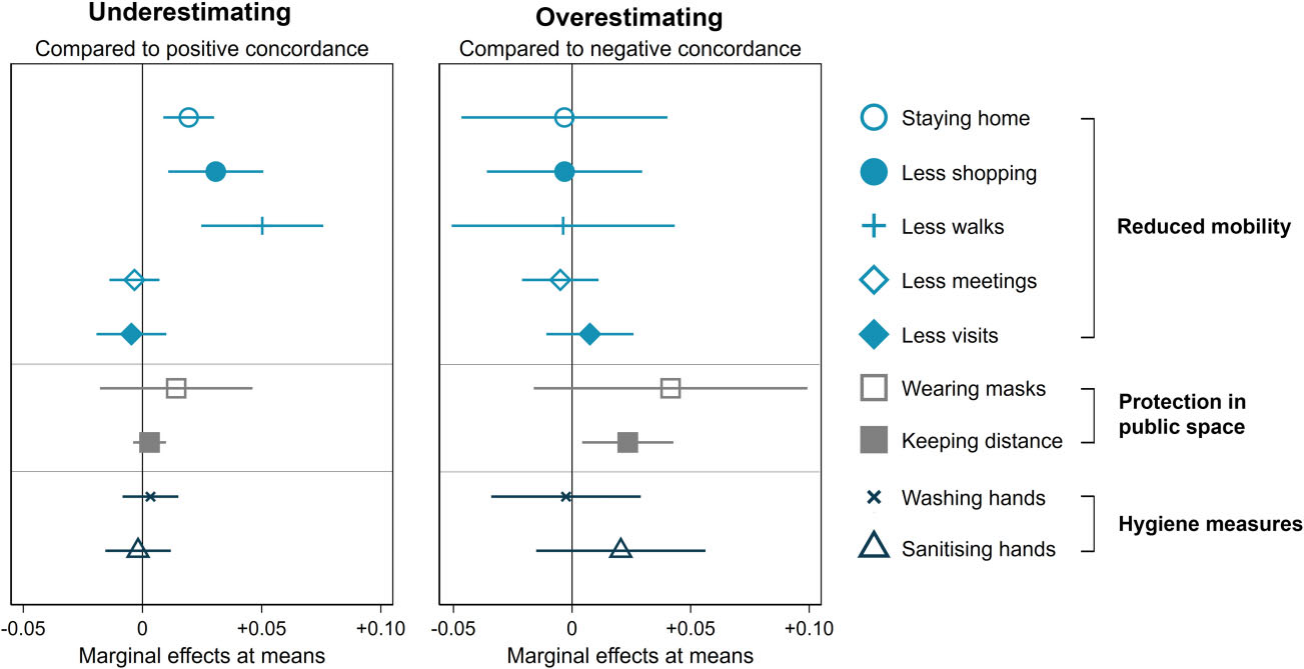
Marginal effects (at means) of health perception on COVID-19-related adaptive behaviour (the underlying estimation results, including coefficients and standard errors, are provided in Supplementary Appendix tables SA3 and SA4)

Figure 1 shows the logistic regression results, which confirm the descriptive findings for mobility reductions. Relative to older adults who accurately assess their health, those who underestimate are significantly more likely to stay at home, more likely to reduce shopping and more likely to reduce going for walks. No significant association between underestimation and protection in public spaces or hygiene measures is found. Contrary to our hypothesis, those who overestimate their health are significantly more likely to keep distance when outside their home. We do not, however, find a statistically significant relationship between overestimation of health and any of the other preventive behaviours.

Note that underlying health status does not confound these findings, as we control for physical and cognitive health in multiple ways and split the sample based on the tested ability to stand up from a chair. Additionally, we control for differences in adaptive pandemic behaviour by age, gender and education. Supplementary Appendix tables SA3 and SA4 show the full set of coefficients. In addition to our main results, we find that women—who overall are more likely to underestimate their health (12.5%) than men (6.5%)—engage significantly more in adaptive behaviours. Moreover, preventive health behaviour increases with higher age for most measures, and older adults with higher education are more likely to adopt protection in public spaces and hygiene measures but less likely to reduce their mobility, compared with the less educated.

Our results are robust to a range of robustness analyses, described in Supplementary Appendix S2, that consider different specifications of health perception, different estimation methods, additional control variables and interactions.

## Discussion

Although older adults are more vulnerable during the COVID-19 pandemic^2–4^^,^^21^ and perceive the risk of the virus to be higher than younger adults do,^22^ studies frequently show that older adults fail to engage in protective health behaviour.^6–8^ One explanation might be the increase in health misperception with age,^9^ leading to deviations from recommended guidelines. In this article, we hypothesized that individuals underestimating their health engage more in preventive action, whereas individuals overestimating their health engage less.

Our results show that older adults who underestimate their health are indeed more likely to exercise adaptive behaviour, at least concerning mobility reductions. More specifically, they are more likely to stay at home, shop less and go for walks less often. The relationship might operate through increased perception of disease susceptibility and severity.^12^ Although these preventive actions may protect individuals and help to contain the virus, an exaggerated reduction in mobility on the part of those who underestimate their health might be contributing to the already high social isolation and loneliness of older adults during the ongoing pandemic.^23–25^ In additional analyses, we also find that depressive symptoms are associated with an increase in adaptive behaviour (Supplementary Appendix tables SA9 and SA10).

Contrary to our hypothesis, adaptive behaviour related to protection in public spaces and hygiene measures are not significantly associated with underestimating health. One possible explanation is that protection in public spaces was often mandatory and was thus exercised regardless of health perception. Overall, we find that differences in health perception matter less for preventive behaviours that are widely adopted, such as hygiene measures (table 2). Moreover, the SHARE survey questions on hygiene measures are phrased so that they ask whether respondents washed, sanitized or disinfected their hands more frequently than usual, (i.e. if they increased their hygiene behaviour) (Supplementary Appendix table SA1). If individuals who underestimate their health had already adopted such hygiene measures before the pandemic, their higher baseline level could explain why they did not (have to) further increase their hygiene measures, which is why we find no effect of underestimating health on hygiene measures during the pandemic.

Overestimating health appears not to be significantly associated with preventive behaviour. The only exception is that those who overestimate their health appear more likely to keep distance when outside, which is contrary to our hypothesis. Speculatively, those who overestimate their health might also be overconfident in other domains (i.e. their preventive behaviour) or even overestimate distances themselves, which could explain this curious finding. Moreover, the positive effect becomes insignificant once the original ordered structure of the outcome variable is considered by employing ordered logit regressions instead of logistic regressions for binary outcomes (Supplementary Appendix table SA6).

In addition to our main results, we find that women in good health are more likely to engage in protective behaviours than men. This is in line with recent studies showing greater COVID-19-related risk awareness among women,^22^^,^^26^ who in turn may be more likely to implement protective behaviour.^27^

One potential limitation of this study is that data on health perception is taken from 2013, because the SHARE COVID-19 study did not cover the performance tests needed for the health perception measure. We have shown, however, that individual health perception is generally stable over time and we also control for age as a potential confounder. Furthermore, this analysis does not elicit the causal effects of health perception on adaptive behaviour, which could be desirable for future work.

In conclusion, our study showed that health perception is relevant for the adoption of preventive health behaviour among older Europeans, especially with respect to mobility reductions. Future work could fruitfully explore a potential link between underestimating health, mobility reduction and increases in social isolation and loneliness. 

## Supplementary data

Supplementary data are available at *EURPUB* online. 

## Funding

This work was supported by the Austrian Academy of Sciences via an APART-GSK Fellowship for Daniela Weber. This article uses data from SHARE Waves 2, 5 and 8 (10.6103/SHARE.w2.710, 10.6103/SHARE.w5.710 and 10.6103/SHARE.w8ca.100). The SHARE data collection has been funded by the European Commission, DG RTD through FP5 (QLK6-CT-2001-00360), FP6 (SHARE-I3: RII-CT-2006-062193, COMPARE: CIT5-CT-2005-028857 and SHARELIFE: CIT4-CT-2006-028812), FP7 (SHARE-PREP: GA No. 211909, SHARE-LEAP: GA No. 227822, SHARE M4: GA No. 261982, DASISH: GA No. 283646) and Horizon 2020 (SHARE-DEV3: GA No. 676536, SHARE-COHESION: GA No. 870628, SERISS: GA No. 654221, SSHOC: GA No. 823782) and by DG Employment, Social Affairs & Inclusion through VS 2015/0195, VS 2016/0135, VS 2018/0285, VS 2019/0332 and VS 2020/0313. Additional funding from the German Ministry of Education and Research, the Max Planck Society for the Advancement of Science, the U.S. National Institute on Ageing (U01_AG09740-13S2, P01_AG005842, P01_AG08291, P30_AG12815, R21_AG025169, Y1-AG-4553-01, IAG_BSR06-11, OGHA_04-064, HHSN271201300071C and RAG052527A) and from various national funding sources is gratefully acknowledged (see www.share-project.org). 

*Conflicts of interest*: None declared.

Key points

- A significant proportion of older adults do not follow recommended guidelines concerning preventive actions during the COVID-19 pandemic.
- Health beliefs appear relevant for the adoption of preventive health behaviours related to mobility, and thus have serious consequences for the containment of the virus.
- Older adults who underestimate their health are more likely to show adaptive behaviour related to mobility reductions.
- Overestimating health is not significantly associated with reduced mobility, protective behaviour in public spaces or hygiene measures.

## Data availability

The data underlying the results presented in the study are available from the Survey of Health, Ageing and Retirement in Europe (http://www.share-project.org).

## Supplementary Material

ckab221_Supplementary_Data
