# Supplementary material for: Older Europeans’ health perception and their adaptive behavior during the COVID-19 pandemic
Source: Eur J Public Health. 2022 Jan 3:ckab221. doi: 10.1093/eurpub/ckab221 (PMC8755393; doi:10.1093/eurpub/ckab221)
Supplement: Supplementary file 1 [file ckab221_supplementary_data.docx]

**Older Europeans’ health perception and their adaptive behaviour during the COVID-19 pandemic**

**SUPPLEMENTARY MATERIAL**

**Appendix 1: main results**

Table A.1 Sequence of survey questions for outcome variables

|  | **Survey question** | **Possible outcomes** | **Routing** | **Transformation** |
| --- | --- | --- | --- | --- |
| 1 | Since the outbreak of Corona, have you ever left your home? | Yes No | Asked to entire sample | Adaptation: no No adaptation: yes |
| 2 | Since the outbreak of Corona, how often have you done the following activities, as compared to before the outbreak? Going shopping? Going out for a walk? Meeting with more than five people from outside your household? Visiting other family members? | Not anymore Less often About the same More often | Asked if question 1 is answered with “yes” | Adaptation: not anymore, less often, if question 1 is answered with “no” No adaptation: about the same, more often |
| 3 | How often did you wear a face mask when you went outside your home to a public space? | Always Often Sometimes  Never | Asked if question 1 is answered with “yes” | Adaptation: always, often No adaptation: sometimes, never |
| 4 | How often did you keep distance to others when you went outside your home? | Always Often Sometimes Never | Asked if question 1 is answered with “yes” | Adaptation: always, often No adaptation: sometimes, never |
| 5 | Did you wash your hands more frequently than usual? | Yes No | Asked to entire sample | Adaptation: yes No adaptation: no |
| 6 | Did you use special hand sanitiser or disinfection fluids more frequently than usual? | Yes No | Asked to entire sample | Adaptation: yes No adaptation: no |

Table A.2: Summary statistics

|  | N | Mean | SD | Min. | Max. |
| --- | --- | --- | --- | --- | --- |
| **Adaptive behaviour** |  |  |  |  |  |
| **Reduced mobility** |  |  |  |  |  |
| Staying home (1 = yes) | 24,399 | 0.121 | 0.326 | 0 | 1 |
| Less shopping (1 = yes) | 24,164 | 0.692 | 0.462 | 0 | 1 |
| Less walks (1 = yes) | 24,060 | 0.522 | 0.500 | 0 | 1 |
| Less meetings (1 = yes) | 23,909 | 0.911 | 0.285 | 0 | 1 |
| Less visits (1 = yes) | 23,944 | 0.840 | 0.367 | 0 | 1 |
| **Protection in public spaces** |  |  |  |  |  |
| Wearing masks (1 = yes) | 20,686 | 0.793 | 0.405 | 0 | 1 |
| Keeping distance (1 = yes) | 20,654 | 0.961 | 0.193 | 0 | 1 |
| **Hygiene measures** |  |  |  |  |  |
| Washing hands (1 = yes) | 24,364 | 0.872 | 0.334 | 0 | 1 |
| Sanitising hands (1 = yes) | 24,373 | 0.806 | 0.395 | 0 | 1 |
| **Health perception** |  |  |  |  |  |
| Positive concordance (1 = yes) | 24,507 | 0.796 | 0.403 | 0 | 1 |
| Underestimating (1 = yes) | 24,507 | 0.097 | 0.296 | 0 | 1 |
| Negative concordance (1 = yes) | 24,507 | 0.047 | 0.212 | 0 | 1 |
| Overestimating (1 = yes) | 24,507 | 0.060 | 0.238 | 0 | 1 |
| **Mobility** |  |  |  |  |  |
| Objectively mobile (1 = yes) | 24,507 | 0.893 | 0.310 | 0 | 1 |
| Subjectively mobile (1 = yes) | 24,507 | 0.856 | 0.351 | 0 | 1 |
| **Control variables** |  |  |  |  |  |
| Age (in number of years) | 24,507 | 61.896 | 9.726 | 50 | 94 |
| Gender (1 = female) | 24,507 | 0.526 | 0.499 | 0 | 1 |
| Low education | 24,162 | 0.363 | 0.481 | 0 | 1 |
| Medium education | 24,162 | 0.393 | 0.488 | 0 | 1 |
| High education | 24,162 | 0.244 | 0.430 | 0 | 1 |
| Chronic disease dummy (1 = yes) | 24,499 | 0.674 | 0.469 | 0 | 1 |
| ADL dummy (1 = yes) | 24,505 | 0.071 | 0.258 | 0 | 1 |
| IADL dummy (1 = yes) | 24,505 | 0.096 | 0.295 | 0 | 1 |
| Frailty (1 = frail) | 23,212 | 0.075 | 0.263 | 0 | 1 |
| Cognitive ability (4 = high) | 24,482 | 3.749 | 0.587 | 0 | 4 |
| Retired (1 = yes) | 24,446 | 0.400 | 0.490 | 0 | 1 |
| Living with partner (1 = yes) | 24,276 | 0.676 | 0.468 | 0 | 1 |
| Feeling depressed or sad (1 = yes) | 24,445 | 0.291 | 0.455 | 0 | 1 |
| Anyone tested positive (1 = yes) | 24,197 | 0.108 | 0.310 | 0 | 1 |
| Lowest monthly household income | 18,612 | 1,802 | 1,525 | 0 | 82,704 |

Note: Calibrated cross-sectional individual weights are applied to all columns except “N”

Table A.3: Main results for underestimating health

|  | (1) | (2) | (3) | (4) | (5) | (6) | (7) | (8) | (9) |
| --- | --- | --- | --- | --- | --- | --- | --- | --- | --- |
|  | Staying home | Less shopping | Less walks | Less meetings | Less visits | Wearing masks | Keeping distance | Washing hands | Sanitising hands |
| Underestimating | 0.255^***^ | 0.167^**^ | 0.202^***^ | -0.054 | -0.044 | 0.070 | 0.106 | 0.040 | -0.016 |
|  | (0.066) | (0.057) | (0.052) | (0.085) | (0.070) | (0.082) | (0.130) | (0.071) | (0.061) |
| Age | -0.023 | -0.041 | -0.113^***^ | 0.208^***^ | 0.136^***^ | 0.116^*^ | 0.241^***^ | 0.189^***^ | 0.083^**^ |
|  | (0.039) | (0.030) | (0.030) | (0.044) | (0.036) | (0.046) | (0.058) | (0.035) | (0.032) |
| Age squared | 0.001^*^ | 0.001^*^ | 0.001^***^ | -0.001^***^ | -0.001^***^ | -0.001^*^ | -0.002^***^ | -0.002^***^ | -0.001^***^ |
|  | (0.000) | (0.000) | (0.000) | (0.000) | (0.000) | (0.000) | (0.000) | (0.000) | (0.000) |
| Women | 0.264^***^ | 0.644^***^ | 0.268^***^ | 0.406^***^ | 0.324^***^ | 0.413^***^ | 0.408^***^ | 0.224^***^ | 0.118^**^ |
|  | (0.045) | (0.032) | (0.031) | (0.049) | (0.040) | (0.045) | (0.075) | (0.044) | (0.037) |
| Medium education (ref. = low) | -0.324^***^ | -0.041 | -0.210^***^ | -0.036 | -0.073 | 0.168^*^ | 0.132 | 0.075 | 0.116^*^ |
|  | (0.057) | (0.045) | (0.042) | (0.066) | (0.056) | (0.066) | (0.094) | (0.057) | (0.051) |
| High education (ref. = low) | -0.647^***^ | 0.030 | -0.428^***^ | 0.185^*^ | 0.015 | 0.368^***^ | 0.465^***^ | 0.327^***^ | 0.192^***^ |
|  | (0.073) | (0.049) | (0.047) | (0.076) | (0.062) | (0.071) | (0.113) | (0.067) | (0.058) |
| Chronic disease dummy | 0.082 | 0.181^***^ | 0.241^***^ | 0.079 | 0.096^*^ | 0.238^***^ | 0.154 | -0.031 | 0.067 |
|  | (0.055) | (0.037) | (0.037) | (0.055) | (0.046) | (0.053) | (0.086) | (0.052) | (0.046) |
| ADL dummy | -0.053 | 0.029 | 0.216^**^ | 0.181 | 0.304^**^ | -0.071 | -0.295 | -0.023 | 0.011 |
|  | (0.104) | (0.087) | (0.081) | (0.136) | (0.118) | (0.119) | (0.179) | (0.101) | (0.090) |
| IADL dummy | 0.291^***^ | 0.144 | 0.250^***^ | 0.021 | 0.054 | -0.043 | -0.143 | -0.238^**^ | -0.049 |
|  | (0.082) | (0.074) | (0.069) | (0.112) | (0.093) | (0.099) | (0.158) | (0.083) | (0.077) |
| Frailty dummy | 0.472^***^ | 0.217^*^ | 0.645^***^ | 0.642^***^ | 0.306^*^ | 0.287^*^ | -0.211 | -0.121 | -0.303^***^ |
|  | (0.085) | (0.097) | (0.089) | (0.173) | (0.128) | (0.135) | (0.184) | (0.101) | (0.087) |
| Cognition score = 1 (ref. = 0) | -0.129 | 0.252 | -0.480 | -0.127 | -0.528 | -0.010 | -1.519^*^ | -0.401 | -0.673 |
|  | (0.388) | (0.472) | (0.479) | (0.809) | (0.569) | (0.800) | (0.753) | (0.381) | (0.363) |
| Cognition score = 2 (ref. = 0) | -0.749^**^ | -0.170 | -0.615^*^ | -0.430 | -0.241 | -0.194 | -0.734 | 0.213 | -0.130 |
|  | (0.270) | (0.283) | (0.310) | (0.427) | (0.382) | (0.461) | (0.581) | (0.270) | (0.267) |
| Cognition score = 3 (ref. = 0) | -0.925^***^ | -0.192 | -0.723^**^ | -0.369 | -0.339 | 0.115 | -0.210 | 0.660^**^ | 0.122 |
|  | (0.225) | (0.246) | (0.272) | (0.363) | (0.331) | (0.402) | (0.524) | (0.225) | (0.229) |
| Cognition score = 4 (ref. = 0) | -1.033^***^ | -0.205 | -0.774^**^ | -0.380 | -0.417 | 0.209 | -0.154 | 0.685^**^ | 0.225 |
|  | (0.220) | (0.244) | (0.270) | (0.358) | (0.327) | (0.397) | (0.516) | (0.220) | (0.225) |
| Constant | -3.721^**^ | 0.494 | 2.056^*^ | -5.304^***^ | -3.483^**^ | -1.763 | -4.107^*^ | -4.554^***^ | -0.956 |
|  | (1.332) | (1.011) | (1.000) | (1.451) | (1.192) | (1.549) | (2.001) | (1.169) | (1.080) |
| Country dummies | Yes | Yes | Yes | Yes | Yes | Yes | Yes | Yes | Yes |
| Interview week dummies | Yes | Yes | Yes | Yes | Yes | Yes | Yes | Yes | Yes |
| N | 20,510 | 20,331 | 20,242 | 20,104 | 20,141 | 17,901 | 17,880 | 20,488 | 20,496 |
| Pseudo R2 | 0.184 | 0.092 | 0.210 | 0.080 | 0.060 | 0.524 | 0.047 | 0.035 | 0.068 |

Note: Logistic regressions based on the sample that was able to stand up during the chair stand test; the table presents coefficients along with standard errors in parentheses; * p<0.05, ** p<0.01, *** p<0.0

Table A.4: Main results for overestimating health

|  | (1) | (2) | (3) | (4) | (5) | (6) | (7) | (8) | (9) |
| --- | --- | --- | --- | --- | --- | --- | --- | --- | --- |
|  | Staying home | Less shopping | Less walks | Less meetings | Less visits | Wearing masks | Keeping distance | Washing hands | Sanitising hands |
| Overestimating | -0.018 | -0.027 | -0.019 | -0.129 | 0.149 | 0.255 | 0.614^*^ | -0.021 | 0.142 |
|  | (0.126) | (0.140) | (0.123) | (0.217) | (0.183) | (0.177) | (0.249) | (0.133) | (0.125) |
| Age | 0.026 | -0.077 | -0.081 | 0.089 | 0.236^*^ | 0.061 | 0.379^**^ | 0.117 | 0.106 |
|  | (0.085) | (0.097) | (0.080) | (0.132) | (0.105) | (0.129) | (0.143) | (0.084) | (0.078) |
| Age squared | 0.000 | 0.001 | 0.001 | -0.000 | -0.002^*^ | -0.001 | -0.003^**^ | -0.001 | -0.001^*^ |
|  | (0.001) | (0.001) | (0.001) | (0.001) | (0.001) | (0.001) | (0.001) | (0.001) | (0.001) |
| Women | 0.479^***^ | 0.709^***^ | 0.424^***^ | 0.290 | 0.165 | 0.587^***^ | 0.552^*^ | 0.386^**^ | 0.200 |
|  | (0.120) | (0.122) | (0.109) | (0.193) | (0.164) | (0.164) | (0.221) | (0.123) | (0.116) |
| Medium education (ref. = low) | -0.382^**^ | -0.127 | 0.065 | -0.088 | 0.046 | -0.172 | 0.440 | -0.259 | 0.231 |
|  | (0.135) | (0.145) | (0.125) | (0.224) | (0.195) | (0.191) | (0.245) | (0.146) | (0.134) |
| High education (ref. = low) | -0.594^**^ | 0.055 | -0.176 | 0.170 | -0.215 | 0.090 | 0.246 | -0.089 | 0.203 |
|  | (0.184) | (0.183) | (0.154) | (0.292) | (0.238) | (0.223) | (0.319) | (0.184) | (0.173) |
| Chronic disease dummy | -0.007 | 0.153 | 0.187 | 0.576^*^ | 0.002 | 0.315 | 0.079 | 0.129 | 0.147 |
|  | (0.164) | (0.168) | (0.150) | (0.230) | (0.236) | (0.213) | (0.295) | (0.175) | (0.164) |
| ADL dummy | 0.331^*^ | 0.402^*^ | 0.288 | 0.470 | -0.023 | 0.299 | 0.610^*^ | -0.079 | -0.163 |
|  | (0.140) | (0.187) | (0.158) | (0.304) | (0.239) | (0.231) | (0.308) | (0.143) | (0.140) |
| IADL dummy | 0.328^*^ | 0.395^*^ | 0.318^*^ | 0.113 | 0.274 | -0.174 | -0.100 | -0.306^*^ | -0.100 |
|  | (0.134) | (0.175) | (0.144) | (0.276) | (0.244) | (0.206) | (0.268) | (0.145) | (0.140) |
| Frailty dummy | 0.255^*^ | 0.290 | 0.502^***^ | 0.068 | 0.276 | -0.081 | -0.205 | -0.294^*^ | 0.051 |
|  | (0.129) | (0.165) | (0.137) | (0.248) | (0.211) | (0.198) | (0.256) | (0.137) | (0.128) |
| Cognition score = 1 (ref. = 0) | -0.471 | 0.675 | 0.182 | 0.005 | -0.662 | -0.398 | -0.880 | -0.290 | 0.379 |
|  | (0.581) | (1.220) | (0.858) | (1.140) | (1.291) | (1.119) | (1.347) | (0.619) | (0.613) |
| Cognition score = 2 (ref. = 0) | -0.427 | 0.732 | 0.477 | 0.579 | 0.045 | 0.846 | 0.000 | 0.117 | 0.195 |
|  | (0.446) | (0.738) | (0.631) | (0.731) | (0.949) | (0.987) | (.) | (0.436) | (0.445) |
| Cognition score = 3 (ref. = 0) | -1.248^**^ | 0.056 | -0.425 | 0.033 | -0.556 | 0.474 | 0.463 | 0.375 | 0.142 |
|  | (0.385) | (0.578) | (0.506) | (0.248) | (0.770) | (0.811) | (0.743) | (0.374) | (0.384) |
| Cognition score = 4 (ref. = 0) | -1.465^***^ | -0.255 | -0.504 | 0.000 | -0.443 | 0.592 | 0.986 | 0.634 | 0.388 |
|  | (0.373) | (0.558) | (0.495) | (.) | (0.757) | (0.790) | (0.709) | (0.359) | (0.372) |
| Constant | -4.995 | 1.555 | 0.583 | -2.657 | -6.809 | 0.105 | -10.359^*^ | -2.177 | -1.905 |
|  | (2.952) | (3.198) | (2.700) | (4.321) | (3.561) | (4.327) | (4.932) | (2.903) | (2.675) |
| Country dummies | Yes | Yes | Yes | Yes | Yes | Yes | Yes | Yes | Yes |
| Interview week dummies | Yes | Yes | Yes | Yes | Yes | Yes | Yes | Yes | Yes |
| N | 2,260 | 2,219 | 2,224 | 2,118 | 2,192 | 1,580 | 1,464 | 2,250 | 2,255 |
| Pseudo R2 | 0.240 | 0.155 | 0.218 | 0.103 | 0.123 | 0.444 | 0.128 | 0.061 | 0.069 |

Note: Logistic regressions based on the sample that was unable to stand up during the chair stand test; the table presents coefficients along with standard errors in parentheses; * p<0.05, ** p<0.01, *** p<0.0

**Appendix 2: robustness analyses**

Our results are robust to a range of robustness analyses that consider different specifications of health perception, different estimation methods, additional control variables including macro drivers of adaptive health, and interactions. First, as some adaptive behaviour variables originally had ordered outcomes and were dichotomised (see Table A.1 in Appendix 1), we conduct robustness analyses considering the original outcomes and run ordered logit regressions instead of logistic regressions for this subset of outcome variables (Tables A.5 and A.6). Although the coefficients for underestimating health vary slightly in magnitude, the results are qualitatively similar to the main analysis. Interestingly, the curious positive effect of overestimating health on keeping distance when outside disappears when ordered logit regressions are employed.

Second, for the original health perception variable, individuals who had to use their arm to help them stand up from the chair during the performance test were considered unable (see 1 or 2 for details on the health perception measure). For the robustness analyses in Tables A.7 and A.8, these individuals are considered able to stand up from a chair. The results are virtually identical to the main analysis.

Third, we also consider additional control variables (Tables A.9 and A.10). Most importantly, we control for depressive symptoms. While the depressive realism hypothesis conjectures that depressive individuals may actually be more accurate and realistic than non-depressed individuals, the selective processing hypothesis suggests that depression may lead to negative bias and under-confidence. Theoretically, the relationship is complex, and experimental evidence adds to this complexity. While a range of early experimental evidence shows that depressed individuals are more realistic than their non-depressed counterparts (3), recent research finds that depressed individuals are slightly more likely to be under-confident in their performance evaluations (4). Furthermore, major cognitive theories of depression point towards a reverse relationship such that negative perceptions of self can cause depression (5,6). It is therefore unclear how depression may actually affect both health perception and adaptive behaviour, or whether the relationship is in the other direction such that staying at home affects depression.

The main results of underestimating and overestimating health on adaptive behaviour remain unchanged when controlling for depression. In addition, we find that individuals with depressive symptoms also appear more likely to show adaptive behaviour. Our results also remain robust to adding retirement status, partnership status, and a variable indicating whether the survey participants or anyone close to them has tested positive for the COVID-19 virus. While living with a partner increases the likelihood of almost all types of preventive behaviour, retirement and positive COVID-19 experiences affect only some of the outcome variables (Tables A.9 and A.10). 

Depending on their economic resources, households might be more or less likely to adapt their behaviour to the ongoing pandemic. We thus also control for the lowest equivalised monthly household income during the pandemic but we do this in a separate regression, as this variable is missing for over 23% of the survey respondents (Tables A.11 and A.12). Our main results are, again, robust to this additional control variable. We find, however, that the effect of income on preventive health behaviour varies across measures. While mobility reductions decrease with higher income, protection in public spaces and hygiene measures increase.

Fourth, we also account for differences in the severity of the pandemic as well as in mandatory rules across countries and time. We interact the country and survey week dummies to further control for variations across time and space (Tables A.13 and A.14). The results remain virtually identical to the main analysis. As public health and social measures enforced at the time of the interview might be important macro drivers of adaptive health behaviour, we also control for policy variations across countries and times during the study period. For this robustness analysis, we utilise data from the European Centre for Disease Prevention and Control (7) which includes selected national public response measures to COVID-19 on a weekly basis. In particular, we control for mandatory mask use in public spaces to account for macro drivers of protection in public spaces, and for the restriction of private gatherings to account for macro drivers of reduced mobility. Variables that could proxy macro drivers of hygiene measures are not available, as washing or sanitising hands was not enforced by law but rather recommended or socially regulated. 

Results in Tables A.15 to A.16 show that our main findings are robust for the inclusion of health and social measures at the country level (i.e., individuals who underestimate their health are more likely to show adaptive behaviour). Moreover, the macro controls have little effect on individual adaptive health behaviour, most likely because country-level variations are already accounted for due to the inclusion of country dummies and controls for the week of the interview. The only additional effect found suggests that mandatory mask use in public spaces decreases mobility reductions related to shopping, potentially because older adults feel that it is safer to go shopping when mandatory mask use applies.

Finally, we investigate if and how health perception changes over time. For our main analysis, we match information on health and health beliefs from wave 5 in 2013 with data on health behaviour from the COVID-19 survey in 2020. It is thus important to investigate how frequently individuals change their health beliefs between waves. Unfortunately, the 2020 COVID-19 survey does not include tested health variables and thus does not allow us to analyse health beliefs. We can, however, analyse variations in health perception between wave 2 in 2007, which also includes the chair stand test, and wave 5 in 2020.

A total of 13,595 individuals participated in both waves 2 and 5, which is a much smaller and potentially selected sample than the one used for the main analysis. This subsample allows us, however, to explore how health perception varies over time. It appears that health beliefs are constant between the waves for the large majority of observations, namely 61.7%, thus reassuring us that our health perception measure is valid for a large fraction of the sample. The within-individual standard deviation in health perception is only 0.10 for the objectively able sample (individuals that are able to stand up from a chair during the chair stand test) and 0.08 for the objectively unable (individuals that are unable to stand up from a chair during the chair stand test).

**REFERENCES**

1.		Spitzer S, Weber D. Reporting biases in self-assessed physical and cognitive health status of older Europeans. PLoS One. 2019;14(10):e0223526. 

2.	Spitzer S, Shaikh M. Health misperception and healthcare utilisation among older Europeans. Vienna Inst Demogr Work Pap. 2020;(01).

3.	Alloy LB, Abramson LY. Judgment of contingency in depressed and nondepressed students: Sadder but wiser? Journal of Experimental Psychology. 1979; 108(4): 441–48.

4.	Fu T, Koutstaal W. Depression, confidence, and decision:  Evidence against depressive realism. Journal of Psychopathology and Behavioral Assessment. 2005; Vol. 27: 243–252.

5.	Beck AT. Depression: Clinical, experimental, and theoretical aspects. 1967. New York: Harper & Row.

6.	Beck AT, Rush AJ, Shaw BF, Emery G. Cognitive therapy of depression.  1979. New York: Guilford.

7.	European Centre for Disease Prevention and Control. Data on country response measures to COVID-19. Release version: November 2021. Data set. 2021; Available from: https://www.ecdc.europa.eu/en/publications-data/download-data-response-measures-covid-19.

Table A.5: Robustness analysis for underestimating health: ordered logit estimations

|  | (1) | (2) | (3) | (4) | (5) | (6) |
| --- | --- | --- | --- | --- | --- | --- |
|  | Less shopping | Less walks | Less meetings | Less visits | Wearing masks | Keeping distance |
| Underestimating | 0.174^***^ | 0.241^***^ | 0.026 | 0.086 | 0.068 | 0.001 |
|  | (0.045) | (0.046) | (0.051) | (0.048) | (0.063) | (0.066) |
| Age | -0.177^***^ | -0.134^***^ | 0.087^**^ | 0.059^*^ | 0.116^***^ | 0.155^***^ |
|  | (0.025) | (0.024) | (0.027) | (0.026) | (0.034) | (0.034) |
| Age squared | 0.002^***^ | 0.001^***^ | -0.000 | -0.000 | -0.001^***^ | -0.001^***^ |
|  | (0.000) | (0.000) | (0.000) | (0.000) | (0.000) | (0.000) |
| Women | 0.483^***^ | 0.120^***^ | 0.305^***^ | 0.274^***^ | 0.429^***^ | 0.388^***^ |
|  | (0.027) | (0.025) | (0.028) | (0.026) | (0.034) | (0.037) |
| Medium education (ref. = low) | -0.162^***^ | -0.215^***^ | -0.120^**^ | -0.134^***^ | 0.119^*^ | 0.055 |
|  | (0.037) | (0.036) | (0.041) | (0.039) | (0.050) | (0.051) |
| High education (ref. = low) | -0.145^***^ | -0.534^***^ | -0.179^***^ | -0.193^***^ | 0.270^***^ | 0.076 |
|  | (0.040) | (0.040) | (0.044) | (0.041) | (0.054) | (0.055) |
| Chronic disease dummy | 0.153^***^ | 0.187^***^ | 0.051 | 0.073^*^ | 0.202^***^ | 0.046 |
|  | (0.030) | (0.030) | (0.033) | (0.031) | (0.040) | (0.043) |
| ADL dummy | 0.063 | 0.157^*^ | 0.082 | 0.111 | -0.053 | -0.141 |
|  | (0.073) | (0.072) | (0.079) | (0.074) | (0.099) | (0.098) |
| IADL dummy | 0.308^***^ | 0.300^***^ | 0.003 | 0.117 | 0.043 | -0.048 |
|  | (0.062) | (0.061) | (0.065) | (0.063) | (0.078) | (0.085) |
| Frailty dummy | 0.435^***^ | 0.642^***^ | 0.591^***^ | 0.316^***^ | 0.301^**^ | 0.065 |
|  | (0.074) | (0.072) | (0.089) | (0.076) | (0.112) | (0.109) |
| Cognition score = 1 (ref. = 0) | 0.421 | -0.244 | -0.046 | 0.062 | 0.197 | -0.296 |
|  | (0.388) | (0.400) | (0.428) | (0.423) | (0.596) | (0.570) |
| Cognition score = 2 (ref. = 0) | -0.361 | -0.629^*^ | -0.382 | -0.459 | -0.024 | -0.364 |
|  | (0.262) | (0.245) | (0.271) | (0.239) | (0.378) | (0.294) |
| Cognition score = 3 (ref. = 0) | -0.566^*^ | -0.689^**^ | -0.356 | -0.505^*^ | 0.123 | 0.112 |
|  | (0.234) | (0.219) | (0.242) | (0.212) | (0.337) | (0.257) |
| Cognition score = 4 (ref. = 0) | -0.620^**^ | -0.741^***^ | -0.396 | -0.523^*^ | 0.219 | 0.192 |
|  | (0.232) | (0.217) | (0.239) | (0.210) | (0.335) | (0.252) |
| cut1 | -8.253^***^ | -5.351^***^ | -1.651 | -1.418 | 0.873 | 1.032 |
|  | (0.837) | (0.820) | (0.914) | (0.847) | (1.162) | (1.133) |
| cut2 | -4.666^***^ | -2.902^***^ | 1.433 | 1.252 | 2.201 | 2.386^*^ |
|  | (0.836) | (0.820) | (0.910) | (0.846) | (1.162) | (1.129) |
| cut3 | -2.072^*^ | -1.571 | 3.420^***^ | 3.255^***^ | 3.062^**^ | 4.152^***^ |
|  | (0.834) | (0.819) | (0.910) | (0.846) | (1.162) | (1.126) |
| Country dummies | Yes | Yes | Yes | Yes | Yes | Yes |
| Interview week dummies | Yes | Yes | Yes | Yes | Yes | Yes |
| N | 20,333 | 20,243 | 20,106 | 20,143 | 17,903 | 17,882 |
| Pseudo R2 | 0.089 | 0.119 | 0.081 | 0.073 | 0.362 | 0.024 |

Note: Ordered logit regressions based on the sample that was able to stand up during the chair stand test; the table presents coefficients along with standard errors in parentheses; * p<0.05, ** p<0.01, *** p<0.0

Table A.6: Robustness analysis for overestimating health: ordered logit estimations

|  | (1) | (2) | (3) | (4) | (5) | (6) |
| --- | --- | --- | --- | --- | --- | --- |
|  | Less shopping | Less walks | Less meetings | Less visits | Wearing masks | Keeping distance |
| Overestimating | -0.047 | -0.026 | 0.048 | 0.014 | 0.150 | 0.279 |
|  | (0.102) | (0.100) | (0.124) | (0.113) | (0.145) | (0.156) |
| Age | -0.117 | -0.091 | 0.012 | 0.065 | 0.013 | 0.234^*^ |
|  | (0.066) | (0.062) | (0.077) | (0.071) | (0.098) | (0.095) |
| Age squared | 0.001^**^ | 0.001^*^ | 0.000 | -0.000 | -0.000 | -0.002^*^ |
|  | (0.000) | (0.000) | (0.001) | (0.001) | (0.001) | (0.001) |
| Women | 0.486^***^ | 0.375^***^ | 0.328^**^ | 0.189 | 0.463^***^ | 0.288^*^ |
|  | (0.094) | (0.087) | (0.109) | (0.098) | (0.129) | (0.136) |
| Medium education (ref. = low) | -0.258^*^ | -0.025 | 0.020 | -0.064 | -0.120 | 0.166 |
|  | (0.109) | (0.107) | (0.133) | (0.121) | (0.162) | (0.166) |
| High education (ref. = low) | -0.313^*^ | -0.292^*^ | -0.172 | -0.377^**^ | 0.053 | -0.276 |
|  | (0.135) | (0.129) | (0.156) | (0.146) | (0.181) | (0.193) |
| Chronic disease dummy | 0.018 | 0.008 | 0.180 | -0.072 | 0.205 | 0.066 |
|  | (0.122) | (0.120) | (0.145) | (0.138) | (0.173) | (0.180) |
| ADL dummy | 0.369^**^ | 0.239 | 0.268 | 0.249 | 0.205 | 0.389^*^ |
|  | (0.126) | (0.124) | (0.162) | (0.150) | (0.180) | (0.192) |
| IADL dummy | 0.438^***^ | 0.267^*^ | 0.068 | 0.166 | -0.292 | -0.129 |
|  | (0.117) | (0.116) | (0.146) | (0.139) | (0.170) | (0.180) |
| Frailty dummy | 0.333^**^ | 0.489^***^ | 0.436^**^ | 0.201 | 0.067 | -0.213 |
|  | (0.111) | (0.108) | (0.139) | (0.126) | (0.159) | (0.166) |
| Cognition score = 1 (ref. = 0) | -0.433 | -0.082 | 0.176 | 0.217 | -0.311 | 0.544 |
|  | (0.618) | (0.673) | (1.005) | (0.723) | (0.758) | (1.300) |
| Cognition score = 2 (ref. = 0) | -0.332 | 0.177 | 0.554 | 0.140 | 0.207 | 0.050 |
|  | (0.515) | (0.479) | (0.708) | (0.483) | (0.768) | (0.713) |
| Cognition score = 3 (ref. = 0) | -0.879 | -0.539 | -0.683 | -0.235 | 0.438 | 0.140 |
|  | (0.469) | (0.427) | (0.546) | (0.423) | (0.648) | (0.664) |
| Cognition score = 4 (ref. = 0) | -1.149^*^ | -0.674 | -0.928 | -0.264 | 0.478 | 0.581 |
|  | (0.461) | (0.420) | (0.535) | (0.414) | (0.633) | (0.648) |
| cut1 | -6.805^**^ | -3.944 | -3.989 | -1.117 | -2.525 | 4.886 |
|  | (2.229) | (2.091) | (2.603) | (2.406) | (3.329) | (3.247) |
| cut2 | -3.185 | -1.366 | -0.964 | 1.726 | -1.436 | 5.930 |
|  | (2.212) | (2.090) | (2.573) | (2.399) | (3.326) | (3.256) |
| cut3 | -1.023 | -0.152 | 0.756 | 3.474 | -0.700 | 7.208^*^ |
|  | (2.209) | (2.088) | (2.570) | (2.396) | (3.321) | (3.250) |
| Country dummies | Yes | Yes | Yes | Yes | Yes | Yes |
| Interview week dummies | Yes | Yes | Yes | Yes | Yes | Yes |
| N | 2,222 | 2,225 | 2,218 | 2,211 | 1,581 | 1,571 |
| Pseudo R2 | 0.153 | 0.130 | 0.097 | 0.093 | 0.315 | 0.047 |

Note: Ordered logit regressions based on the sample that was unable to stand up during the chair stand test; the table presents coefficients along with standard errors in parentheses; * p<0.05, ** p<0.01, *** p<0.0

Table A.7: Robustness analysis for underestimating health: different specification of objective mobility

|  | (1) | (2) | (3) | (4) | (5) | (6) | (7) | (8) | (9) |
| --- | --- | --- | --- | --- | --- | --- | --- | --- | --- |
|  | Staying home | Less shopping | Less walks | Less meetings | Less visits | Wearing masks | Keeping distance | Washing hands | Sanitising hands |
| Underestimating | 0.240^***^ | 0.170^**^ | 0.208^***^ | -0.055 | -0.046 | 0.079 | 0.094 | 0.033 | -0.015 |
|  | (0.066) | (0.057) | (0.052) | (0.084) | (0.069) | (0.082) | (0.129) | (0.071) | (0.061) |
| Age | -0.024 | -0.045 | -0.113^***^ | 0.204^***^ | 0.134^***^ | 0.115^*^ | 0.244^***^ | 0.194^***^ | 0.087^**^ |
|  | (0.038) | (0.030) | (0.029) | (0.044) | (0.035) | (0.046) | (0.058) | (0.034) | (0.032) |
| Age squared | 0.001^**^ | 0.001^*^ | 0.001^***^ | -0.001^***^ | -0.001^**^ | -0.001^*^ | -0.002^***^ | -0.002^***^ | -0.001^***^ |
|  | (0.000) | (0.000) | (0.000) | (0.000) | (0.000) | (0.000) | (0.000) | (0.000) | (0.000) |
| Women | 0.261^***^ | 0.649^***^ | 0.270^***^ | 0.410^***^ | 0.322^***^ | 0.417^***^ | 0.414^***^ | 0.232^***^ | 0.122^**^ |
|  | (0.045) | (0.032) | (0.031) | (0.049) | (0.040) | (0.045) | (0.075) | (0.044) | (0.037) |
| Medium education (ref. = low) | -0.332^***^ | -0.040 | -0.209^***^ | -0.029 | -0.074 | 0.163^*^ | 0.141 | 0.075 | 0.112^*^ |
|  | (0.057) | (0.045) | (0.042) | (0.066) | (0.056) | (0.066) | (0.094) | (0.056) | (0.051) |
| High education (ref. = low) | -0.654^***^ | 0.032 | -0.426^***^ | 0.192^*^ | 0.017 | 0.364^***^ | 0.470^***^ | 0.329^***^ | 0.190^***^ |
|  | (0.073) | (0.049) | (0.047) | (0.076) | (0.062) | (0.071) | (0.113) | (0.067) | (0.058) |
| Chronic disease dummy | 0.083 | 0.184^***^ | 0.245^***^ | 0.082 | 0.097^*^ | 0.237^***^ | 0.155 | -0.031 | 0.066 |
|  | (0.055) | (0.037) | (0.037) | (0.055) | (0.046) | (0.053) | (0.085) | (0.052) | (0.046) |
| ADL dummy | -0.053 | 0.023 | 0.206^*^ | 0.169 | 0.293^*^ | -0.075 | -0.273 | -0.010 | 0.004 |
|  | (0.104) | (0.086) | (0.081) | (0.134) | (0.117) | (0.119) | (0.179) | (0.101) | (0.089) |
| IADL dummy | 0.276^***^ | 0.149^*^ | 0.255^***^ | 0.012 | 0.062 | -0.039 | -0.146 | -0.256^**^ | -0.047 |
|  | (0.082) | (0.074) | (0.068) | (0.111) | (0.092) | (0.098) | (0.158) | (0.083) | (0.076) |
| Frailty dummy | 0.468^***^ | 0.227^*^ | 0.638^***^ | 0.643^***^ | 0.308^*^ | 0.280^*^ | -0.180 | -0.117 | -0.304^***^ |
|  | (0.084) | (0.096) | (0.088) | (0.171) | (0.127) | (0.134) | (0.184) | (0.099) | (0.086) |
| Cognition score = 1 (ref. = 0) | -0.209 | 0.245 | -0.481 | -0.125 | -0.533 | -0.002 | -1.508^*^ | -0.473 | -0.652 |
|  | (0.386) | (0.471) | (0.478) | (0.809) | (0.567) | (0.795) | (0.751) | (0.375) | (0.360) |
| Cognition score = 2 (ref. = 0) | -0.767^**^ | -0.146 | -0.616^*^ | -0.415 | -0.261 | -0.154 | -0.713 | 0.237 | -0.120 |
|  | (0.261) | (0.282) | (0.308) | (0.426) | (0.380) | (0.459) | (0.580) | (0.265) | (0.263) |
| Cognition score = 3 (ref. = 0) | -0.954^***^ | -0.202 | -0.728^**^ | -0.378 | -0.345 | 0.108 | -0.219 | 0.651^**^ | 0.116 |
|  | (0.221) | (0.246) | (0.271) | (0.362) | (0.330) | (0.401) | (0.524) | (0.222) | (0.226) |
| Cognition score = 4 (ref. = 0) | -1.064^***^ | -0.213 | -0.777^**^ | -0.386 | -0.429 | 0.210 | -0.151 | 0.681^**^ | 0.217 |
|  | (0.216) | (0.243) | (0.269) | (0.358) | (0.327) | (0.397) | (0.516) | (0.217) | (0.223) |
| Constant | -3.659^**^ | 0.607 | 2.051^*^ | -5.168^***^ | -3.403^**^ | -1.754 | -4.231^*^ | -4.710^***^ | -1.063 |
|  | (1.321) | (1.008) | (0.994) | (1.448) | (1.185) | (1.542) | (1.993) | (1.158) | (1.073) |
| Country dummies | Yes | Yes | Yes | Yes | Yes | Yes | Yes | Yes | Yes |
| Interview week dummies | Yes | Yes | Yes | Yes | Yes | Yes | Yes | Yes | Yes |
| N | 20,625 | 20,446 | 20,354 | 20,216 | 20,255 | 17,992 | 17,969 | 20,603 | 20,611 |
| Pseudo R2 | 0.184 | 0.093 | 0.212 | 0.080 | 0.061 | 0.524 | 0.046 | 0.036 | 0.069 |

Note: Logistic regressions based on the sample that was able to stand up during the chair stand test; the table presents coefficients along with standard errors in parentheses; * p<0.05, ** p<0.01, *** p<0.0

Table A.8: Robustness analysis for overestimating health: different specification of objective mobility

|  | (1) | (2) | (3) | (4) | (5) | (6) | (7) | (8) | (9) |
| --- | --- | --- | --- | --- | --- | --- | --- | --- | --- |
|  | Staying home | Less shopping | Less walks | Less meetings | Less visits | Wearing masks | Keeping distance | Washing hands | Sanitising hands |
| Overestimating | -0.027 | -0.012 | 0.006 | -0.137 | 0.135 | 0.312 | 0.600^*^ | -0.035 | 0.153 |
|  | (0.129) | (0.143) | (0.126) | (0.226) | (0.190) | (0.182) | (0.256) | (0.136) | (0.128) |
| Age | 0.039 | -0.048 | -0.082 | 0.136 | 0.264^*^ | 0.043 | 0.337^*^ | 0.084 | 0.092 |
|  | (0.088) | (0.099) | (0.083) | (0.135) | (0.110) | (0.132) | (0.144) | (0.086) | (0.079) |
| Age squared | 0.000 | 0.001 | 0.001 | -0.001 | -0.002^*^ | -0.001 | -0.003^**^ | -0.001 | -0.001 |
|  | (0.001) | (0.001) | (0.001) | (0.001) | (0.001) | (0.001) | (0.001) | (0.001) | (0.001) |
| Women | 0.498^***^ | 0.645^***^ | 0.409^***^ | 0.207 | 0.187 | 0.539^**^ | 0.499^*^ | 0.330^**^ | 0.167 |
|  | (0.124) | (0.126) | (0.111) | (0.204) | (0.168) | (0.168) | (0.229) | (0.126) | (0.119) |
| Medium education (ref. = low) | -0.336^*^ | -0.147 | 0.063 | -0.175 | 0.049 | -0.158 | 0.430 | -0.271 | 0.270^*^ |
|  | (0.137) | (0.146) | (0.128) | (0.233) | (0.201) | (0.196) | (0.257) | (0.150) | (0.137) |
| High education (ref. = low) | -0.543^**^ | 0.028 | -0.205 | 0.062 | -0.282 | 0.098 | 0.232 | -0.130 | 0.201 |
|  | (0.188) | (0.187) | (0.158) | (0.306) | (0.245) | (0.230) | (0.327) | (0.186) | (0.176) |
| Chronic disease dummy | -0.025 | 0.101 | 0.151 | 0.529^*^ | -0.019 | 0.342 | 0.018 | 0.155 | 0.174 |
|  | (0.167) | (0.173) | (0.154) | (0.238) | (0.245) | (0.218) | (0.316) | (0.180) | (0.168) |
| ADL dummy | 0.331^*^ | 0.436^*^ | 0.337^*^ | 0.503 | -0.003 | 0.334 | 0.553 | -0.131 | -0.166 |
|  | (0.144) | (0.192) | (0.163) | (0.320) | (0.246) | (0.238) | (0.311) | (0.145) | (0.143) |
| IADL dummy | 0.361^**^ | 0.379^*^ | 0.291^*^ | 0.166 | 0.248 | -0.215 | -0.117 | -0.265 | -0.117 |
|  | (0.136) | (0.179) | (0.147) | (0.284) | (0.247) | (0.212) | (0.273) | (0.147) | (0.143) |
| Frailty dummy | 0.200 | 0.282 | 0.518^***^ | 0.027 | 0.262 | -0.069 | -0.272 | -0.320^*^ | 0.074 |
|  | (0.134) | (0.168) | (0.140) | (0.255) | (0.214) | (0.202) | (0.259) | (0.140) | (0.132) |
| Cognition score = 1 (ref. = 0) | -0.311 | 0.742 | 0.224 | -0.115 | -0.705 | -0.563 | -0.928 | -0.092 | 0.366 |
|  | (0.593) | (1.227) | (0.879) | (1.148) | (1.296) | (1.137) | (1.361) | (0.635) | (0.618) |
| Cognition score = 2 (ref. = 0) | -0.312 | 0.631 | 0.573 | 0.428 | 0.259 | 0.649 | 0.000 | 0.069 | 0.200 |
|  | (0.469) | (0.743) | (0.645) | (0.740) | (1.035) | (0.999) | (.) | (0.447) | (0.463) |
| Cognition score = 3 (ref. = 0) | -1.146^**^ | 0.185 | -0.381 | 0.057 | -0.598 | 0.544 | 0.537 | 0.412 | 0.149 |
|  | (0.399) | (0.577) | (0.510) | (0.260) | (0.782) | (0.810) | (0.749) | (0.383) | (0.395) |
| Cognition score = 4 (ref. = 0) | -1.371^***^ | -0.197 | -0.474 | 0.000 | -0.395 | 0.582 | 0.989 | 0.660 | 0.419 |
|  | (0.386) | (0.556) | (0.498) | (.) | (0.768) | (0.786) | (0.713) | (0.365) | (0.382) |
| Constant | -5.564 | 0.606 | 0.498 | -3.870 | -7.805^*^ | 0.759 | -8.780 | -1.038 | -1.594 |
|  | (3.040) | (3.256) | (2.802) | (4.425) | (3.702) | (4.407) | (4.991) | (2.973) | (2.716) |
| Country dummies | Yes | Yes | Yes | Yes | Yes | Yes | Yes | Yes | Yes |
| Interview week dummies | Yes | Yes | Yes | Yes | Yes | Yes | Yes | Yes | Yes |
| N | 2,145 | 2,104 | 2,112 | 2,010 | 2,078 | 1,489 | 1,386 | 2,135 | 2,140 |
| Pseudo R2 | 0.246 | 0.150 | 0.216 | 0.104 | 0.127 | 0.443 | 0.129 | 0.059 | 0.066 |

Note: Logistic regressions based on the sample that was unable to stand up during the chair stand test; the table presents coefficients along with standard errors in parentheses; * p<0.05, ** p<0.01, *** p<0.0

Table A.9: Robustness analysis for underestimating health: additional control variables

|  | (1) | (2) | (3) | (4) | (5) | (6) | (7) | (8) | (9) |
| --- | --- | --- | --- | --- | --- | --- | --- | --- | --- |
|  | Staying home | Less shopping | Less walks | Less meetings | Less visits | Wearing masks | Keeping distance | Washing hands | Sanitising hands |
| Underestimating | 0.246^***^ | 0.155^**^ | 0.188^***^ | -0.050 | -0.059 | 0.069 | 0.094 | 0.042 | -0.008 |
|  | (0.067) | (0.058) | (0.053) | (0.086) | (0.070) | (0.083) | (0.131) | (0.072) | (0.062) |
| Age | 0.005 | -0.045 | -0.103^**^ | 0.154^***^ | 0.090^*^ | 0.085 | 0.211^**^ | 0.200^***^ | 0.090^*^ |
|  | (0.042) | (0.032) | (0.032) | (0.046) | (0.038) | (0.048) | (0.066) | (0.039) | (0.035) |
| Age squared | 0.001 | 0.001^*^ | 0.001^***^ | -0.001^**^ | -0.001^*^ | -0.001 | -0.002^***^ | -0.002^***^ | -0.001^***^ |
|  | (0.000) | (0.000) | (0.000) | (0.000) | (0.000) | (0.000) | (0.000) | (0.000) | (0.000) |
| Women | 0.229^***^ | 0.653^***^ | 0.211^***^ | 0.403^***^ | 0.329^***^ | 0.409^***^ | 0.455^***^ | 0.250^***^ | 0.142^***^ |
|  | (0.047) | (0.033) | (0.032) | (0.050) | (0.041) | (0.046) | (0.078) | (0.046) | (0.039) |
| Medium education (ref. = low) | -0.317^***^ | -0.039 | -0.194^***^ | -0.034 | -0.071 | 0.161^*^ | 0.115 | 0.067 | 0.112^*^ |
|  | (0.058) | (0.045) | (0.042) | (0.067) | (0.057) | (0.067) | (0.095) | (0.057) | (0.051) |
| High education (ref. = low) | -0.649^***^ | 0.028 | -0.424^***^ | 0.186^*^ | 0.020 | 0.367^***^ | 0.431^***^ | 0.315^***^ | 0.188^**^ |
|  | (0.074) | (0.050) | (0.048) | (0.077) | (0.063) | (0.071) | (0.114) | (0.068) | (0.058) |
| Chronic disease dummy | 0.065 | 0.172^***^ | 0.216^***^ | 0.065 | 0.077 | 0.222^***^ | 0.155 | -0.025 | 0.065 |
|  | (0.055) | (0.037) | (0.037) | (0.056) | (0.046) | (0.053) | (0.086) | (0.052) | (0.047) |
| ADL dummy | -0.039 | 0.028 | 0.199^*^ | 0.162 | 0.293^*^ | -0.100 | -0.242 | -0.039 | 0.026 |
|  | (0.106) | (0.089) | (0.083) | (0.138) | (0.119) | (0.122) | (0.185) | (0.103) | (0.092) |
| IADL dummy | 0.301^***^ | 0.134 | 0.244^***^ | 0.011 | 0.035 | -0.045 | -0.206 | -0.270^**^ | -0.051 |
|  | (0.083) | (0.075) | (0.069) | (0.114) | (0.094) | (0.100) | (0.158) | (0.085) | (0.078) |
| Frailty dummy | 0.428^***^ | 0.208^*^ | 0.593^***^ | 0.589^***^ | 0.275^*^ | 0.236 | -0.183 | -0.078 | -0.282^**^ |
|  | (0.086) | (0.098) | (0.091) | (0.174) | (0.129) | (0.137) | (0.187) | (0.104) | (0.089) |
| Cognition score = 1 (ref. = 0) | -0.105 | 0.281 | -0.482 | -0.193 | -0.550 | -0.139 | -1.509^*^ | -0.413 | -0.658 |
|  | (0.396) | (0.471) | (0.487) | (0.821) | (0.571) | (0.809) | (0.756) | (0.396) | (0.378) |
| Cognition score = 2 (ref. = 0) | -0.663^*^ | -0.065 | -0.566 | -0.372 | -0.112 | -0.126 | -0.645 | 0.251 | -0.172 |
|  | (0.275) | (0.287) | (0.316) | (0.425) | (0.383) | (0.461) | (0.579) | (0.275) | (0.275) |
| Cognition score = 3 (ref. = 0) | -0.824^***^ | -0.113 | -0.694^*^ | -0.324 | -0.256 | 0.165 | -0.137 | 0.654^**^ | 0.074 |
|  | (0.230) | (0.250) | (0.278) | (0.360) | (0.329) | (0.401) | (0.522) | (0.230) | (0.237) |
| Cognition score = 4 (ref. = 0) | -0.917^***^ | -0.119 | -0.728^**^ | -0.318 | -0.334 | 0.252 | -0.062 | 0.677^**^ | 0.160 |
|  | (0.226) | (0.247) | (0.275) | (0.355) | (0.326) | (0.397) | (0.514) | (0.225) | (0.234) |
| Retired | -0.116 | -0.016 | -0.024 | 0.160^*^ | 0.127^*^ | 0.122 | 0.050 | -0.071 | -0.052 |
|  | (0.065) | (0.050) | (0.048) | (0.076) | (0.062) | (0.072) | (0.119) | (0.069) | (0.060) |
| Living with partner | 0.065 | 0.352^***^ | 0.025 | 0.265^***^ | 0.323^***^ | 0.178^**^ | 0.303^***^ | 0.264^***^ | 0.292^***^ |
|  | (0.055) | (0.039) | (0.039) | (0.057) | (0.047) | (0.056) | (0.086) | (0.051) | (0.046) |
| Feeling depressed or sad | 0.261^***^ | 0.303^***^ | 0.474^***^ | 0.304^***^ | 0.296^***^ | 0.292^***^ | 0.147 | 0.129^*^ | 0.082 |
|  | (0.053) | (0.045) | (0.041) | (0.069) | (0.057) | (0.062) | (0.101) | (0.057) | (0.049) |
| Anyone tested positive | -0.064 | 0.119^*^ | -0.070 | 0.132 | 0.106 | 0.001 | 0.266 | 0.188^*^ | 0.060 |
|  | (0.090) | (0.059) | (0.059) | (0.096) | (0.076) | (0.081) | (0.156) | (0.088) | (0.074) |
| Constant | -4.905^***^ | 0.153 | 1.605 | -3.791^*^ | -2.320 | -0.897 | -3.522 | -5.206^***^ | -1.431 |
|  | (1.460) | (1.090) | (1.087) | (1.556) | (1.282) | (1.649) | (2.295) | (1.322) | (1.200) |
| Country dummies | Yes | Yes | Yes | Yes | Yes | Yes | Yes | Yes | Yes |
| Interview week dummies | Yes | Yes | Yes | Yes | Yes | Yes | Yes | Yes | Yes |
| N | 20,280 | 20,108 | 20,020 | 19,882 | 19,921 | 17,720 | 17,705 | 20,265 | 20,273 |
| Pseudo R2 | 0.187 | 0.098 | 0.216 | 0.084 | 0.065 | 0.527 | 0.050 | 0.038 | 0.071 |

Note: Logistic regressions based on the sample that was able to stand up during the chair stand test; the table presents coefficients along with standard errors in parentheses; * p<0.05, ** p<0.01, *** p<0.0

Table A.10: Robustness analysis for overestimating health: additional control variables

|  | (1) | (2) | (3) | (4) | (5) | (6) | (7) | (8) | (9) |
| --- | --- | --- | --- | --- | --- | --- | --- | --- | --- |
|  | Staying home | Less shopping | Less walks | Less meetings | Less visits | Wearing masks | Keeping distance | Washing hands | Sanitising hands |
| Overestimating | 0.007 | 0.025 | 0.009 | -0.068 | 0.240 | 0.322 | 0.712^**^ | 0.012 | 0.152 |
|  | (0.128) | (0.143) | (0.126) | (0.221) | (0.187) | (0.180) | (0.254) | (0.136) | (0.128) |
| Age | 0.050 | -0.071 | -0.080 | -0.023 | 0.267^*^ | 0.184 | 0.434^**^ | 0.192^*^ | 0.107 |
|  | (0.094) | (0.108) | (0.090) | (0.143) | (0.123) | (0.138) | (0.164) | (0.094) | (0.088) |
| Age squared | 0.000 | 0.001 | 0.001 | 0.000 | -0.002^*^ | -0.001 | -0.003^**^ | -0.002^*^ | -0.001 |
|  | (0.001) | (0.001) | (0.001) | (0.001) | (0.001) | (0.001) | (0.001) | (0.001) | (0.001) |
| Women | 0.446^***^ | 0.716^***^ | 0.380^***^ | 0.284 | 0.102 | 0.597^***^ | 0.660^**^ | 0.393^**^ | 0.206 |
|  | (0.128) | (0.128) | (0.113) | (0.208) | (0.168) | (0.167) | (0.228) | (0.132) | (0.123) |
| Medium education (ref. = low) | -0.387^**^ | -0.093 | 0.133 | -0.054 | 0.097 | -0.203 | 0.392 | -0.255 | 0.260 |
|  | (0.138) | (0.148) | (0.128) | (0.230) | (0.201) | (0.196) | (0.246) | (0.151) | (0.137) |
| High education (ref. = low) | -0.597^**^ | 0.068 | -0.140 | 0.188 | -0.236 | 0.031 | 0.155 | -0.106 | 0.186 |
|  | (0.192) | (0.188) | (0.156) | (0.301) | (0.244) | (0.227) | (0.326) | (0.189) | (0.176) |
| Chronic disease dummy | 0.019 | 0.117 | 0.146 | 0.475 | -0.064 | 0.342 | 0.188 | 0.087 | 0.202 |
|  | (0.171) | (0.177) | (0.157) | (0.243) | (0.251) | (0.220) | (0.307) | (0.179) | (0.168) |
| ADL dummy | 0.369^*^ | 0.372^*^ | 0.256 | 0.454 | -0.064 | 0.302 | 0.547 | -0.121 | -0.182 |
|  | (0.144) | (0.189) | (0.161) | (0.303) | (0.241) | (0.233) | (0.310) | (0.149) | (0.144) |
| IADL dummy | 0.323^*^ | 0.360^*^ | 0.254 | 0.087 | 0.261 | -0.196 | -0.131 | -0.380^*^ | -0.106 |
|  | (0.136) | (0.175) | (0.147) | (0.282) | (0.247) | (0.207) | (0.265) | (0.148) | (0.142) |
| Frailty dummy | 0.185 | 0.307 | 0.508^***^ | 0.107 | 0.271 | -0.082 | -0.046 | -0.258 | 0.059 |
|  | (0.134) | (0.168) | (0.142) | (0.255) | (0.213) | (0.203) | (0.262) | (0.140) | (0.133) |
| Cognition score = 1 (ref. = 0) | -0.486 | 0.830 | 0.463 | -0.068 | -0.623 | -0.510 | -1.027 | -0.744 | 0.122 |
|  | (0.593) | (1.259) | (0.874) | (1.128) | (1.297) | (1.197) | (1.303) | (0.662) | (0.632) |
| Cognition score = 2 (ref. = 0) | -0.319 | 0.881 | 0.657 | 0.499 | 0.129 | 0.780 | 0.000 | -0.161 | 0.036 |
|  | (0.460) | (0.760) | (0.647) | (0.734) | (0.943) | (0.971) | (.) | (0.489) | (0.478) |
| Cognition score = 3 (ref. = 0) | -1.005^*^ | 0.286 | -0.172 | 0.031 | -0.318 | 0.447 | 0.547 | 0.153 | -0.031 |
|  | (0.397) | (0.600) | (0.522) | (0.257) | (0.765) | (0.810) | (0.700) | (0.430) | (0.415) |
| Cognition score = 4 (ref. = 0) | -1.229^**^ | -0.017 | -0.249 | 0.000 | -0.267 | 0.595 | 1.075 | 0.376 | 0.187 |
|  | (0.385) | (0.578) | (0.509) | (.) | (0.748) | (0.789) | (0.662) | (0.414) | (0.402) |
| Retired | -0.013 | -0.060 | 0.091 | 0.470 | -0.098 | -0.430 | -0.303 | -0.190 | 0.001 |
|  | (0.148) | (0.172) | (0.154) | (0.263) | (0.230) | (0.222) | (0.325) | (0.163) | (0.150) |
| Living with partner | 0.004 | 0.383^**^ | -0.069 | 0.503^*^ | 0.291 | 0.358^*^ | 0.655^**^ | -0.003 | 0.258^*^ |
|  | (0.122) | (0.133) | (0.115) | (0.204) | (0.175) | (0.166) | (0.223) | (0.132) | (0.120) |
| Feeling depressed or sad | 0.339^**^ | 0.609^***^ | 0.472^***^ | 0.563^*^ | 0.386^*^ | 0.301 | -0.032 | 0.230 | 0.005 |
|  | (0.121) | (0.151) | (0.119) | (0.237) | (0.190) | (0.186) | (0.246) | (0.133) | (0.121) |
| Anyone tested positive | -0.368 | 0.006 | -0.355 | -0.108 | -0.006 | 0.230 | 0.835 | 0.272 | 0.417 |
|  | (0.258) | (0.236) | (0.199) | (0.394) | (0.331) | (0.343) | (0.721) | (0.270) | (0.248) |
| Constant | -6.197 | 0.535 | 0.178 | 0.720 | -8.404^*^ | -4.566 | -13.044^*^ | -4.539 | -2.049 |
|  | (3.222) | (3.570) | (3.010) | (4.766) | (4.154) | (4.655) | (5.690) | (3.207) | (3.000) |
| Country dummies | Yes | Yes | Yes | Yes | Yes | Yes | Yes | Yes | Yes |
| Interview week dummies | Yes | Yes | Yes | Yes | Yes | Yes | Yes | Yes | Yes |
| N | 2,190 | 2,149 | 2,154 | 2,054 | 2,124 | 1,548 | 1,436 | 2,184 | 2,189 |
| Pseudo R2 | 0.238 | 0.165 | 0.223 | 0.111 | 0.129 | 0.447 | 0.142 | 0.067 | 0.075 |

Note: Logistic regressions based on the sample that was unable to stand up during the chair stand test; the table presents coefficients along with standard errors in parentheses; * p<0.05, ** p<0.01, *** p<0.0

Table A.11: Robustness analysis for underestimating health: additional control variable income

|  | (1) | (2) | (3) | (4) | (5) | (6) | (7) | (8) | (9) |
| --- | --- | --- | --- | --- | --- | --- | --- | --- | --- |
|  | Staying home | Less shopping | Less walks | Less meetings | Less visits | Wearing masks | Keeping distance | Washing hands | Sanitising hands |
| Underestimating | 0.205^**^ | 0.136^*^ | 0.201^***^ | -0.096 | -0.025 | 0.128 | 0.130 | 0.108 | 0.038 |
|  | (0.078) | (0.064) | (0.059) | (0.093) | (0.078) | (0.091) | (0.148) | (0.083) | (0.070) |
| Age | -0.045 | -0.071^*^ | -0.103^**^ | 0.201^***^ | 0.136^**^ | 0.117^*^ | 0.211^**^ | 0.215^***^ | 0.098^**^ |
|  | (0.046) | (0.035) | (0.035) | (0.051) | (0.041) | (0.053) | (0.068) | (0.041) | (0.037) |
| Age squared | 0.001^*^ | 0.001^**^ | 0.001^***^ | -0.001^***^ | -0.001^**^ | -0.001^*^ | -0.002^***^ | -0.002^***^ | -0.001^***^ |
|  | (0.000) | (0.000) | (0.000) | (0.000) | (0.000) | (0.000) | (0.001) | (0.000) | (0.000) |
| Women | 0.255^***^ | 0.691^***^ | 0.262^***^ | 0.412^***^ | 0.329^***^ | 0.405^***^ | 0.500^***^ | 0.251^***^ | 0.146^***^ |
|  | (0.053) | (0.036) | (0.036) | (0.056) | (0.044) | (0.050) | (0.087) | (0.051) | (0.043) |
| Medium education (ref. = low) | -0.297^***^ | 0.000 | -0.176^***^ | 0.011 | -0.077 | 0.129 | 0.068 | 0.013 | 0.059 |
|  | (0.066) | (0.052) | (0.048) | (0.075) | (0.064) | (0.074) | (0.108) | (0.065) | (0.058) |
| High education (ref. = low) | -0.575^***^ | 0.062 | -0.344^***^ | 0.196^*^ | 0.025 | 0.271^***^ | 0.206 | 0.203^**^ | 0.095 |
|  | (0.088) | (0.057) | (0.056) | (0.087) | (0.071) | (0.082) | (0.128) | (0.078) | (0.067) |
| Chronic disease dummy | 0.106 | 0.177^***^ | 0.236^***^ | 0.097 | 0.098 | 0.211^***^ | 0.101 | -0.045 | 0.072 |
|  | (0.065) | (0.042) | (0.042) | (0.062) | (0.051) | (0.060) | (0.099) | (0.060) | (0.053) |
| ADL dummy | -0.091 | 0.023 | 0.146 | 0.188 | 0.308^*^ | -0.095 | -0.182 | -0.043 | 0.031 |
|  | (0.120) | (0.097) | (0.090) | (0.151) | (0.130) | (0.131) | (0.208) | (0.114) | (0.101) |
| IADL dummy | 0.291^**^ | 0.115 | 0.214^**^ | -0.048 | -0.007 | -0.034 | -0.217 | -0.241^**^ | -0.050 |
|  | (0.094) | (0.082) | (0.077) | (0.123) | (0.101) | (0.108) | (0.171) | (0.093) | (0.087) |
| Frailty dummy | 0.528^***^ | 0.206 | 0.643^***^ | 0.586^**^ | 0.214 | 0.297^*^ | -0.125 | -0.180 | -0.357^***^ |
|  | (0.097) | (0.109) | (0.100) | (0.191) | (0.139) | (0.149) | (0.212) | (0.113) | (0.098) |
| Cognition score = 1 (ref. = 0) | -0.074 | -0.123 | -0.560 | -0.699 | -0.903 | -0.761 | -2.031^*^ | -0.566 | -0.536 |
|  | (0.461) | (0.525) | (0.550) | (0.857) | (0.617) | (0.970) | (0.946) | (0.470) | (0.449) |
| Cognition score = 2 (ref. = 0) | -0.803^*^ | -0.360 | -0.855^*^ | -0.675 | -0.223 | -0.250 | -1.124 | 0.219 | -0.100 |
|  | (0.336) | (0.342) | (0.356) | (0.506) | (0.450) | (0.552) | (0.779) | (0.341) | (0.328) |
| Cognition score = 3 (ref. = 0) | -1.066^***^ | -0.453 | -0.980^**^ | -0.579 | -0.381 | 0.060 | -0.523 | 0.633^*^ | 0.149 |
|  | (0.286) | (0.299) | (0.314) | (0.433) | (0.390) | (0.488) | (0.723) | (0.289) | (0.284) |
| Cognition score = 4 (ref. = 0) | -1.149^***^ | -0.443 | -0.988^**^ | -0.540 | -0.446 | 0.186 | -0.479 | 0.647^*^ | 0.232 |
|  | (0.280) | (0.296) | (0.311) | (0.429) | (0.386) | (0.483) | (0.715) | (0.283) | (0.281) |
| Lowest income | -0.088^***^ | 0.010 | -0.044^***^ | -0.002 | -0.000 | 0.063^***^ | 0.114^***^ | 0.049^***^ | 0.063^***^ |
|  | (0.015) | (0.010) | (0.011) | (0.014) | (0.011) | (0.015) | (0.019) | (0.013) | (0.012) |
| Constant | -1.880 | 1.560 | 2.552^*^ | -4.841^**^ | -3.363^*^ | -2.524 | -4.103 | -5.979^***^ | -2.239 |
|  | (1.584) | (1.181) | (1.173) | (1.713) | (1.389) | (1.810) | (2.377) | (1.384) | (1.254) |
| Country dummies | Yes | Yes | Yes | Yes | Yes | Yes | Yes | Yes | Yes |
| Interview week dummies | Yes | Yes | Yes | Yes | Yes | Yes | Yes | Yes | Yes |
| N | 15,768 | 15,652 | 15,577 | 15,500 | 15,513 | 13,877 | 13,864 | 15,755 | 15,762 |
| Pseudo R2 | 0.196 | 0.090 | 0.203 | 0.081 | 0.058 | 0.515 | 0.050 | 0.035 | 0.068 |

Note: Logistic regressions based on the sample that was able to stand up during the chair stand test; the table presents coefficients along with standard errors in parentheses; * p<0.05, ** p<0.01, *** p<0.0

Table A.12: Robustness analysis for overestimating health: additional control variable income

|  | (1) | (2) | (3) | (4) | (5) | (6) | (7) | (8) | (9) |
| --- | --- | --- | --- | --- | --- | --- | --- | --- | --- |
|  | Staying home | Less shopping | Less walks | Less meetings | Less visits | Wearing masks | Keeping distance | Washing hands | Sanitising hands |
| Overestimating | 0.104 | 0.044 | -0.091 | -0.108 | 0.274 | 0.172 | 0.679^*^ | -0.027 | 0.058 |
|  | (0.151) | (0.159) | (0.142) | (0.246) | (0.209) | (0.198) | (0.282) | (0.155) | (0.143) |
| Age | 0.150 | -0.043 | -0.045 | 0.059 | 0.274^*^ | -0.035 | 0.372^*^ | 0.078 | 0.058 |
|  | (0.111) | (0.115) | (0.095) | (0.164) | (0.123) | (0.141) | (0.164) | (0.104) | (0.095) |
| Age squared | -0.000 | 0.001 | 0.001 | -0.000 | -0.002^*^ | 0.000 | -0.003^*^ | -0.001 | -0.001 |
|  | (0.001) | (0.001) | (0.001) | (0.001) | (0.001) | (0.001) | (0.001) | (0.001) | (0.001) |
| Women | 0.463^**^ | 0.707^***^ | 0.496^***^ | 0.358 | 0.217 | 0.526^**^ | 0.596^*^ | 0.369^*^ | 0.176 |
|  | (0.141) | (0.139) | (0.126) | (0.219) | (0.185) | (0.189) | (0.249) | (0.144) | (0.135) |
| Medium education (ref. = low) | -0.344^*^ | -0.044 | 0.045 | -0.016 | -0.061 | -0.074 | 0.455 | -0.362^*^ | 0.121 |
|  | (0.163) | (0.164) | (0.144) | (0.260) | (0.227) | (0.215) | (0.279) | (0.173) | (0.156) |
| High education (ref. = low) | -0.523^*^ | 0.064 | 0.042 | 0.281 | -0.381 | 0.062 | 0.082 | -0.122 | 0.052 |
|  | (0.223) | (0.214) | (0.182) | (0.355) | (0.281) | (0.264) | (0.382) | (0.223) | (0.209) |
| Chronic disease dummy | -0.157 | 0.289 | 0.113 | 0.573^*^ | 0.032 | 0.355 | -0.093 | 0.223 | 0.139 |
|  | (0.193) | (0.192) | (0.179) | (0.279) | (0.284) | (0.243) | (0.351) | (0.199) | (0.188) |
| ADL dummy | 0.491^**^ | 0.433^*^ | 0.415^*^ | 0.472 | 0.045 | 0.220 | 0.270 | -0.148 | -0.167 |
|  | (0.165) | (0.208) | (0.181) | (0.343) | (0.271) | (0.259) | (0.335) | (0.167) | (0.165) |
| IADL dummy | 0.437^**^ | 0.367 | 0.282 | 0.217 | 0.170 | -0.161 | 0.006 | -0.276 | -0.196 |
|  | (0.163) | (0.198) | (0.166) | (0.315) | (0.287) | (0.242) | (0.307) | (0.174) | (0.166) |
| Frailty dummy | 0.407^**^ | 0.323 | 0.473^**^ | 0.013 | 0.264 | 0.004 | -0.164 | -0.297 | 0.068 |
|  | (0.154) | (0.185) | (0.157) | (0.286) | (0.239) | (0.222) | (0.286) | (0.161) | (0.153) |
| Cognition score = 1 (ref. = 0) | -0.300 | 0.000 | 0.000 | 0.000 | 0.000 | 0.000 | 0.000 | -0.746 | 0.444 |
|  | (0.826) | (.) | (.) | (.) | (.) | (.) | (.) | (0.862) | (0.850) |
| Cognition score = 2 (ref. = 0) | -0.313 | 1.016 | 0.720 | 0.981 | 0.151 | -0.194 | 0.000 | -0.133 | 0.494 |
|  | (0.618) | (0.842) | (0.729) | (0.990) | (0.963) | (1.129) | (.) | (0.589) | (0.585) |
| Cognition score = 3 (ref. = 0) | -1.283^*^ | 0.289 | -0.087 | -0.178 | -0.354 | 0.172 | 0.383 | 0.242 | 0.490 |
|  | (0.530) | (0.650) | (0.595) | (0.278) | (0.777) | (0.915) | (0.958) | (0.516) | (0.499) |
| Cognition score = 4 (ref. = 0) | -1.459^**^ | -0.024 | -0.232 | 0.000 | -0.114 | 0.307 | 0.799 | 0.503 | 0.633 |
|  | (0.515) | (0.627) | (0.580) | (.) | (0.757) | (0.890) | (0.917) | (0.497) | (0.483) |
| Lowest income | -0.075 | 0.023 | -0.055 | 0.079 | 0.094 | 0.037 | 0.079 | 0.055 | 0.115^**^ |
|  | (0.045) | (0.044) | (0.036) | (0.049) | (0.058) | (0.054) | (0.082) | (0.040) | (0.040) |
| Constant | -8.515^*^ | -0.260 | -0.299 | -3.094 | -9.559^*^ | 2.968 | -10.529 | -1.459 | -1.758 |
|  | (3.915) | (3.809) | (3.219) | (5.279) | (4.233) | (4.765) | (5.665) | (3.592) | (3.274) |
| Country dummies | Yes | Yes | Yes | Yes | Yes | Yes | Yes | Yes | Yes |
| Interview week dummies | Yes | Yes | Yes | Yes | Yes | Yes | Yes | Yes | Yes |
| N | 1,658 | 1,620 | 1,624 | 1,569 | 1,596 | 1,129 | 1,106 | 1,655 | 1,655 |
| Pseudo R2 | 0.270 | 0.151 | 0.210 | 0.123 | 0.122 | 0.396 | 0.131 | 0.063 | 0.065 |

Note: Logistic regressions based on the sample that was unable to stand up during the chair stand test; the table presents coefficients along with standard errors in parentheses; * p<0.05, ** p<0.01, *** p<0.0

Table A.13: Robustness analysis for underestimating health: interaction between country and interview week dummies

|  | (1) | (2) | (3) | (4) | (5) | (6) | (7) | (8) | (9) |
| --- | --- | --- | --- | --- | --- | --- | --- | --- | --- |
|  | Staying home | Less shopping | Less walks | Less meetings | Less visits | Wearing masks | Keeping distance | Washing hands | Sanitising hands |
| Underestimating | 0.250^***^ | 0.164^**^ | 0.192^***^ | -0.058 | -0.043 | 0.070 | 0.114 | 0.040 | -0.018 |
|  | (0.066) | (0.057) | (0.052) | (0.085) | (0.070) | (0.082) | (0.132) | (0.072) | (0.061) |
| Age | -0.031 | -0.044 | -0.115^***^ | 0.210^***^ | 0.137^***^ | 0.109^*^ | 0.252^***^ | 0.196^***^ | 0.087^**^ |
|  | (0.039) | (0.031) | (0.030) | (0.044) | (0.036) | (0.046) | (0.059) | (0.035) | (0.032) |
| Age squared | 0.001^**^ | 0.001^*^ | 0.001^***^ | -0.001^***^ | -0.001^***^ | -0.001^*^ | -0.002^***^ | -0.002^***^ | -0.001^***^ |
|  | (0.000) | (0.000) | (0.000) | (0.000) | (0.000) | (0.000) | (0.000) | (0.000) | (0.000) |
| Women | 0.263^***^ | 0.646^***^ | 0.272^***^ | 0.411^***^ | 0.329^***^ | 0.419^***^ | 0.418^***^ | 0.228^***^ | 0.120^**^ |
|  | (0.045) | (0.032) | (0.032) | (0.050) | (0.040) | (0.046) | (0.076) | (0.044) | (0.038) |
| Medium education (ref. = low) | -0.323^***^ | -0.039 | -0.214^***^ | -0.041 | -0.073 | 0.172^**^ | 0.119 | 0.089 | 0.119^*^ |
|  | (0.057) | (0.045) | (0.042) | (0.067) | (0.057) | (0.066) | (0.095) | (0.057) | (0.051) |
| High education (ref. = low) | -0.638^***^ | 0.030 | -0.427^***^ | 0.190^*^ | 0.018 | 0.370^***^ | 0.466^***^ | 0.341^***^ | 0.193^***^ |
|  | (0.074) | (0.049) | (0.048) | (0.076) | (0.063) | (0.071) | (0.114) | (0.067) | (0.058) |
| Chronic disease dummy | 0.090 | 0.182^***^ | 0.241^***^ | 0.078 | 0.094^*^ | 0.245^***^ | 0.146 | -0.032 | 0.068 |
|  | (0.055) | (0.037) | (0.037) | (0.055) | (0.046) | (0.053) | (0.086) | (0.052) | (0.046) |
| ADL dummy | -0.074 | 0.029 | 0.214^**^ | 0.188 | 0.311^**^ | -0.069 | -0.284 | -0.012 | 0.016 |
|  | (0.105) | (0.087) | (0.081) | (0.137) | (0.118) | (0.120) | (0.182) | (0.102) | (0.090) |
| IADL dummy | 0.288^***^ | 0.136 | 0.246^***^ | 0.010 | 0.058 | -0.057 | -0.127 | -0.238^**^ | -0.055 |
|  | (0.083) | (0.074) | (0.069) | (0.113) | (0.092) | (0.099) | (0.160) | (0.084) | (0.077) |
| Frailty dummy | 0.489^***^ | 0.234^*^ | 0.663^***^ | 0.653^***^ | 0.309^*^ | 0.283^*^ | -0.230 | -0.124 | -0.310^***^ |
|  | (0.086) | (0.097) | (0.089) | (0.173) | (0.128) | (0.136) | (0.185) | (0.101) | (0.087) |
| Cognition score = 1 (ref. = 0) | -0.132 | 0.308 | -0.485 | -0.146 | -0.513 | 0.076 | -1.367 | -0.418 | -0.647 |
|  | (0.391) | (0.468) | (0.478) | (0.805) | (0.572) | (0.807) | (0.743) | (0.381) | (0.363) |
| Cognition score = 2 (ref. = 0) | -0.761^**^ | -0.162 | -0.621^*^ | -0.469 | -0.232 | -0.133 | -0.633 | 0.210 | -0.145 |
|  | (0.271) | (0.284) | (0.311) | (0.421) | (0.384) | (0.467) | (0.579) | (0.271) | (0.268) |
| Cognition score = 3 (ref. = 0) | -0.916^***^ | -0.178 | -0.717^**^ | -0.374 | -0.318 | 0.176 | -0.076 | 0.650^**^ | 0.136 |
|  | (0.226) | (0.247) | (0.274) | (0.355) | (0.332) | (0.409) | (0.521) | (0.227) | (0.229) |
| Cognition score = 4 (ref. = 0) | -1.021^***^ | -0.191 | -0.761^**^ | -0.390 | -0.404 | 0.258 | -0.036 | 0.676^**^ | 0.237 |
|  | (0.221) | (0.244) | (0.272) | (0.351) | (0.329) | (0.404) | (0.512) | (0.222) | (0.226) |
| Constant | -3.351^*^ | 0.480 | 2.166^*^ | -5.375^***^ | -3.559^**^ | -1.706 | -4.643^*^ | -4.750^***^ | -1.067 |
|  | (1.340) | (1.015) | (1.005) | (1.463) | (1.201) | (1.560) | (2.032) | (1.182) | (1.093) |
| Country dummies | Yes | Yes | Yes | Yes | Yes | Yes | Yes | Yes | Yes |
| Interview week dummies | Yes | Yes | Yes | Yes | Yes | Yes | Yes | Yes | Yes |
| Interaction between dummies | Yes | Yes | Yes | Yes | Yes | Yes | Yes | Yes | Yes |
| N | 20,276 | 20,299 | 20,217 | 19,903 | 20,021 | 17,234 | 17,293 | 20,458 | 20,427 |
| Pseudo R2 | 0.190 | 0.097 | 0.214 | 0.087 | 0.065 | 0.513 | 0.056 | 0.041 | 0.074 |

Note: Logistic regressions based on the sample that was able to stand up during the chair stand test; the table presents coefficients along with standard errors in parentheses; * p<0.05, ** p<0.01, *** p<0.0

Table A.14: Robustness analysis for overestimating health: interaction between country and interview week dummies

|  | (1) | (2) | (3) | (4) | (5) | (6) | (7) | (8) | (9) |
| --- | --- | --- | --- | --- | --- | --- | --- | --- | --- |
|  | Staying home | Less shopping | Less walks | Less meetings | Less visits | Wearing masks | Keeping distance | Washing hands | Sanitising hands |
| Overestimating | -0.012 | -0.024 | 0.011 | -0.148 | 0.181 | 0.337 | 0.701^**^ | -0.007 | 0.115 |
|  | (0.130) | (0.145) | (0.129) | (0.225) | (0.186) | (0.186) | (0.264) | (0.137) | (0.128) |
| Age | 0.030 | -0.051 | -0.087 | 0.114 | 0.232^*^ | 0.083 | 0.435^**^ | 0.136 | 0.110 |
|  | (0.088) | (0.102) | (0.084) | (0.131) | (0.111) | (0.137) | (0.154) | (0.089) | (0.083) |
| Age squared | 0.000 | 0.001 | 0.001 | -0.001 | -0.001 | -0.001 | -0.003^**^ | -0.001^*^ | -0.001 |
|  | (0.001) | (0.001) | (0.001) | (0.001) | (0.001) | (0.001) | (0.001) | (0.001) | (0.001) |
| Women | 0.498^***^ | 0.752^***^ | 0.421^***^ | 0.299 | 0.177 | 0.618^***^ | 0.629^**^ | 0.381^**^ | 0.195 |
|  | (0.124) | (0.127) | (0.114) | (0.202) | (0.171) | (0.177) | (0.234) | (0.127) | (0.119) |
| Medium education (ref. = low) | -0.380^**^ | -0.049 | 0.090 | -0.080 | 0.066 | -0.185 | 0.503^*^ | -0.336^*^ | 0.223 |
|  | (0.139) | (0.149) | (0.130) | (0.229) | (0.205) | (0.202) | (0.256) | (0.154) | (0.138) |
| High education (ref. = low) | -0.603^**^ | 0.144 | -0.161 | 0.192 | -0.218 | 0.047 | 0.313 | -0.186 | 0.198 |
|  | (0.188) | (0.190) | (0.161) | (0.305) | (0.245) | (0.235) | (0.321) | (0.193) | (0.177) |
| Chronic disease dummy | 0.035 | 0.202 | 0.236 | 0.612^*^ | 0.060 | 0.331 | 0.115 | 0.117 | 0.132 |
|  | (0.171) | (0.181) | (0.159) | (0.247) | (0.243) | (0.223) | (0.305) | (0.185) | (0.169) |
| ADL dummy | 0.363^*^ | 0.413^*^ | 0.315 | 0.476 | -0.005 | 0.375 | 0.583 | -0.086 | -0.178 |
|  | (0.144) | (0.194) | (0.164) | (0.311) | (0.244) | (0.239) | (0.317) | (0.146) | (0.147) |
| IADL dummy | 0.310^*^ | 0.479^**^ | 0.317^*^ | 0.138 | 0.318 | -0.160 | 0.032 | -0.314^*^ | -0.104 |
|  | (0.139) | (0.182) | (0.148) | (0.290) | (0.251) | (0.208) | (0.282) | (0.152) | (0.144) |
| Frailty dummy | 0.250 | 0.269 | 0.496^***^ | 0.050 | 0.273 | -0.074 | -0.248 | -0.279^*^ | 0.037 |
|  | (0.134) | (0.171) | (0.144) | (0.254) | (0.217) | (0.203) | (0.278) | (0.140) | (0.132) |
| Cognition score = 1 (ref. = 0) | -0.417 | 0.519 | 0.269 | 0.142 | -0.637 | -0.222 | -1.706 | -0.321 | 0.071 |
|  | (0.615) | (1.268) | (0.864) | (1.215) | (1.378) | (1.107) | (1.710) | (0.674) | (0.651) |
| Cognition score = 2 (ref. = 0) | -0.337 | 0.810 | 0.493 | 0.492 | -0.039 | 0.842 | 0.000 | 0.100 | 0.047 |
|  | (0.468) | (0.763) | (0.669) | (0.750) | (0.975) | (1.040) | (.) | (0.457) | (0.489) |
| Cognition score = 3 (ref. = 0) | -1.214^**^ | 0.126 | -0.355 | 0.068 | -0.558 | 0.582 | 0.536 | 0.397 | 0.018 |
|  | (0.405) | (0.633) | (0.540) | (0.259) | (0.815) | (0.851) | (0.755) | (0.388) | (0.421) |
| Cognition score = 4 (ref. = 0) | -1.460^***^ | -0.280 | -0.465 | 0.000 | -0.527 | 0.796 | 1.184 | 0.698 | 0.318 |
|  | (0.393) | (0.617) | (0.530) | (.) | (0.802) | (0.835) | (0.726) | (0.371) | (0.409) |
| Constant | -4.867 | 0.437 | 0.953 | -3.679 | -7.103 | -1.133 | -12.808^*^ | -2.175 | -1.812 |
|  | (3.065) | (3.360) | (2.833) | (4.283) | (3.753) | (4.563) | (5.263) | (3.089) | (2.849) |
| Country dummies | Yes | Yes | Yes | Yes | Yes | Yes | Yes | Yes | Yes |
| Interview week dummies | Yes | Yes | Yes | Yes | Yes | Yes | Yes | Yes | Yes |
| Interaction between dummies | Yes | Yes | Yes | Yes | Yes | Yes | Yes | Yes | Yes |
| N | 2,186 | 2,086 | 2,141 | 1,628 | 1,849 | 1,310 | 1,081 | 2,143 | 2,149 |
| Pseudo R2 | 0.255 | 0.172 | 0.234 | 0.102 | 0.133 | 0.370 | 0.143 | 0.086 | 0.084 |

Note: Logistic regressions based on the sample that was unable to stand up during the chair stand test; the table presents coefficients along with standard errors in parentheses; * p<0.05, ** p<0.01, *** p<0.0

Table A.15: Robustness analysis for underestimating health: controlling for public health measures at the time of the interview

|  | (1) | (2) | (3) | (4) | (5) | (6) | (7) | (8) | (9) |
| --- | --- | --- | --- | --- | --- | --- | --- | --- | --- |
|  | Staying home | Less shopping | Less walks | Less meetings | Less visits | Wearing masks | Keeping distance | Washing hands | Sanitising hands |
| Underestimating | 0.263^***^ | 0.200^***^ | 0.213^***^ | -0.063 | -0.048 | 0.069 | 0.088 | 0.047 | -0.033 |
|  | (0.066) | (0.059) | (0.053) | (0.088) | (0.071) | (0.082) | (0.133) | (0.073) | (0.062) |
| Age | -0.022 | -0.055 | -0.104^***^ | 0.189^***^ | 0.132^***^ | 0.123^**^ | 0.196^**^ | 0.191^***^ | 0.075^*^ |
|  | (0.039) | (0.031) | (0.030) | (0.047) | (0.037) | (0.046) | (0.061) | (0.036) | (0.033) |
| Age squared | 0.001^*^ | 0.001^*^ | 0.001^***^ | -0.001^***^ | -0.001^**^ | -0.001^**^ | -0.002^***^ | -0.002^***^ | -0.001^***^ |
|  | (0.000) | (0.000) | (0.000) | (0.000) | (0.000) | (0.000) | (0.000) | (0.000) | (0.000) |
| Women | 0.271^***^ | 0.638^***^ | 0.277^***^ | 0.425^***^ | 0.309^***^ | 0.415^***^ | 0.396^***^ | 0.231^***^ | 0.108^**^ |
|  | (0.046) | (0.033) | (0.032) | (0.052) | (0.041) | (0.046) | (0.078) | (0.045) | (0.039) |
| Medium education (ref. = low) | -0.330^***^ | -0.033 | -0.202^***^ | -0.026 | -0.074 | 0.179^**^ | 0.151 | 0.095 | 0.105^*^ |
|  | (0.057) | (0.046) | (0.043) | (0.070) | (0.059) | (0.066) | (0.097) | (0.058) | (0.053) |
| High education (ref. = low) | -0.653^***^ | 0.035 | -0.424^***^ | 0.165^*^ | -0.001 | 0.382^***^ | 0.466^***^ | 0.355^***^ | 0.146^*^ |
|  | (0.074) | (0.051) | (0.048) | (0.081) | (0.065) | (0.071) | (0.118) | (0.069) | (0.060) |
| Chronic disease dummy | 0.081 | 0.167^***^ | 0.240^***^ | 0.070 | 0.097^*^ | 0.239^***^ | 0.159 | -0.043 | 0.057 |
|  | (0.055) | (0.038) | (0.038) | (0.058) | (0.048) | (0.053) | (0.088) | (0.053) | (0.048) |
| ADL dummy | -0.080 | -0.002 | 0.212^*^ | 0.155 | 0.301^*^ | -0.075 | -0.266 | -0.052 | -0.005 |
|  | (0.105) | (0.089) | (0.083) | (0.141) | (0.121) | (0.120) | (0.184) | (0.103) | (0.091) |
| IADL dummy | 0.300^***^ | 0.144 | 0.238^***^ | 0.006 | 0.036 | -0.040 | -0.138 | -0.235^**^ | -0.069 |
|  | (0.083) | (0.075) | (0.070) | (0.116) | (0.094) | (0.100) | (0.163) | (0.085) | (0.079) |
| Frailty dummy | 0.465^***^ | 0.225^*^ | 0.641^***^ | 0.776^***^ | 0.311^*^ | 0.297^*^ | -0.185 | -0.090 | -0.280^**^ |
|  | (0.085) | (0.098) | (0.090) | (0.186) | (0.130) | (0.136) | (0.188) | (0.102) | (0.088) |
| Cognition score = 1 (ref. = 0) | -0.124 | 0.279 | -0.537 | 0.572 | -0.509 | -0.002 | -1.222 | -0.322 | -0.617 |
|  | (0.391) | (0.472) | (0.473) | (1.095) | (0.568) | (0.802) | (0.810) | (0.387) | (0.367) |
| Cognition score = 2 (ref. = 0) | -0.767^**^ | -0.150 | -0.575 | -0.409 | -0.121 | -0.187 | -0.560 | 0.279 | -0.061 |
|  | (0.271) | (0.285) | (0.312) | (0.432) | (0.388) | (0.463) | (0.593) | (0.274) | (0.270) |
| Cognition score = 3 (ref. = 0) | -0.939^***^ | -0.157 | -0.708^**^ | -0.338 | -0.311 | 0.104 | -0.137 | 0.676^**^ | 0.146 |
|  | (0.226) | (0.246) | (0.272) | (0.362) | (0.330) | (0.404) | (0.525) | (0.226) | (0.229) |
| Cognition score = 4 (ref. = 0) | -1.041^***^ | -0.173 | -0.755^**^ | -0.344 | -0.378 | 0.206 | -0.123 | 0.695^**^ | 0.251 |
|  | (0.221) | (0.243) | (0.270) | (0.357) | (0.327) | (0.399) | (0.517) | (0.221) | (0.226) |
| Mandatory mask use in all public spaces | -0.105 | -0.109 | -0.159 | 0.071 | -0.142 | 0.037 | 0.064 | -0.040 | -0.048 |
|  | (0.106) | (0.082) | (0.085) | (0.142) | (0.109) | (0.151) | (0.203) | (0.114) | (0.101) |
| Restrictions, private gatherings | 0.057 | -0.105 | 0.058 | 0.015 | 0.046 | 0.047 | 0.239 | 0.015 | -0.166 |
|  | (0.139) | (0.088) | (0.087) | (0.163) | (0.119) | (0.122) | (0.251) | (0.124) | (0.110) |
| Constant | -3.756^**^ | 1.018 | 1.762 | -4.633^**^ | -3.330^**^ | -2.010 | -2.649 | -4.647^***^ | -0.659 |
|  | (1.352) | (1.039) | (1.021) | (1.549) | (1.239) | (1.548) | (2.099) | (1.200) | (1.112) |
| Country dummies | Yes | Yes | Yes | Yes | Yes | Yes | Yes | Yes | Yes |
| Interview week dummies | Yes | Yes | Yes | Yes | Yes | Yes | Yes | Yes | Yes |
| N | 19,437 | 19,263 | 19,173 | 19,016 | 19,056 | 16,848 | 16,827 | 19,416 | 19,423 |
| Pseudo R2 | 0.179 | 0.094 | 0.201 | 0.078 | 0.061 | 0.486 | 0.045 | 0.035 | 0.069 |

Note: Logistic regressions based on the sample that was able to stand up during the chair stand test; the table presents coefficients along with standard errors in parentheses; * p<0.05, ** p<0.01, *** p<0.0

Table A.16: Robustness analysis for overestimating health: controlling for public health measures at the time of the interview

|  | (1) | (2) | (3) | (4) | (5) | (6) | (7) | (8) | (9) |
| --- | --- | --- | --- | --- | --- | --- | --- | --- | --- |
|  | Staying home | Less shopping | Less walks | Less meetings | Less visits | Wearing masks | Keeping distance | Washing hands | Sanitising hands |
| Overestimating | -0.004 | -0.043 | 0.040 | -0.229 | 0.132 | 0.245 | 0.770^**^ | -0.037 | 0.151 |
|  | (0.127) | (0.144) | (0.126) | (0.225) | (0.188) | (0.178) | (0.263) | (0.135) | (0.128) |
| Age | 0.010 | -0.077 | -0.085 | 0.073 | 0.238^*^ | 0.057 | 0.422^**^ | 0.140 | 0.110 |
|  | (0.086) | (0.101) | (0.084) | (0.142) | (0.111) | (0.131) | (0.143) | (0.087) | (0.080) |
| Age squared | 0.000 | 0.001 | 0.001 | -0.000 | -0.002 | -0.001 | -0.003^**^ | -0.001^*^ | -0.001^*^ |
|  | (0.001) | (0.001) | (0.001) | (0.001) | (0.001) | (0.001) | (0.001) | (0.001) | (0.001) |
| Women | 0.487^***^ | 0.695^***^ | 0.416^***^ | 0.350 | 0.189 | 0.585^***^ | 0.521^*^ | 0.419^***^ | 0.222 |
|  | (0.122) | (0.126) | (0.111) | (0.199) | (0.170) | (0.166) | (0.233) | (0.125) | (0.118) |
| Medium education (ref. = low) | -0.395^**^ | -0.088 | 0.064 | -0.107 | 0.127 | -0.209 | 0.391 | -0.271 | 0.202 |
|  | (0.135) | (0.149) | (0.127) | (0.233) | (0.199) | (0.194) | (0.249) | (0.149) | (0.136) |
| High education (ref. = low) | -0.618^***^ | 0.071 | -0.172 | 0.115 | -0.024 | 0.059 | 0.333 | -0.086 | 0.125 |
|  | (0.187) | (0.191) | (0.158) | (0.302) | (0.251) | (0.226) | (0.348) | (0.191) | (0.176) |
| Chronic disease dummy | -0.010 | 0.170 | 0.173 | 0.653^**^ | -0.005 | 0.320 | 0.112 | 0.202 | 0.158 |
|  | (0.166) | (0.173) | (0.155) | (0.238) | (0.245) | (0.215) | (0.304) | (0.177) | (0.169) |
| ADL dummy | 0.323^*^ | 0.379^*^ | 0.249 | 0.416 | 0.019 | 0.282 | 0.745^*^ | -0.072 | -0.181 |
|  | (0.142) | (0.190) | (0.160) | (0.310) | (0.240) | (0.227) | (0.319) | (0.144) | (0.142) |
| IADL dummy | 0.322^*^ | 0.383^*^ | 0.371^*^ | 0.028 | 0.093 | -0.169 | -0.103 | -0.328^*^ | -0.102 |
|  | (0.136) | (0.179) | (0.148) | (0.285) | (0.247) | (0.202) | (0.281) | (0.147) | (0.142) |
| Frailty dummy | 0.238 | 0.267 | 0.487^***^ | 0.011 | 0.373 | -0.135 | -0.079 | -0.311^*^ | 0.037 |
|  | (0.132) | (0.169) | (0.140) | (0.255) | (0.216) | (0.197) | (0.264) | (0.140) | (0.131) |
| Cognition score = 1 (ref. = 0) | -0.474 | 0.668 | 0.133 | 0.077 | -0.501 | -0.375 | -0.949 | -0.359 | 0.286 |
|  | (0.589) | (1.205) | (0.856) | (1.160) | (1.297) | (1.122) | (1.381) | (0.629) | (0.616) |
| Cognition score = 2 (ref. = 0) | -0.418 | 0.907 | 0.504 | 0.529 | 0.509 | 0.877 | 0.000 | 0.049 | 0.114 |
|  | (0.448) | (0.777) | (0.641) | (0.730) | (1.027) | (0.996) | (.) | (0.448) | (0.454) |
| Cognition score = 3 (ref. = 0) | -1.277^***^ | 0.024 | -0.465 | 0.088 | -0.530 | 0.495 | 0.498 | 0.314 | 0.089 |
|  | (0.387) | (0.571) | (0.506) | (0.258) | (0.753) | (0.814) | (0.743) | (0.385) | (0.392) |
| Cognition score = 4 (ref. = 0) | -1.459^***^ | -0.264 | -0.499 | 0.000 | -0.414 | 0.597 | 1.075 | 0.597 | 0.348 |
|  | (0.375) | (0.551) | (0.495) | (.) | (0.737) | (0.792) | (0.708) | (0.369) | (0.379) |
| Mandatory mask use in all public spaces | -0.433 | -0.569^*^ | -0.476 | 0.439 | 0.689 | -0.134 | 0.872 | -0.257 | -0.446 |
|  | (0.265) | (0.261) | (0.264) | (0.707) | (0.468) | (0.534) | (0.561) | (0.276) | (0.274) |
| Restrictions, private gatherings | -0.449 | -0.477 | -0.226 | 0.314 | -0.582 | -0.007 | 0.695 | 0.044 | 0.222 |
|  | (0.359) | (0.332) | (0.303) | (0.688) | (0.555) | (0.487) | (0.772) | (0.352) | (0.341) |
| Constant | -4.463 | 1.590 | 0.689 | -2.214 | -7.081 | 0.283 | -11.971^*^ | -2.915 | -1.995 |
|  | (2.994) | (3.316) | (2.815) | (4.604) | (3.724) | (4.373) | (4.940) | (2.974) | (2.735) |
| Country dummies | Yes | Yes | Yes | Yes | Yes | Yes | Yes | Yes | Yes |
| Interview week dummies | Yes | Yes | Yes | Yes | Yes | Yes | Yes | Yes | Yes |
| N | 2,182 | 2,141 | 2,144 | 2,040 | 2,118 | 1,505 | 1,393 | 2,172 | 2,175 |
| Pseudo R2 | 0.236 | 0.152 | 0.208 | 0.106 | 0.131 | 0.400 | 0.137 | 0.065 | 0.069 |

Note: Logistic regressions based on the sample that was unable to stand up during the chair stand test; the table presents coefficients along with standard errors in parentheses; * p<0.05, ** p<0.01, *** p<0.0
